# Supplementary material for: Multitrait GWAS to connect disease variants and biological mechanisms
Source: PLoS Genet. 2021 Aug 30;17(8):e1009713. doi: 10.1371/journal.pgen.1009713 (PMC8437297; doi:10.1371/journal.pgen.1009713)
Supplement: S1 Text — (DOCX) [file pgen.1009713.s001.docx]

Supplementary Text for

Multitrait GWAS to connect disease variants and biological mechanisms

Table of contents

[Supplementary Text for 1](#_Toc77688840)

[Multitrait GWAS to connect disease variants and biological mechanisms 1](#_Toc77688841)

[Multivariate statistical test 2](#_Toc77688842)

[Relationship between the proposed statistical tests 3](#_Toc77688843)

[Solving singular covariance matrix 3](#_Toc77688844)

[Theoretical comparison with MANOVA 4](#_Toc77688845)

[Validation of the approach using UK Biobank data 5](#_Toc77688846)

[Expected z-score covariance for null SNPs 5](#_Toc77688847)

[Simulation to assess the impact of causal variants on the covariance estimation 7](#_Toc77688848)

[Sample size inference for linear regression 7](#_Toc77688849)

[Sample size inference for logistic regression 8](#_Toc77688850)

[Imputation of missing z-score statistics 8](#_Toc77688851)

[Unbiased genetic covariance estimator by clusters 9](#_Toc77688852)

[Tissue enrichment analysis 10](#_Toc77688853)

**Supplementary Note**

## Multivariate statistical test

The *Omnibus* test is a standard generalization of Student's t-statistic to the multivariate case. It aims at testing the null hypothesis of no association between a single SNP and none of the outcomes. Briefly, assuming a vector of z-scores $\mathbf{z}$ of size $K$ follows a multivariate normal with mean 0 and covariance $\boldsymbol{\Sigma}_{\mathbf{r}}$, the probability that an occurrence $\mathbf{z}$ belong to the multivariate distribution can be estimated by comparing $T_{omni}$, the square of its *Mahalanobis* distance (a generalization of the mean to standard deviation distance), to a chi-squared distribution with $K$ degree of freedom (although note that for the special case of small sample size relative to the number of outcomes, alternative distribution should be used, see the section about MANOVA). In practice, $T_{omni}$ is defined as:

$$\boldsymbol{T}_{\boldsymbol{omni}}\mathbf{=}\mathbf{z}^{\boldsymbol{t}}\boldsymbol{\Sigma}_{\mathbf{r}}^{\mathbf{-1}}\mathbf{z}\boldsymbol{\sim}\boldsymbol{\chi}_{\boldsymbol{Kdf}}^{\boldsymbol{2}}$$

This test is similar to the previously published *metaCCA*[1] approach. However, the existing implementation of *metaCCA* does not address any of the practical issues discussed in the main text (data pre-processing, missing statistics, etc). It also shares similarity with TATES[2], which suffers the same practical issues as *metaCCA*. Moreover, *TATES* has a slightly different null hypothesis, based on the aggregation of *p*-value instead of signed statistics. Finally, both methods propose using an *ad hoc* estimation of the covariance between test under the null, which, has shown in S8 Fig, can be invalid.

The *sumZ* statistics consists in testing whether a specific linear combination of the individual univariate statistics follows a null distribution between the tested SNP and the outcomes. We defined a simple weighted sum of z-score, $S=\mathbf{w}^{t}\mathbf{z}$. Again, assuming$\boldsymbol{z\sim MVN}\left( \boldsymbol{0},\boldsymbol{\Sigma}_{\mathbf{r}} \right)$, where $\mathbf{MVN}$ is the multivariate normal distribution, the statistics $S$ being a sum of normally distributed variables, also follows a normal distribution with mean and variance equal to:

$$\mathbb{E}\left[ \mathbf{w}^{\boldsymbol{t}}\mathbf{z} \right]\mathbb{=E}\left[ \sum_{\boldsymbol{i}\mathbf{=1}}^{\boldsymbol{K}} \boldsymbol{w}_{\boldsymbol{i}}\boldsymbol{z}_{\boldsymbol{i}} \right]\mathbf{=}\sum_{\boldsymbol{i}\mathbf{=1}}^{\boldsymbol{K}} \boldsymbol{w}_{\boldsymbol{i}}\mathbb{E}\left[ \boldsymbol{z}_{\boldsymbol{i}} \right]\mathbf{=0}$$

$$\boldsymbol{var}\left[ \mathbf{w}^{\boldsymbol{t}}\mathbf{z} \right]\mathbf{=}\boldsymbol{var}\left[ \sum_{\boldsymbol{i}\mathbf{=1}}^{\boldsymbol{K}} \boldsymbol{w}_{\boldsymbol{i}}\boldsymbol{z}_{\boldsymbol{i}} \right]\mathbf{=}\sum_{\boldsymbol{i}\mathbf{=1}}^{\boldsymbol{K}} \sum_{\boldsymbol{j}\mathbf{=1}}^{\boldsymbol{K}} \boldsymbol{w}_{\boldsymbol{i}}\boldsymbol{w}_{\boldsymbol{j}}\boldsymbol{var}\left[ \boldsymbol{z}_{\boldsymbol{i}}\boldsymbol{z}_{\boldsymbol{j}} \right]\mathbf{=}\mathbf{w}^{\boldsymbol{t}}\boldsymbol{\Sigma}_{\mathbf{r}}\mathbf{w}$$

Test of association can thus be defined as a standard two-sided *t*-test, i.e.:

$$\boldsymbol{t}_{\boldsymbol{sumZ}}\mathbf{=}\frac{\mathbf{w}^{\boldsymbol{t}}\mathbf{z-}\mathbb{E}\left[ \mathbf{w}^{\boldsymbol{t}}\mathbf{z} \right]}{\sqrt{\boldsymbol{var}\left[ \mathbf{w}^{\boldsymbol{t}}\mathbf{z} \right]}}\mathbf{=}\frac{\mathbf{w}^{\boldsymbol{t}}\mathbf{z}}{\sqrt{\mathbf{w}^{\boldsymbol{t}}\boldsymbol{\Sigma}_{\mathbf{r}}\mathbf{w}}}$$

Or more commonly, to a 1 degree of freedom chi-squared test statistics:

$$\boldsymbol{T}_{\boldsymbol{sumZ}}\mathbf{=}\frac{\left( \mathbf{w}^{\boldsymbol{t}}\mathbf{z} \right)^{\mathbf{2}}}{\mathbf{w}^{\mathbf{t}}\boldsymbol{\Sigma}_{\mathbf{r}}\mathbf{w}}\boldsymbol{\sim}\boldsymbol{\chi}_{\boldsymbol{1}\boldsymbol{df}}^{\boldsymbol{2}}$$

Several previous publications presented weighted approaches with various flavors (e.g. [3-6]). For example the HIPO approach[3] proposes to optimize the weights by combining $\Sigma_{r}$ and $\boldsymbol{\Sigma}_{\mathbf{g}}$, the genetic and residual covariance matrix, respectively, while we considered them separately. Zhu et al proposed two alternative weighting schemes, referred to as *sHom* and *sHet*. The *sHom* approach[7] is very similar to the weighting scheme of **1**, assuming similar direction of genetic effect across the traits analyzed, and also accounting for sample size per GWAS. On the other hand, the *sHet* propose a binary weight (i.e. 0 or 1) allowing to include or exclude a subset of phenotype to be analyzed jointly. Finally, the *aSPU* test[5] and its extension (e.g. metaUSAT[6]) extend the latter *sHet* approach and proposes using a combination of multiple weights. While potentially powerful, the *ad hoc* combining assessing multiple weight requires estimating the null distribution of the statistics using a Monte Carlo simulation.

## Relationship between the proposed statistical tests

The two proposed statistics can be linked in the special case where the weights $\mathbf{w}$ equal $\mathbf{v}$, the eigenvector of $\boldsymbol{\Sigma}_{\mathbf{r}}$**:**

$$\boldsymbol{T}_{\boldsymbol{omni}}\mathbf{=}\sum_{\boldsymbol{i}\mathbf{=1}}^{\boldsymbol{K}} \boldsymbol{T}_{\boldsymbol{sumZ}}\left| \mathbf{w=} \right.\mathbf{v}_{\boldsymbol{i}}$$

*Proof:* Consider the $K\times K$ variance-covariance matrix $\boldsymbol{\Sigma}_{\mathbf{r}}$ of the z-scores. As $\boldsymbol{\Sigma}_{\mathbf{r}}$ is symmetric and using the spectral theorem, there exists an orthogonal matrix $\mathbf{P}$ and a diagonal matrix $\mathbf{D}$ such that:

$\boldsymbol{\Sigma}_{\mathbf{r}}\mathbf{=PD}\mathbf{P}^{\mathbf{t}}$

where the columns of $\mathbf{P}$ correspond to the eigenvectors $\mathbf{v}_{i=1\ldots K}$ of $\boldsymbol{\Sigma}_{\mathbf{r}}$ and the diagonal of $\mathbf{D}$ corresponds to the eigenvalues $\lambda_{i=1\ldots K}$ of $\boldsymbol{\Sigma}_{\mathbf{r}}$. The covariance matrix can then be further written as:

$$\boldsymbol{\Sigma}_{\mathbf{r}}\mathbf{=}\sum_{\boldsymbol{i}\mathbf{=1}}^{\boldsymbol{K}} \boldsymbol{\lambda}_{\boldsymbol{i}}\mathbf{v}_{\boldsymbol{i}}\mathbf{v}_{\boldsymbol{i}}^{\boldsymbol{t}}$$

It follows that the *Omnibus* test can be rewritten as:

$$\boldsymbol{T}_{\boldsymbol{omni}}\mathbf{=}\boldsymbol{z}^{\boldsymbol{t}}\boldsymbol{\Sigma}_{\boldsymbol{r}}^{\mathbf{-1}}\boldsymbol{z}$$

$$\mathbf{=}\boldsymbol{z}^{\boldsymbol{t}}\left( \boldsymbol{PD}\boldsymbol{P}^{\boldsymbol{t}} \right)^{\mathbf{-1}}\boldsymbol{z}$$

$$\mathbf{=}\boldsymbol{z}^{\boldsymbol{t}}\left( \boldsymbol{P}^{\boldsymbol{t}} \right)^{\mathbf{-1}}\boldsymbol{D}^{\mathbf{-1}}\boldsymbol{P}^{\mathbf{-1}}\boldsymbol{z}$$

$$\mathbf{=}\boldsymbol{z}^{\boldsymbol{t}}\boldsymbol{P}\boldsymbol{D}^{\mathbf{-1}}\boldsymbol{P}^{\boldsymbol{t}}\boldsymbol{z}$$

$$\mathbf{=}\mathbf{z}^{\boldsymbol{t}}\left( \sum_{\boldsymbol{i}\mathbf{=1}}^{\boldsymbol{K}} \frac{\mathbf{1}}{\boldsymbol{\lambda}_{\boldsymbol{i}}}\mathbf{v}_{\boldsymbol{i}}\mathbf{v}_{\boldsymbol{i}}^{\boldsymbol{t}} \right)\mathbf{z}$$

$$\mathbf{=}\sum_{\boldsymbol{i}\mathbf{=1}}^{\boldsymbol{K}} \frac{\mathbf{1}}{\boldsymbol{\lambda}_{\boldsymbol{i}}}\left( \mathbf{z}_{\boldsymbol{i}}^{\boldsymbol{t}}\mathbf{v}_{\boldsymbol{i}} \right)\left( \mathbf{z}_{\boldsymbol{i}}^{\boldsymbol{t}}\mathbf{v}_{\boldsymbol{i}} \right)^{\boldsymbol{t}}$$

$$\mathbf{=}\sum_{\boldsymbol{i}\mathbf{=1}}^{\boldsymbol{K}} \frac{\mathbf{1}}{\boldsymbol{\lambda}_{\boldsymbol{i}}}\left( \mathbf{z}_{\boldsymbol{i}}^{\boldsymbol{t}}\mathbf{v}_{\boldsymbol{i}} \right)^{\mathbf{2}}$$

On the other hand, applying the *sumZ* statistic with weights $\mathbf{w=}\mathbf{v}_{i}$, where $\mathbf{v}_{i}$ is the eigenvector $i$ of $\boldsymbol{\Sigma}_{\mathbf{r}}$ corresponding to $\lambda_{i}$,we have:

$$\boldsymbol{T}_{\boldsymbol{sumZ}}\mathbf{=}\frac{\left( \mathbf{v}_{\boldsymbol{i}}^{\boldsymbol{t}}\mathbf{z} \right)^{\mathbf{2}}}{\mathbf{v}_{\boldsymbol{i}}^{\boldsymbol{t}}\boldsymbol{\Sigma}_{\mathbf{r}}\mathbf{v}_{\mathbf{i}}}$$

As $\mathbf{v}_{i}$ is a principal component of $\boldsymbol{\Sigma}_{\mathbf{r}}$, by definition we have $\mathbf{v}_{i}^{t}\boldsymbol{\Sigma}_{\mathbf{r}}\mathbf{v}_{i}\boldsymbol{=}\lambda_{i}\mathbf{v}_{i}^{t}\mathbf{v}_{i}$, but also $\mathbf{v}_{i}^{t}\mathbf{v}_{i}\boldsymbol{=}1$. It follows that:

$$\boldsymbol{T}_{\boldsymbol{sumZ}}\mathbf{=}\frac{\mathbf{1}}{\boldsymbol{\lambda}_{\boldsymbol{i}}}\left( \mathbf{v}_{\boldsymbol{i}}^{\boldsymbol{t}}\mathbf{z} \right)^{\mathbf{2}}$$

## Solving singular covariance matrix

Consider the case where the estimated variance-covariance matrix ${\hat{\boldsymbol{\Sigma}}}_{\mathbf{r}}$ is not invertible. We compared the performances of three methods described below to derive a pseudo-inverse of ${\hat{\boldsymbol{\Sigma}}}_{\mathbf{r}}$. Using the spectral theorem, ${\hat{\boldsymbol{\Sigma}}}_{\mathbf{r}}$ can be written:

$${\hat{\boldsymbol{\Sigma}}}_{\mathbf{r}}\mathbf{=PD}\mathbf{P}^{\boldsymbol{t}}$$

where $\mathbf{D}=diag\left( \left( \lambda_{k} \right)_{k=1\ldots K} \right)$, $\left( \lambda_{k} \right)_{k=1\ldots K}$ are the eigenvalues of ${\hat{\boldsymbol{\Sigma}}}_{\mathbf{r}}$ and $\mathbf{P}$ is the orthogonal matrix which columns correspond to the eigenvectors of ${\hat{\boldsymbol{\Sigma}}}_{\mathbf{r}}$.

***Strategy 1:*** The first strategy consists in considering only eigenvalues greater than a specified threshold $\epsilon$. Suppose only $K^{'}$ eigenvalues out of $K$ are greater than $\epsilon$. Let $D_{K^{'}}=diag\left( \left( \lambda_{k} \right)_{k=1\ldots K^{'}} \right)$ and $\mathbf{P}_{K^{'}}$ denote the $K\times K^{'}$ matrix which columns are the $K^{'}$ eigenvectors corresponding to the eigenvalues greater than $\epsilon$. We then define the pseudo-inverse of ${\hat{\boldsymbol{\Sigma}}}_{\mathbf{r}}$ by:

$${\hat{\boldsymbol{\Sigma}}}_{\mathbf{r}}^{\mathbf{*}}\mathbf{=}\mathbf{P}_{\boldsymbol{K}^{\mathbf{'}}}\mathbf{D}_{\boldsymbol{K}^{\mathbf{'}}}^{\mathbf{-1}}\mathbf{P}_{\boldsymbol{K}^{\mathbf{'}}}^{\boldsymbol{t}}$$

***Strategy 2:*** The second strategy is relatively similar to the first one. Instead of considering only the eigenvalues greater than a threshold $\epsilon$, eigenvalues below this threshold are set to $\epsilon$. Let $\mathbf{D}_{\epsilon}=diag\left( \left( \lambda_{k}^{'} \right)_{k=1\ldots K^{'}} \right)$ where $\lambda_{k}^{'}$ is equal to $\lambda_{k}$ if $\lambda_{k}$ is greater than $\epsilon$ and $\epsilon$ otherwise. We then define the pseudo-inverse of ${\hat{\boldsymbol{\Sigma}}}_{\mathbf{r}}$ by:

$${\hat{\boldsymbol{\Sigma}}}_{\mathbf{r}}^{\mathbf{*}}\mathbf{=P}\mathbf{D}_{\boldsymbol{\epsilon}}^{\mathbf{-1}}\mathbf{P}^{\boldsymbol{t}}$$

***Strategy 3:*** The third strategy consists in adding a small value $\epsilon$ to the diagonal terms of ${\hat{\boldsymbol{\Sigma}}}_{\mathbf{r}}$. The pseudo-inverse of ${\hat{\boldsymbol{\Sigma}}}_{\mathbf{r}}$ is defined by:

$${\hat{\boldsymbol{\Sigma}}}_{\mathbf{r}}^{\mathbf{*}}\mathbf{=}\left( {\hat{\boldsymbol{\Sigma}}}_{\mathbf{r}}\mathbf{+}\boldsymbol{\epsilon}\boldsymbol{\times}\mathbf{I}_{\boldsymbol{K}} \right)^{\mathbf{-1}}$$

where $\mathbf{I}_{K}$ denote the $K\times K$ identity matrix.

We conducted simulation series to compare the relative performances of the three strategies for three different thresholds ($\epsilon=1\times{10}^{-3},1\times{10}^{-6}, 1\times{10}^{-9})$. As showed in **S1 Fig,** the distributions of the *p*-values were correctly calibrated for all three tests for the two highest thresholds, but remain correct only for strategy 1 for the lowest $\epsilon$. The latter strategy was therefore used in all analyses, as implemented in the JASS python package[8].

## Theoretical comparison with MANOVA

In the special case of complete sample overlap and no missing phenotypic value, the proposed *Omnibus* statistics is asymptotically similar to the one-way MANOVA. Consider $K$ correlated traits $Y_{k}$, $k=1,\ldots,K$ and let $\mathbf{Y}$ denote the $n\times K$ matrix of traits for all $n$ individuals. For a given variant, let $\mathbf{x}$ be $n\times1$ vector of predictors. The underlying model in the MANOVA model can be written as:

$$\mathbf{Y=x}\boldsymbol{\beta}^{\mathbf{t}}\mathbf{+}\boldsymbol{\varepsilon}$$

where $\boldsymbol{\beta}$ is the $1\times K$ vector of genetic effect on the $K$ phenotypes and $\varepsilon$ is the matrix of errors. As for the proposed *Omnibus* test, the null hypothesis tested in the MANOVA is $\boldsymbol{\beta}=0$, and it does not rely on an assumption about the direction of the effects. The Wilk’s Lambda test statistic is defined as follows:

$$\boldsymbol{W}\mathbf{=}\frac{\boldsymbol{det}\left( \mathbf{E} \right)}{\boldsymbol{det}\left( \mathbf{H+E} \right)}\mathbf{=}\frac{\boldsymbol{det}\left( \boldsymbol{Y}^{\boldsymbol{t}}\boldsymbol{Y}\mathbf{-}\hat{\boldsymbol{\beta}}\left( \mathbf{x}^{\boldsymbol{t}}\mathbf{x} \right){\hat{\boldsymbol{\beta}}}^{\boldsymbol{t}} \right)}{\boldsymbol{det}\left( \mathbf{Y}^{\boldsymbol{t}}\mathbf{Y} \right)}$$

where $\mathbf{E}=\mathbf{Y}^{t}\mathbf{Y}-\hat{\boldsymbol{\beta}}\left( \mathbf{x}^{t}\mathbf{x} \right){\hat{\boldsymbol{\beta}}}^{t}$, $\mathbf{H}=\hat{\boldsymbol{\beta}}\left( \mathbf{x}^{t}\mathbf{x} \right){\hat{\boldsymbol{\beta}}}^{t}$ and $\hat{\boldsymbol{\beta}}=\mathbf{Y}^{t}\mathbf{x}\left( \mathbf{x}^{T}\mathbf{x} \right)^{-1}$. Under the null, $W$ follows a Wilks’ lambda distribution $\Lambda\left( K, n-1, 1 \right)$. In practice, the statistical test is commonly performed using a *Fisher* distribution through the approximation $\frac{1-\Lambda\left( K, n-1, 1 \right)}{\Lambda\left( K, n-1, 1 \right)}\sim\frac{K}{n-K}F_{K,n-K}$ where $F_{p,q}$ denotes the Fisher distribution with $p$ and $q$ degrees of freedom. Furthermore, note that when $n$ is large compared to $K$, the quantity $\left( \frac{K}{2}-n+1 \right)\log\left( W \right)$ can be approximated by a chi-square distribution with $K$ degrees of freedom under the null. More details on *Wilks’* lambda distribution and its approximation can be found in Mardia et al.[9] and Bartlett[10]. Importantly, the Fisher approximation of the *Wilks’* statistics can be directly derived using only summary statistics since one can use the phenotypic variance-covariance matrix derived using the LD Score to estimate the matrix $\mathbf{Y}^{t}\mathbf{Y}$, while $\hat{\boldsymbol{\beta}}\left( \mathbf{x}^{t}\mathbf{x} \right){\hat{\boldsymbol{\beta}}}^{t}$ can be estimated by $\mathbf{z}^{t}\mathbf{z}$. Nevertheless, although the null hypothesis tested in the MANOVA and the *Omnibus* test is the same, a direct theoretical comparison of the two approaches is non-trivial. Instead, we performed series of simulation studies (**Figs S4** and **S5**) to assess the relative performances of these approaches.

Overall, all analyses confirm the three tests show similar results as long as $K\ll n$ as it is the case in large consortia and most of recent GWAS. In the special case where $n$ is relatively close to $K$ (see e.g. **Figs S4c** and **S5c,** where $K$=100 and $N$=500), the *Omnibus* test shows deflated statistics and therefore, decreased power as compared to the MANOVA. For such settings, we recommend using the Fisher approximation, which shows improved adequacy with the MANOVA. Finally, for the special case when $n$ is relatively close to $K$, but outcomes have only partial sample overlap, there are no trivial solution and further development is therefore required.

## Validation of the approach using UK Biobank data

We used data from the UK Biobank cohort[11, 12] to validate our approach and our theoretical comparison between the *Omnibus* approach and the MANOVA. In brief, we selected five anthropometric traits and 619,017 high-quality genotyped SNPs with minor allele frequencies (MAF) > 1% available in a subset of 336,347 unrelated individuals of British ancestry. For ease of comparison, we projected out all relevant covariates and used the residual as primary outcomes in all individual-level data analyses.

As showed in **Fig S7** and **S8**, a correct estimation of the covariance matrix between GWAS summary statistics under the null is critical and can be impacted by true genetic effects. We first aimed at confirming the validity of the LDSC estimator. We performed multiple analyses while varying the sample size use to generate summary statistics by randomly sub-sampling the complete data. In a first analysis, we down-sampled the whole dataset by removing 50%, 90% and 99% of all individuals; the resulted sample size was 168,173, 33,635 and 3,363 individuals, respectively. In a second analysis, we down-sampled the individuals but for a single trait at the same rates (i.e. removing 50%, 90% and 99% of individuals), thus inducing different sample overlaps between the down-sampled trait and other traits. In all scenarios considered, we observed a very strong concordance between the LDSC estimates and the expected value derived using the estimated phenotypic correlation and the true sample size overlap (**S9 Fig**), demonstrating the adequacy of the LDSC regression models, at least for the anthropometric traits used in our analysis which are known to show high polygenicity[13].

For the comparison with the MANOVA, we ran its implementation in PLINK[14]. For the *Omnibus* test, we proceeded in three steps: (1) compute single-trait GWAS for each of the five anthropometric traits and store the summary statistics; (2) estimate the phenotypic correlation matrix by LDSC[15]; and (3) computed the *Omnibus* test to get multi-trait statistics. Looking at differences between -log10(*p*-values) from MANOVA and the *Omnibus* for a subset of 14,718 genotyped SNPs on Chromosome 20, we observed almost perfect correlation between the two approaches (**S6 Fig**). Note that once the individual phenotype summary statistics are derived, the computation time of MANOVA was considerably larger than for the *Omnibus* test (approximately 50 min *vs.* 5 min).

## Expected z-score covariance for null SNPs

Consider two standardized quantitative phenotypes $\mathbf{y}_{1}$ and $\mathbf{y}_{2}$ that have been tested for association with a set of $m$ single nucleotide polymorphisms (SNP) in studies with sample size $n_{1}$ and $n_{2}$, respectively, and $n_{s}$ samples shared between the two studies. We aim at estimating the expected covariance between the resulting z-score statistics for a given null predictor –i.e. for a putative variant drawn independently from others SNPs and not associated with the outcomes under study. We denote $\mathbf{x}_{1}$ and $\mathbf{x}_{2}$ the column vectors of standardized predictors for such a null variants in dataset 1 and 2. We can write $\mathbb{E}\left[ z_{1}z_{2} \right]$, the expected covariance between the z-scores at for $\mathbf{x}_{1}$ and $\mathbf{x}_{2}$, as:

$$\mathbb{E}\left[ \boldsymbol{z}_{\mathbf{1}}\boldsymbol{z}_{\mathbf{2}} \right]\mathbb{=E}\left[ \mathbb{E}\left[ \frac{\left( \left( \mathbf{x}_{\mathbf{1}}^{\boldsymbol{t}}\mathbf{x}_{\mathbf{1}} \right)^{\mathbf{-1}}\mathbf{x}_{\mathbf{1}}^{\boldsymbol{t}}\mathbf{y}_{\mathbf{1}} \right)}{\sqrt{\left( \mathbf{x}_{\mathbf{1}}^{\boldsymbol{t}}\mathbf{x}_{\mathbf{1}} \right)^{\mathbf{-1}}}}\left( \frac{\left( \left( \mathbf{x}_{\mathbf{2}}^{\boldsymbol{t}}\mathbf{x}_{\mathbf{2}} \right)^{\mathbf{-1}}\mathbf{x}_{\mathbf{2}}^{\boldsymbol{t}}\mathbf{y}_{\mathbf{2}} \right)}{\sqrt{\left( \mathbf{x}_{\mathbf{2}}^{\boldsymbol{t}}\mathbf{x}_{\boldsymbol{2}} \right)^{\mathbf{-1}}}} \right)^{\boldsymbol{t}}\mathbf{|}\mathbf{x}_{\boldsymbol{1}}\mathbf{,}\mathbf{x}_{\mathbf{2}}\mathbf{,}\mathbf{y}_{\mathbf{1}}\mathbf{,}\mathbf{y}_{\mathbf{2}} \right] \right]$$

$$\mathbb{=E}\left[ \mathbb{E}\left[ \frac{\mathbf{x}_{\mathbf{1}}^{\boldsymbol{t}}\mathbf{y}_{\mathbf{1}}\left( \mathbf{x}_{\mathbf{2}}^{\boldsymbol{t}}\mathbf{y}_{\mathbf{2}} \right)^{\boldsymbol{t}}}{\sqrt{\boldsymbol{n}_{\mathbf{1}}}\sqrt{\boldsymbol{n}_{\mathbf{2}}}}\mathbf{|}\mathbf{x}_{\boldsymbol{1}}\mathbf{,}\mathbf{x}_{\mathbf{2}}\mathbf{,}\mathbf{y}_{\mathbf{1}}\mathbf{,}\mathbf{y}_{\mathbf{2}} \right] \right]$$

$$\mathbf{=}\frac{\mathbf{1}}{\sqrt{\boldsymbol{n}_{\mathbf{1}}\boldsymbol{n}_{\mathbf{2}}}}\mathbb{E}\left[ \mathbf{x}_{\mathbf{1}}^{\boldsymbol{t}}\mathbf{y}_{\mathbf{1}}\mathbf{y}_{\mathbf{2}}^{\boldsymbol{t}}\mathbf{x}_{\mathbf{2}} \right]$$

$$\mathbf{=}\frac{\boldsymbol{\rho}\boldsymbol{n}_{\boldsymbol{s}}}{\sqrt{\boldsymbol{n}_{\mathbf{1}}\boldsymbol{n}_{\mathbf{2}}}}$$

where $\rho$ is the phenotypic covariance derived over the $n_{s}$ overlapping samples.

Note that this is in agreement with recent work on the LD-score regression by Bulik-Sullivan et al[15]. Indeed, consider further that $\mathbf{y}_{1}\boldsymbol{=}\mathbf{G}_{1}\boldsymbol{\beta}_{1}+\boldsymbol{\varepsilon}_{1}$, and $\mathbf{y}_{2}\boldsymbol{=}\mathbf{G}_{2}\boldsymbol{\beta}_{2}+\boldsymbol{\varepsilon}_{2}$, where $\mathbf{G}_{1}$ and $\mathbf{G}_{2}$ are $n_{1}\boldsymbol{\times}m$ and $n_{2}\boldsymbol{\times}m$ matrices of standardized genotypes for the causal variants of outcome 1 and outcome 2, respectively, and $\boldsymbol{\varepsilon}_{1}$ and $\boldsymbol{\varepsilon}_{2}$ are there residual variance. Now assume the effects of $\mathbf{G}_{1}$ and $\mathbf{G}_{2}$ are distributed as $\boldsymbol{\beta}_{1}\mathcal{\sim N}\left( 0,{h_{1}^{2}}/m \right)$ and $\boldsymbol{\beta}_{2}\mathcal{\sim N}\left( 0,{h_{2}^{2}}/m \right)$, where $\boldsymbol{\beta}_{1}$ and $\boldsymbol{\beta}_{2}$ are two column vectors of size $m$ with $cov\left( \boldsymbol{\beta}_{1},\boldsymbol{\beta}_{2} \right)=\sigma_{g}=r_{g}{\sqrt{h_{1}^{2}h_{2}^{2}}}/m$, and that $\mathbf{y}_{1}$ and $\mathbf{y}_{2}$ also shared non-genetic variance, equals to $\sigma_{e}$. We can further re-write $\mathbb{E}\left[ z_{1}z_{2} \right]$ as:

$$\mathbb{E}\left[ \boldsymbol{z}_{\mathbf{1}}\boldsymbol{z}_{\mathbf{2}} \right]\mathbf{=}\frac{\mathbf{1}}{\sqrt{\boldsymbol{n}_{\mathbf{1}}\boldsymbol{n}_{\mathbf{2}}}}\mathbb{E}\left[ \mathbf{x}_{\mathbf{1}}^{\boldsymbol{t}}\mathbb{E}\left[ \left( \mathbf{G}_{\mathbf{1}}\boldsymbol{\beta}_{\mathbf{1}}\mathbf{+}\boldsymbol{\varepsilon}_{\mathbf{1}} \right)\left( \mathbf{G}_{\mathbf{2}}\boldsymbol{\beta}_{\mathbf{2}}\mathbf{+}\boldsymbol{\varepsilon}_{\mathbf{2}} \right)^{\boldsymbol{t}}\mathbf{|}\mathbf{x}_{\boldsymbol{1}}\mathbf{,}\mathbf{x}_{\mathbf{2}}\mathbf{,}\mathbf{G}_{\mathbf{1}}\mathbf{,}\mathbf{G}_{\mathbf{2}} \right]\mathbf{x}_{\mathbf{2}} \right]$$

$$\mathbf{=}\frac{\mathbf{1}}{\sqrt{\boldsymbol{n}_{\mathbf{1}}\boldsymbol{n}_{\mathbf{2}}}}\mathbb{E}\left[ \mathbf{x}_{\mathbf{1}}^{\boldsymbol{t}}\mathbb{E}\left[ \left( \mathbf{G}_{\mathbf{1}}\boldsymbol{\beta}_{\mathbf{1}}\mathbf{+}\boldsymbol{\varepsilon}_{\mathbf{1}} \right)\left( \boldsymbol{\beta}_{\mathbf{2}}^{\boldsymbol{t}}\mathbf{G}_{\mathbf{2}}^{\boldsymbol{t}}\mathbf{+}\boldsymbol{\varepsilon}_{\mathbf{2}}^{\boldsymbol{t}} \right)\mathbf{|}\mathbf{x}_{\boldsymbol{1}}\mathbf{,}\mathbf{x}_{\mathbf{2}}\mathbf{,}\mathbf{G}_{\mathbf{1}}\mathbf{,}\mathbf{G}_{\mathbf{2}} \right]\mathbf{x}_{\mathbf{2}} \right]$$

$$\mathbf{=}\frac{\mathbf{1}}{\sqrt{\boldsymbol{n}_{\mathbf{1}}\boldsymbol{n}_{\mathbf{2}}}}\mathbb{E}\left[ \mathbf{x}_{\mathbf{1}}^{\boldsymbol{t}}\left( \mathbf{G}_{\mathbf{1}}\mathbb{E}\left[ \left( \boldsymbol{\beta}_{\mathbf{1}}\boldsymbol{\beta}_{\mathbf{2}}^{\boldsymbol{t}} \right) \right]\mathbf{G}_{\mathbf{2}}^{\boldsymbol{t}}\mathbb{+E}\left[ \boldsymbol{\varepsilon}_{\mathbf{1}}\boldsymbol{\beta}_{\mathbf{2}}^{\boldsymbol{t}}\boldsymbol{G}_{\mathbf{2}}^{\boldsymbol{t}} \right]\mathbb{+E}\left[ \mathbf{G}_{\mathbf{1}}\boldsymbol{\beta}_{\mathbf{1}}\boldsymbol{\varepsilon}_{\mathbf{2}}^{\boldsymbol{t}} \right]\mathbb{+E}\left[ \boldsymbol{\varepsilon}_{\mathbf{1}}\boldsymbol{\varepsilon}_{\mathbf{2}}^{\boldsymbol{t}} \right] \right)\mathbf{x}_{\mathbf{2}} \right]$$

$$\mathbf{=}\frac{\mathbf{1}}{\sqrt{\boldsymbol{n}_{\mathbf{1}}\boldsymbol{n}_{\mathbf{2}}}}\mathbb{E}\left[ \mathbf{x}_{\mathbf{1}}^{\boldsymbol{t}}\mathbf{G}_{\mathbf{1}}\mathbb{E}\left[ \left( \boldsymbol{\beta}_{\mathbf{1}}\boldsymbol{\beta}_{\mathbf{2}}^{\boldsymbol{t}} \right) \right]\mathbf{G}_{\mathbf{2}}^{\boldsymbol{t}}\mathbf{x}_{\mathbf{2}} \right]\boldsymbol{+}\frac{\mathbf{1}}{\sqrt{\boldsymbol{n}_{\mathbf{1}}\boldsymbol{n}_{\mathbf{2}}}}\mathbb{E}\left[ \mathbf{x}_{\mathbf{1}}^{\boldsymbol{t}}\mathbb{E}\left[ \boldsymbol{\varepsilon}_{\mathbf{1}}\boldsymbol{\varepsilon}_{\mathbf{2}}^{\boldsymbol{t}} \right]\mathbf{x}_{\mathbf{2}} \right]$$

$$\mathbf{=}\frac{\mathbf{1}}{\sqrt{\boldsymbol{n}_{\mathbf{1}}\boldsymbol{n}_{\mathbf{2}}}}\mathbb{E}\left[ \mathbf{x}_{\mathbf{1}}^{\boldsymbol{t}}\mathbf{G}_{\mathbf{1}}\frac{\boldsymbol{\sigma}_{\boldsymbol{g}}}{\boldsymbol{m}}\mathbf{I}_{\boldsymbol{m}}\mathbf{G}_{\mathbf{2}}^{\boldsymbol{t}}\mathbf{x}_{\mathbf{2}} \right]\boldsymbol{+}\frac{\mathbf{1}}{\sqrt{\boldsymbol{n}_{\mathbf{1}}\boldsymbol{n}_{\mathbf{2}}}}\boldsymbol{\sigma}_{\boldsymbol{e}}\boldsymbol{n}_{\boldsymbol{s}}$$

$$\mathbf{=}\frac{\mathbf{1}}{\sqrt{\boldsymbol{n}_{\mathbf{1}}\boldsymbol{n}_{\mathbf{2}}}}\frac{\boldsymbol{\sigma}_{\boldsymbol{g}}}{\boldsymbol{m}}\mathbb{E}\left[ \mathbf{x}_{\mathbf{1}}^{\boldsymbol{t}}\mathbf{G}_{\mathbf{1}}\mathbf{G}_{\mathbf{2}}^{\boldsymbol{t}}\mathbf{x}_{\mathbf{2}} \right]\boldsymbol{+}\frac{\boldsymbol{\sigma}_{\boldsymbol{e}}\boldsymbol{n}_{\boldsymbol{s}}}{\sqrt{\boldsymbol{n}_{\mathbf{1}}\boldsymbol{n}_{\mathbf{2}}}}$$

Furthermore, we have $\mathbb{E}\left[ \mathbf{x}_{1i}^{t}\mathbf{G}_{1}\mathbf{G}_{2}^{t}\mathbf{x}_{2} \right]=\mathbb{E}\left[ \left( \mathbf{r}n_{1}+\boldsymbol{\theta}_{1} \right)^{t}\left( \mathbf{r}n_{2}+\boldsymbol{\theta}_{2} \right) \right],$ where $\mathbf{r}$ is a vector of the true correlation between the $\mathbf{x}_{\boldsymbol{.}}$ and all $m$ SNPs from $\mathbf{G}_{.}$, and $\boldsymbol{\theta}_{1}$ and $\boldsymbol{\theta}_{2}$ are the residuals noise due to finite sample size. Now, as compared to the derivation from Bulik-Sullivan et al[15], we consider here the special case where $\mathbf{x}_{1}$ and $\mathbf{x}_{2}$ are not correlated with any causal (i.e. independent of $\mathbf{G}_{1}$ and $\mathbf{G}_{2}$, respectively), so that $\mathbf{r=0}$ and $\mathbb{E}\left[ \mathbf{x}_{1i}^{t}\mathbf{G}_{1}\mathbf{G}_{2}^{t}\mathbf{x}_{2} \right]=\mathbb{E}\left[ \boldsymbol{\theta}_{1}^{t}\boldsymbol{\theta}_{2} \right]$. In the special case of no sample overlap, $\boldsymbol{\theta}_{1}$ and $\boldsymbol{\theta}_{2}$ are independent, so that the latter expectation is null. However, when there is sample overlap, subsets of $\mathbf{x}_{1}$ and $\mathbf{x}_{2}$, as well as $\mathbf{G}_{1}$ and $\mathbf{G}_{2}$, will be identical. We refer to these group as $\mathbf{x}_{s}$ and $\mathbf{G}_{s}$, respectively, and to $\mathbf{x}_{\boldsymbol{1}}^{\boldsymbol{*}}$, $\mathbf{x}_{\boldsymbol{2}}^{\boldsymbol{*}}$, $\mathbf{G}_{\boldsymbol{1}}^{\boldsymbol{*}}$,and $\mathbf{G}_{\boldsymbol{2}}^{\boldsymbol{*}}$, for their respective complements. We have now:

$$\mathbb{E}\left[ \boldsymbol{\theta}_{\boldsymbol{1}}^{\boldsymbol{t}}\boldsymbol{\theta}_{\boldsymbol{2}} \right]\boldsymbol{=}\mathbb{E}\left[ \left( \sum_{\boldsymbol{i\in}\boldsymbol{n}_{\boldsymbol{s}}} \mathbf{x}_{\mathbf{s}\boldsymbol{i}}^{\boldsymbol{t}}\mathbf{G}_{\boldsymbol{si}}\boldsymbol{+}\sum_{\boldsymbol{i\notin}\boldsymbol{n}_{\boldsymbol{s}}} \mathbf{x}_{\mathbf{1}\boldsymbol{i}}^{\boldsymbol{*t}}\mathbf{G}_{\boldsymbol{1}\boldsymbol{i}}^{\boldsymbol{*}} \right)^{\boldsymbol{t}}\left( \sum_{\boldsymbol{j\in}\boldsymbol{n}_{\boldsymbol{s}}} \mathbf{x}_{\mathbf{s}\boldsymbol{j}}^{\boldsymbol{t}}\mathbf{G}_{\boldsymbol{sj}}\boldsymbol{+}\sum_{\boldsymbol{j\notin}\boldsymbol{n}_{\boldsymbol{s}}} \mathbf{x}_{\mathbf{2}\boldsymbol{j}}^{\boldsymbol{*t}}\mathbf{G}_{\boldsymbol{2}\boldsymbol{j}}^{\boldsymbol{*}} \right) \right]$$

$$\boldsymbol{=}\mathbb{E}\left[ \left( \sum_{\boldsymbol{i\in}\boldsymbol{n}_{\boldsymbol{s}}} \mathbf{x}_{\mathbf{s}\boldsymbol{i}}^{\boldsymbol{t}}\mathbf{G}_{\mathbf{s}} \right)^{\boldsymbol{t}}\left( \sum_{\boldsymbol{i\in}\boldsymbol{n}_{\boldsymbol{s}}} \mathbf{x}_{\mathbf{s}\boldsymbol{i}}^{\boldsymbol{t}}\mathbf{G}_{\mathbf{s}} \right) \right]$$

$$\boldsymbol{=}\mathbb{E}\left[ \sum_{\boldsymbol{i\in}\boldsymbol{n}_{\boldsymbol{s}}} \left( \mathbf{x}_{\mathbf{s}\boldsymbol{i}}^{\boldsymbol{t}}\mathbf{G}_{\boldsymbol{si}} \right)^{\boldsymbol{2}} \right]$$

$$\boldsymbol{=}\mathbb{E}\left[ \sum_{\boldsymbol{i\in}\boldsymbol{n}_{\boldsymbol{s}}} \boldsymbol{m} \right]$$

$$\boldsymbol{=}\boldsymbol{n}_{\boldsymbol{s}}\boldsymbol{m}$$

It follows that we obtain again:

$$\mathbb{E}\left[ \boldsymbol{z}_{\boldsymbol{1}}\boldsymbol{z}_{\boldsymbol{2}} \right] \mathbf{=}\frac{\mathbf{1}}{\sqrt{\boldsymbol{n}_{\mathbf{1}}\boldsymbol{n}_{\mathbf{2}}}}\frac{\boldsymbol{\sigma}_{\boldsymbol{g}}}{\boldsymbol{m}}\left( \boldsymbol{n}_{\boldsymbol{s}}\boldsymbol{m} \right)\boldsymbol{+}\frac{\boldsymbol{\sigma}_{\boldsymbol{e}}\boldsymbol{n}_{\boldsymbol{s}}}{\sqrt{\boldsymbol{n}_{\mathbf{1}}\boldsymbol{n}_{\mathbf{2}}}}\mathbf{=}\frac{\left( \boldsymbol{\sigma}_{\boldsymbol{g}}\mathbf{+}\boldsymbol{\sigma}_{\boldsymbol{e}} \right)\boldsymbol{n}_{\boldsymbol{s}}}{\sqrt{\boldsymbol{n}_{\mathbf{1}}\boldsymbol{n}_{\mathbf{2}}}}\mathbf{=}\frac{\boldsymbol{\rho}\boldsymbol{N}_{\boldsymbol{s}}}{\sqrt{\boldsymbol{n}_{\mathbf{1}}\boldsymbol{n}_{\mathbf{2}}}}$$

## Simulation to assess the impact of causal variants on the covariance estimation

To generate results of **S8 Fig**, We simulated a series of 100 replicates each including 100,000 SNPs genotyped for 50,000 individuals. Coding allele frequency was uniformly sampled from [0.05, 0.95] and all SNPs were generated independently. For each replicate we generated two phenotypes with heritability drawn uniformly from [0.1, 0.75] and considered genetic correlation $r_{g}$ in [0.05, 0.5, 0.8]. We set the shared environmental variance $r_{e}$ so that the overall phenotypic correlation $r=r_{g}+r_{e}$ is equal to 0.6. With those parameters settings the phenotypic correlation can vary from high heritability, with $r$ fully explained by the genetic correlation to low heritability, with $r$ fully explained by the shared environment. We assumed only a small number $N_{c}$ of the total number of SNPs to be causal with effect sizes drawn from the multivariate normal distribution with mean 0 and covariance $\sigma_{g}=r_{g}{\sqrt{h_{1}^{2}h_{2}^{2}}}/{N_{c}}$:

$$\left( \begin{matrix} h_{1}^{2} & \sigma_{g} \\ \sigma_{g} & h_{2}^{2} \end{matrix} \right)$$

## Sample size inference for linear regression

For some studies, the effective sample size per SNP is not available. As demonstrated below, sample size can be approximated from the variance of the regression coefficient ($var\left( {\hat{\boldsymbol{\beta}}}_{\boldsymbol{G}} \right)$) and the variance of the variant tested ($\sigma_{G}^{2}$). Let’s consider a vector of predictor $\mathbf{X=(1,G,U)}$ of length$n$, where $\mathbf{1}$ is a vector of 1, $\mathbf{G}$ is a genetic variant and $\mathbf{U}$ is a variable that captures the aggregated effect of all covariates. When testing the association between $\mathbf{X}$ and an outcome$\mathbf{Y}$, the variance of the least square estimates $\hat{\beta}=(\hat{\beta}_{0},\hat{\beta}_{G},\hat{\beta}_{U})$ equals:

$\boldsymbol{var}\left( \hat{\boldsymbol{\beta}} \right)\mathbf{=}\boldsymbol{\sigma}_{\boldsymbol{e}}^{\mathbf{2}}\left( \mathbf{X}^{\boldsymbol{t}}\mathbf{X} \right)^{\mathbf{-1}}\mathbf{=}\boldsymbol{\sigma}_{\boldsymbol{e}}^{\mathbf{2}}\left( \begin{matrix} \boldsymbol{1}^{\boldsymbol{t}}\boldsymbol{1} & \boldsymbol{G}^{\boldsymbol{t}}\boldsymbol{1} & \boldsymbol{U}^{\boldsymbol{t}}\mathbf{1} \\ \boldsymbol{G}^{\boldsymbol{t}}\boldsymbol{1} & \boldsymbol{G}^{\boldsymbol{t}}\boldsymbol{G} & \boldsymbol{G}^{\boldsymbol{t}}\boldsymbol{U} \\ \boldsymbol{U}^{\boldsymbol{t}}\boldsymbol{1} & \boldsymbol{G}^{\boldsymbol{t}}\boldsymbol{U} & \boldsymbol{U}^{\boldsymbol{t}}\boldsymbol{U} \end{matrix} \right)^{\mathbf{-1}}$

For large sample size and assuming independence between predictors, the covariance matrix simplified to:

$$\boldsymbol{var}\left( \hat{\boldsymbol{\beta}} \right)\boldsymbol{\approx}\frac{\boldsymbol{\sigma}_{\boldsymbol{e}}^{\mathbf{2}}}{\boldsymbol{n}}\left( \begin{matrix} \mathbf{1} & \boldsymbol{\mu}_{\boldsymbol{G}} & \boldsymbol{\mu}_{\boldsymbol{U}} \\ \boldsymbol{\mu}_{\boldsymbol{G}} & \left( \boldsymbol{\mu}_{\boldsymbol{G}}^{\mathbf{2}}\mathbf{+}\boldsymbol{\sigma}_{\boldsymbol{G}}^{\mathbf{2}} \right) & \left( \boldsymbol{\mu}_{\boldsymbol{G}}\boldsymbol{\mu}_{\boldsymbol{U}} \right) \\ \boldsymbol{\mu}_{\boldsymbol{U}} & \left( \boldsymbol{\mu}_{\boldsymbol{G}}\boldsymbol{\mu}_{\boldsymbol{U}} \right) & \left( \boldsymbol{\mu}_{\boldsymbol{U}}^{\mathbf{2}}\mathbf{+}\boldsymbol{\sigma}_{\boldsymbol{U}}^{\mathbf{2}} \right) \end{matrix} \right)^{\mathbf{-1}}$$

It follows that:

$$\boldsymbol{var}\left( {\hat{\boldsymbol{\beta}}}_{\boldsymbol{G}} \right)\boldsymbol{\approx}\frac{\boldsymbol{\sigma}_{\boldsymbol{e}}^{\mathbf{2}}}{\boldsymbol{n}}\frac{\boldsymbol{det}\left( \begin{matrix} \mathbf{1} & \boldsymbol{\mu}_{\boldsymbol{U}} \\ \boldsymbol{\mu}_{\boldsymbol{U}} & \left( \boldsymbol{\mu}_{\boldsymbol{G}}^{\mathbf{2}}\mathbf{+}\boldsymbol{\sigma}_{\boldsymbol{U}}^{\mathbf{2}} \right) \end{matrix} \right)}{\boldsymbol{det}\left( \begin{matrix} \mathbf{1} & \boldsymbol{\mu}_{\boldsymbol{G}} & \boldsymbol{\mu}_{\boldsymbol{U}} \\ \boldsymbol{\mu}_{\boldsymbol{G}} & \left( \boldsymbol{\mu}_{\boldsymbol{G}}^{\mathbf{2}}\mathbf{+}\boldsymbol{\sigma}_{\boldsymbol{G}}^{\mathbf{2}} \right) & \left( \boldsymbol{\mu}_{\boldsymbol{G}}\boldsymbol{\mu}_{\boldsymbol{U}} \right) \\ \boldsymbol{\mu}_{\boldsymbol{U}} & \left( \boldsymbol{\mu}_{\boldsymbol{G}}\boldsymbol{\mu}_{\boldsymbol{U}} \right) & \left( \boldsymbol{\mu}_{\boldsymbol{U}}^{\mathbf{2}}\mathbf{+}\boldsymbol{\sigma}_{\boldsymbol{U}}^{\mathbf{2}} \right) \end{matrix} \right)}$$

$$\boldsymbol{\approx}\frac{\boldsymbol{\sigma}_{\boldsymbol{e}}^{\mathbf{2}}}{\boldsymbol{n}}\frac{\left( \boldsymbol{\mu}_{\boldsymbol{G}}^{\mathbf{2}}\mathbf{+}\boldsymbol{\sigma}_{\boldsymbol{U}}^{\mathbf{2}} \right)\mathbf{-}\boldsymbol{\mu}_{\boldsymbol{U}}^{\mathbf{2}}}{\left( \boldsymbol{\mu}_{\boldsymbol{G}}^{\mathbf{2}}\mathbf{+}\boldsymbol{\sigma}_{\boldsymbol{G}}^{\mathbf{2}} \right)\left( \boldsymbol{\mu}_{\boldsymbol{U}}^{\mathbf{2}}\mathbf{+}\boldsymbol{\sigma}_{\boldsymbol{U}}^{\mathbf{2}} \right)\mathbf{-}\left( \boldsymbol{\mu}_{\boldsymbol{G}}\boldsymbol{\mu}_{\boldsymbol{U}} \right)^{\mathbf{2}}\mathbf{-}\boldsymbol{\mu}_{\boldsymbol{G}}^{\mathbf{2}}\left( \boldsymbol{\mu}_{\boldsymbol{U}}^{\mathbf{2}}\mathbf{+}\boldsymbol{\sigma}_{\boldsymbol{U}}^{\mathbf{2}} \right)\mathbf{+ 2}\boldsymbol{\mu}_{\boldsymbol{G}}\boldsymbol{\mu}_{\boldsymbol{U}}\left( \boldsymbol{\mu}_{\boldsymbol{G}}\boldsymbol{\mu}_{\boldsymbol{U}} \right)\mathbf{-}\boldsymbol{\mu}_{\boldsymbol{U}}^{\mathbf{2}}\left( \boldsymbol{\mu}_{\boldsymbol{G}}^{\mathbf{2}}\mathbf{+}\boldsymbol{\sigma}_{\boldsymbol{G}}^{\mathbf{2}} \right)}$$

$$\boldsymbol{\approx}\frac{\boldsymbol{\sigma}_{\boldsymbol{e}}^{\boldsymbol{2}}}{\boldsymbol{n}}\frac{\boldsymbol{\sigma}_{\boldsymbol{U}}^{\boldsymbol{2}}}{\boldsymbol{\sigma}_{\boldsymbol{G}}^{\boldsymbol{2}}\boldsymbol{\sigma}_{\boldsymbol{U}}^{\boldsymbol{2}}}$$

$$\boldsymbol{\approx}\frac{\boldsymbol{\sigma}_{\boldsymbol{e}}^{\boldsymbol{2}}}{\boldsymbol{n}\boldsymbol{\sigma}_{\boldsymbol{G}}^{\boldsymbol{2}}}$$

The sample size $n$ can therefore be approximated by $\frac{\sigma_{e}^{2}}{var\left( {\hat{\boldsymbol{\beta}}}_{\boldsymbol{G}} \right) \sigma_{G}^{2}}$. As showed in **S11 Fig**, this approximation shows reasonable accuracy as long as the provided variant frequency is within ±0.01 of the true frequency. For larger differences, the estimated $n$ can vary substantially for relative rare variants (i.e. MAF<5%).

## Sample size inference for logistic regression

As described for linear regression in the section above, we aim at approximating the sample size per SNP. However, conversely to linear regression, there is no close form solution for the parameter of logistic regression. Nevertheless, a relation exists between maximum likelihood estimation using Fisher scoring and weighted least squares estimation[16]. Indeed, maximum likelihood equation for the ($t+1$)^th^ iteration has the form: $\boldsymbol{\beta}^{(t+1)}=\left( \mathbf{X}^{'}\mathbf{W}^{(t)}\mathbf{X} \right)^{-1}\mathbf{X}^{'}\mathbf{W}^{(t)}\mathbf{z}^{(t)}$, where $\mathbf{z}^{(t)}$ is the linearized form of the logit link function for the sample data. Using this formulation, the standard error estimate equals $\hat{\sigma}_{\beta}^{2}\approx1/\left( np(1-p)\sigma_{G}^{2} \right)$, where $p$ is the in-sample proportion of cases. Hence, similarly to the linear regression case, we propose using the term $1/\left( \hat{\sigma}_{\beta_{G}}^{2}\sigma_{G}^{2} \right)$ as a proxy for $W=np(1-p)$, which is proportional to sample size. Consider $w_{F}=n_{F}p_{F}(1-p_{F})$, the parameter $W$ obtained for the full sample, where $n=n_{F}$ and $p=p_{F}$. In theory, $w^{*}$, a given occurrence of $W$, can equals $w_{F}$, even though $n\neq n_{F}$ and $p\neq p_{F}$. However, assuming the number of cases and controls can only decrease, one can show that $w^{*}\leq w_{F}$, and therefore $W$ remains effective as a tool to filter SNP which statistics has been derived with missing individuals. Indeed, $w_{F}$ can be written as a function of $n_{ca}$ and $n_{co}$, the number of cases and controls in the full sample:

$$\boldsymbol{w}_{\boldsymbol{F}}\mathbf{=}\boldsymbol{np}\left( \mathbf{1-}\boldsymbol{p} \right)\mathbf{=}\left( \boldsymbol{n}_{\boldsymbol{ca}}\mathbf{+}\boldsymbol{n}_{\boldsymbol{co}} \right)\frac{\boldsymbol{n}_{\boldsymbol{ca}}}{\boldsymbol{n}_{\boldsymbol{ca}}\mathbf{+}\boldsymbol{n}_{\boldsymbol{co}}}\frac{\boldsymbol{n}_{\boldsymbol{co}}}{\boldsymbol{n}_{\boldsymbol{ca}}\mathbf{+}\boldsymbol{n}_{\boldsymbol{co}}}\mathbf{=}\frac{\boldsymbol{n}_{\boldsymbol{ca}}\boldsymbol{n}_{\boldsymbol{co}}}{\boldsymbol{n}_{\boldsymbol{ca}}\mathbf{+}\boldsymbol{n}_{\boldsymbol{co}}}$$

In comparison, $w^{*}$ is derived in situations where the number of cases or control differ using $n_{ca}^{*}=n_{ca}\times c_{1}$ and $n_{co}^{*}=n_{co}\times c_{2}$, where $c_{1}$ and $c_{2}$ are strictly positives:

$$\boldsymbol{w}^{\mathbf{*}}\mathbf{=}\frac{\boldsymbol{n}_{\boldsymbol{ca}}\boldsymbol{n}_{\boldsymbol{co}}\boldsymbol{c}_{\mathbf{1}}\boldsymbol{c}_{\mathbf{2}}}{\boldsymbol{n}_{\boldsymbol{ca}}\boldsymbol{c}_{\mathbf{1}}\mathbf{+}\boldsymbol{n}_{\boldsymbol{co}}\boldsymbol{c}_{\mathbf{2}}}\mathbf{=}\frac{\boldsymbol{n}_{\boldsymbol{ca}}\boldsymbol{n}_{\boldsymbol{co}}}{\frac{\boldsymbol{n}_{\boldsymbol{ca}}}{\boldsymbol{c}_{\mathbf{2}}}\mathbf{+}\frac{\boldsymbol{n}_{\boldsymbol{co}}}{\boldsymbol{c}_{\mathbf{1}}}}\mathbf{=}\boldsymbol{W}\frac{\boldsymbol{n}_{\boldsymbol{ca}}\mathbf{+}\boldsymbol{n}_{\boldsymbol{co}}}{\frac{\boldsymbol{n}_{\boldsymbol{ca}}}{\boldsymbol{c}_{\mathbf{2}}}\mathbf{+}\frac{\boldsymbol{n}_{\boldsymbol{co}}}{\boldsymbol{c}_{\mathbf{1}}}}$$

It follows that if $c_{1}$ and $c_{2}$ are both strictly smaller than 1, $w^{*}<w_{F}$.

As showed in **S12 Fig**, this indicator provides a reasonable proxy proportional to the true sample size.

## Imputation of missing z-score statistics

The number of SNPs reported in GWAS summary statistics commonly differs between studies. All proposed multivariate approaches require complete data, so that jointly analyzing multiple GWAS might result in the removal of large number of variants. In order to increase the number of SNPs in cross-trait analyses, we performed z-scores imputations at missing variants within each single GWAS using high accuracy python package (RAISS) we recently developed[17]. RAISS improved on pre-existing solutions to reach a level of imputation accuracy suitable for multitrait analysis[18]. RAISS uses external information on linkage disequilibrium (LD) from a reference panel and z-scores for typed variants included in the study. Briefly, let $Z_{t}\mathcal{\sim N}\left( 0,A \right)$ denote the vector of z-scores for the typed variants, and $\Sigma_{t,t}$ denote the correlation matrix of typed genotypes. The vector of imputed z-scores $Z_{i}^{*}$ at non-typed variants can be computed as linear combination of typed z-scores $Z_{t}$ with weights $W=\Sigma_{i,t}\Sigma_{t,t}^{-1}$ where $\Sigma_{i,t}$ is the SNP correlation matrix including imputed and typed variants:

$$\boldsymbol{Z}_{\boldsymbol{i}}^{\mathbf{*}}\mathbf{=}\frac{\boldsymbol{W}}{\sqrt{\mathbf{W}\boldsymbol{A}\boldsymbol{W}^{\boldsymbol{t}}}}\boldsymbol{Z}_{\boldsymbol{t}}$$

The theoretical accuracy of the imputation is assessed using the variance of the conditional random variable $Z_{i|t}^{*}$ and defined as $r^{2}pred=1- \Sigma_{i|t}$, where $\Sigma_{i|t}=WAW^{t}$.

As demonstrated below, the imputed SNPs have the same expected covariance as the genotyped SNP. Consider two GWAS studies $y_{1}$ and $y_{2}$ and suppose (without loss of generality) that the sets of genotyped SNPs are identical in the two studies. Consider a SNP $j$ missing in the two studies. Hence, linear regression z-scores are computed as:

$$\boldsymbol{z}_{\mathbf{1}\boldsymbol{j}}^{\mathbf{*}}\mathbf{=}\frac{\boldsymbol{\Sigma}_{\boldsymbol{j}\mathbf{,}\boldsymbol{t}}\boldsymbol{\Sigma}_{\boldsymbol{t}\mathbf{,}\boldsymbol{t}}^{\mathbf{-1}}}{\sqrt{\boldsymbol{\Sigma}_{\boldsymbol{j}\mathbf{,}\boldsymbol{t}}\boldsymbol{\Sigma}_{\boldsymbol{t}\mathbf{,}\boldsymbol{t}}^{\mathbf{-1}}\boldsymbol{A}\boldsymbol{\Sigma}_{\boldsymbol{t}\mathbf{,}\boldsymbol{t}}^{\mathbf{-1}}\boldsymbol{\Sigma}_{\boldsymbol{j}\mathbf{,}\boldsymbol{t}}^{\boldsymbol{t}}}}\boldsymbol{Z}_{\boldsymbol{t}}$$

$$\boldsymbol{z}_{\mathbf{2}\boldsymbol{j}}^{\mathbf{*}}\mathbf{=}\frac{\boldsymbol{\Sigma}_{\boldsymbol{j}\mathbf{,}\boldsymbol{u}}\boldsymbol{\Sigma}_{\boldsymbol{u}\mathbf{,}\boldsymbol{u}}^{\mathbf{-1}}}{\sqrt{\boldsymbol{\Sigma}_{\boldsymbol{j}\mathbf{,}\boldsymbol{u}}\boldsymbol{\Sigma}_{\boldsymbol{u}\mathbf{,}\boldsymbol{u}}^{\mathbf{-1}}\boldsymbol{A}\boldsymbol{\Sigma}_{\boldsymbol{u}\mathbf{,}\boldsymbol{u}}^{\mathbf{-1}}\boldsymbol{\Sigma}_{\boldsymbol{j}\mathbf{,}\boldsymbol{u}}^{\boldsymbol{t}}}}\boldsymbol{Z}_{\boldsymbol{u}}$$

It follows that the covariance between the imputed z-score equals:

$$\mathbb{E}\left[ \boldsymbol{z}_{\mathbf{1}\boldsymbol{j}}^{\mathbf{*}}\boldsymbol{z}_{\mathbf{2}\boldsymbol{j}}^{\mathbf{*}} \right]\mathbb{=E}\left[ \frac{\boldsymbol{\Sigma}_{\boldsymbol{j}\mathbf{,}\boldsymbol{t}}\boldsymbol{\Sigma}_{\boldsymbol{t}\mathbf{,}\boldsymbol{t}}^{\mathbf{-1}}}{\boldsymbol{r}_{\mathbf{1,}\boldsymbol{pred}}}\boldsymbol{Z}_{\mathbf{1}\boldsymbol{t}}\left( \frac{\boldsymbol{\Sigma}_{\boldsymbol{j}\mathbf{,}\boldsymbol{u}}\boldsymbol{\Sigma}_{\boldsymbol{u}\mathbf{,}\boldsymbol{u}}^{\mathbf{-1}}}{\boldsymbol{r}_{\mathbf{2,}\boldsymbol{pred}}}\boldsymbol{Z}_{\mathbf{2}\boldsymbol{u}} \right)^{\boldsymbol{t}} \right]$$

$$\mathbf{=}\frac{\mathbf{1}}{\boldsymbol{r}_{\mathbf{1,}\boldsymbol{pred}}\boldsymbol{r}_{\mathbf{2,}\boldsymbol{pred}}}\mathbb{E}\left[ \boldsymbol{\Sigma}_{\boldsymbol{j}\mathbf{,}\boldsymbol{t}}\boldsymbol{\Sigma}_{\boldsymbol{t}\mathbf{,}\boldsymbol{t}}^{\mathbf{-1}}\boldsymbol{Z}_{\mathbf{1}\boldsymbol{t}}\left( \boldsymbol{\Sigma}_{\boldsymbol{j}\mathbf{,}\boldsymbol{u}}\boldsymbol{\Sigma}_{\boldsymbol{u}\mathbf{,}\boldsymbol{u}}^{\mathbf{-1}}\boldsymbol{Z}_{\mathbf{2}\boldsymbol{u}} \right)^{\boldsymbol{t}} \right]$$

$$\mathbf{=}\frac{\mathbf{1}}{\boldsymbol{r}_{\mathbf{1,}\boldsymbol{pred}}\boldsymbol{r}_{\mathbf{2,}\boldsymbol{pred}}}\mathbb{E}\left[ \boldsymbol{\Sigma}_{\boldsymbol{j}\mathbf{,}\boldsymbol{t}}\boldsymbol{\Sigma}_{\boldsymbol{t}\mathbf{,}\boldsymbol{t}}^{\mathbf{-1}}\boldsymbol{Z}_{\mathbf{1}\boldsymbol{t}}\boldsymbol{Z}_{\mathbf{2}\boldsymbol{u}}^{\boldsymbol{t}}\boldsymbol{\Sigma}_{\boldsymbol{u}\mathbf{,}\boldsymbol{u}}^{\mathbf{-1}}\boldsymbol{\Sigma}_{\boldsymbol{j}\mathbf{,}\boldsymbol{u}}^{\boldsymbol{t}} \right]$$

$$\mathbf{=}\frac{\mathbf{1}}{\boldsymbol{r}_{\mathbf{1,}\boldsymbol{pred}}\boldsymbol{r}_{\mathbf{2,}\boldsymbol{pred}}}\boldsymbol{\Sigma}_{\boldsymbol{j}\mathbf{,}\boldsymbol{t}}\boldsymbol{\Sigma}_{\boldsymbol{t}\mathbf{,}\boldsymbol{t}}^{\mathbf{-1}}\mathbb{E}\left[ \boldsymbol{Z}_{\mathbf{1}\boldsymbol{t}}\boldsymbol{Z}_{\mathbf{2}\boldsymbol{u}}^{\boldsymbol{t}} \right]\boldsymbol{\Sigma}_{\boldsymbol{u}\mathbf{,}\boldsymbol{u}}^{\mathbf{-1}}\boldsymbol{\Sigma}_{\boldsymbol{j}\mathbf{,}\boldsymbol{u}}^{\boldsymbol{t}}$$

However, we have $\mathbb{E}\left[ Z_{1t}Z_{2u}^{t} \right]\mathbb{=E}\left[ z_{1j}z_{2j} \right]\Sigma_{t,u}$, where $\Sigma_{t,u}$ is the covariance matrix between SNPs $t$ and $u$. It follows that:

$$\mathbb{E}\left[ \boldsymbol{z}_{\mathbf{1}\boldsymbol{j}}^{\mathbf{*}}\boldsymbol{z}_{\mathbf{2}\boldsymbol{j}}^{\mathbf{*}} \right]\mathbf{=}\frac{\mathbf{1}}{\boldsymbol{r}_{\mathbf{1,}\boldsymbol{pred}}\boldsymbol{r}_{\mathbf{2,}\boldsymbol{pred}}}\boldsymbol{\Sigma}_{\boldsymbol{j}\mathbf{,}\boldsymbol{t}}\boldsymbol{\Sigma}_{\boldsymbol{t}\mathbf{,}\boldsymbol{t}}^{\mathbf{-1}}\left[ \mathbb{E}\left[ \boldsymbol{z}_{\mathbf{1}\boldsymbol{j}}\boldsymbol{z}_{\mathbf{2}\boldsymbol{j}} \right]\boldsymbol{\Sigma}_{\boldsymbol{t}\mathbf{,}\boldsymbol{u}} \right]\boldsymbol{\Sigma}_{\boldsymbol{u}\mathbf{,}\boldsymbol{u}}^{\mathbf{-1}}\boldsymbol{\Sigma}_{\boldsymbol{j}\mathbf{,}\boldsymbol{u}}^{\boldsymbol{t}}$$

$$\mathbf{=}\frac{\mathbb{E}\left[ \boldsymbol{z}_{\mathbf{1}\boldsymbol{j}}\boldsymbol{z}_{\mathbf{2}\boldsymbol{j}} \right]}{\boldsymbol{r}_{\mathbf{1,}\boldsymbol{pred}}\boldsymbol{r}_{\mathbf{2,}\boldsymbol{pred}}}\boldsymbol{\Sigma}_{\boldsymbol{j}\mathbf{,}\boldsymbol{t}}\boldsymbol{\Sigma}_{\boldsymbol{t}\mathbf{,}\boldsymbol{t}}^{\mathbf{-1}}\boldsymbol{\Sigma}_{\boldsymbol{t}\mathbf{,}\boldsymbol{u}}\boldsymbol{\Sigma}_{\boldsymbol{u}\mathbf{,}\boldsymbol{u}}^{\mathbf{-1}}\boldsymbol{\Sigma}_{\boldsymbol{j}\mathbf{,}\boldsymbol{u}}^{\boldsymbol{t}}$$

$$\mathbf{=}\frac{\mathbb{E}\left[ \boldsymbol{z}_{\mathbf{1}\boldsymbol{j}}\boldsymbol{z}_{\mathbf{2}\boldsymbol{j}} \right]}{\boldsymbol{r}_{\mathbf{1,}\boldsymbol{pred}}\boldsymbol{r}_{\mathbf{2,}\boldsymbol{pred}}}\boldsymbol{\Sigma}_{\boldsymbol{j}\mathbf{,}\boldsymbol{t}}\boldsymbol{\Sigma}_{\boldsymbol{t}\mathbf{,}\boldsymbol{u}}^{\mathbf{-1}}\boldsymbol{\Sigma}_{\boldsymbol{j}\mathbf{,}\boldsymbol{u}}^{\boldsymbol{t}}$$

$$\mathbb{=E}\left[ \boldsymbol{z}_{\mathbf{1}\boldsymbol{j}}\boldsymbol{z}_{\mathbf{2}\boldsymbol{j}} \right]$$

As a naïve sanity check we plotted in **S13 Fig** the QQplot for multiple traits before and after imputation and did not observed any inflation.

## Unbiased genetic covariance estimator by clusters

We defined the genetic covariance by clusters for trait 1 and trait 2 as:

$\rho_{g, cluster=}\frac{\beta_{1}^{t}\beta_{2}}{M}$

Where $\beta_{1}$ and $\beta_{2}$ are the the vector of genetic effects of the M SNPs contained in the cluster. However to estimate properly this quantity from the observed $\hat{\beta}$, we need to take into account the bias introduce by sample overlap:

$\frac{\mathbb{E}\left( {\hat{\beta}_{1}}^{T}\hat{\beta}_{2} \right)}{M}$= $\frac{1}{M} \sum_{j=1}^{M} \mathbb{E}\left( \frac{{x_{j,1}}^{T}y_{1}}{{x_{j,1}}^{T}x_{j,1}} \times\frac{{x_{j,2}}^{T}y_{1}}{{x_{j,2}}^{T}x_{j,2}} \right)= \frac{1}{M} \sum_{j=1}^{M} \mathbb{E}\left( \frac{{x_{j,1}}^{T}\left( \beta_{1}x_{j,1}+\varepsilon_{1} \right)}{n_{1}} \times\frac{{x_{j,2}}^{T}\left( \beta_{2}x_{j,2}+ \varepsilon_{2} \right)}{n_{2}} \right)$

$$= \frac{1}{M} \sum_{j=1}^{M} \mathbb{E}\left( \frac{{x_{j,1}}^{T}{x_{j,1}\beta}_{j,1}}{{x_{j,1}}^{T}x_{j,1}} \times\frac{{x_{j,2}}^{T}x_{j,2}\beta_{j,2}}{{x_{j,2}}^{T}x_{j,2}}+ \frac{{x_{j,1}}^{T}\varepsilon_{1}}{n_{1}} \times\frac{{\varepsilon_{2}}^{T}x_{j,2}}{n_{2}} \right)$$

$$= \frac{1}{M} \sum_{j=1}^{M} {\beta_{j,1}\beta}_{j,2}\mathbb{+ E}\left( {\varepsilon_{1}}^{T}\left( \begin{matrix} I_{n_{s}} & O_{n_{s}\times(n_{2}-n_{s})} \\ O_{n_{s}\times(n_{1}-n_{s})} & O_{(n_{1}-n_{s})\times(n_{2}-n_{s})} \end{matrix} \right)\varepsilon_{2} \right)$$

$$= \frac{1}{M} \sum_{j=1}^{M} {\beta_{j,1}\beta}_{j,2}+\frac{n_{s}\rho}{n_{1}n_{2}}$$

Hence an unbiased estimator of the mean genetic covariance is :

$\mathbb{E}\left( {\beta_{1}}^{T}\beta_{2} \right)= \frac{\mathbb{E}\left( {\hat{\beta}_{1}}^{T}\hat{\beta}_{2} \right)}{M}$ - $\frac{n_{s}\rho}{n_{1}n_{2}}$

## Tissue enrichment analysis

We tested enrichment for SNPs belonging to regions of open chromatin (more likely to contain expressed genes[19, 20][) in specific tissues in three cases: i) when comparing results across GWAS sets, ii) when comparing univariate results,](#_ENREF_28) and iii) when comparing results across clusters. For all analyses we used functional annotations on 127 Roadmap tissues and cell lines defined by integrating activating histone marks (H3K4me1, H3K4me3, H3K9ac, and H3K27ac) with a latent Dirichlet allocation model as implemented in FUN-LDA[21]. Let $R_{1}$,…, $R_{127}$ be the 127 Roadmap tissues. We are interested in identifying the Roadmap tissue $R_{j}$ with the highest enrichment in significant SNPs (*p*-value < $5\times{10}^{-8}$) from a given set of results $G$. We first define:

$$\boldsymbol{p}_{\boldsymbol{G}\boldsymbol{|}\boldsymbol{R}_{\boldsymbol{j}}}\boldsymbol{=}\frac{\boldsymbol{\#significant SNPs in functional component}\mathbf{R}_{\mathbf{j}}}{\boldsymbol{\#SNPs in functional component}\mathbf{R}_{\mathbf{j}}}$$

where the functional component is defined by FUN-LDA score > 0.9 (probability to be functional >0.9) for $R_{j}$. To test whether there is an enrichment in the functional component of Roadmap tissue $R_{j}$, we compare $p_{G|R_{j}}$ against:

$$\boldsymbol{p}_{\boldsymbol{G}\boldsymbol{|}\boldsymbol{R}_{\boldsymbol{-j}}}\boldsymbol{=}\frac{\boldsymbol{\#significant SNPs in functional components excluding}\mathbf{R}_{\mathbf{j}}}{\boldsymbol{\#SNPs in functional components excluding}\mathbf{R}_{\mathbf{j}}}$$

where functional components excluding $R_{j}$ is defined by FUN-LDA score < 0.1 (probability to be functional <0.1) for $R_{j}$ but >0.9 for any other tissues/cell types. The null hypothesis of our test is $H_{0}: p_{G|R_{j}}= p_{G|R_{-j}}$ *versus*$H_{1}: p_{G|R_{j}}> p_{G|R_{-j}}$. For each analysis, we applied a two-sample proportion test for each Roadmap tissue $R_{j}$ to derive the p-value, and reported the enrichment ratio ${p_{G|R_{j}}}/{p_{G|R_{-j}}}$. To account for the total number of tests, we further applied an FDR adjustment. When comparing results from GWAS sets we adjusted for 23 tests $\times$ 127 tissues/cell types. When comparing results from univariate analyses, we adjusted for 36 phenotypes $\times$ 127 tissues/cell types. Finally, when comparing clusters, we adjusted for 41 clusters $\times$ 127 tissues/cell types.

Supplementary References

1. Cichonska A, Rousu J, Marttinen P, Kangas AJ, Soininen P, Lehtimaki T, et al. metaCCA: summary statistics-based multivariate meta-analysis of genome-wide association studies using canonical correlation analysis. Bioinformatics. 2016;32(13):1981-9. Epub 2016/05/07. doi: 10.1093/bioinformatics/btw052. PubMed PMID: 27153689; PubMed Central PMCID: PMCPMC4920109.

2. van der Sluis S, Posthuma D, Dolan CV. TATES: efficient multivariate genotype-phenotype analysis for genome-wide association studies. PLoS genetics. 2013;9(1):e1003235. Epub 2013/01/30. doi: 10.1371/journal.pgen.1003235. PubMed PMID: 23359524; PubMed Central PMCID: PMC3554627.

3. Qi G, Chatterjee N. Heritability informed power optimization (HIPO) leads to enhanced detection of genetic associations across multiple traits. PLoS Genet. 2018;14(10):e1007549. Epub 2018/10/06. doi: 10.1371/journal.pgen.1007549. PubMed PMID: 30289880.

4. Zhu X, Feng T, Tayo BO, Liang J, Young JH, Franceschini N, et al. Meta-analysis of correlated traits via summary statistics from GWASs with an application in hypertension. American journal of human genetics. 2015;96(1):21-36. Epub 2014/12/17. doi: 10.1016/j.ajhg.2014.11.011. PubMed PMID: 25500260; PubMed Central PMCID: PMC4289691.

5. Kim J, Bai Y, Pan W. An Adaptive Association Test for Multiple Phenotypes with GWAS Summary Statistics. Genet Epidemiol. 2015;39(8):651-63. Epub 2015/10/24. doi: 10.1002/gepi.21931. PubMed PMID: 26493956; PubMed Central PMCID: PMCPMC4715495.

6. Ray D, Boehnke M. Methods for meta-analysis of multiple traits using GWAS summary statistics. Genetic epidemiology. 2018;42(2):134-45. Epub 2017/12/12. doi: 10.1002/gepi.22105. PubMed PMID: 29226385; PubMed Central PMCID: PMCPMC5811402.

7. Zhu X, Feng T, Tayo BO, Liang J, Young JH, Franceschini N, et al. Meta-analysis of correlated traits via summary statistics from GWASs with an application in hypertension. American Journal of Human Genetics. 2015;96(1):21-36. doi: 10.1016/j.ajhg.2014.11.011.

8. Julienne H, Lechat P, Guillemot V, Lasry C, Yao C, Laville V, et al. JASS: Command Line and Web interface for the joint analysis of GWAS results. bioRxiv. 2019.

9. Mardia KV, Kent JT, Bibby JM. Multivariate analysis: Academic Press; 1979.

10. Bartlett MS. Further aspects of the theory of multiple regression. Mathematical Proceedings of the Cambridge Philosophical Society. 1938;34(1):33-40. Epub 2008/10/24. doi: 10.1017/s0305004100019897.

11. Sudlow C, Gallacher J, Allen N, Beral V, Burton P, Danesh J, et al. UK Biobank: An Open Access Resource for Identifying the Causes of a Wide Range of Complex Diseases of Middle and Old Age. PLoS Medicine. 2015;12(3):1-10. doi: 10.1371/journal.pmed.1001779.

12. Bycroft C, Freeman C, Petkova D, Band G, Elliott LT, Sharp K, et al. 2017. doi: 10.1101/166298.

13. Shi H, Kichaev G, Pasaniuc B. Contrasting the Genetic Architecture of 30 Complex Traits from Summary Association Data. Am J Hum Genet. 2016;99(1):139-53. Epub 2016/06/28. doi: 10.1016/j.ajhg.2016.05.013. PubMed PMID: 27346688; PubMed Central PMCID: PMCPMC5005444.

14. Aguet F, Ardlie KG, Cummings BB, Gelfand ET, Getz G, Hadley K, et al. Genetic effects on gene expression across human tissues. Nature. 2017;550(7675):204-13. doi: 10.1038/nature24277.

15. Bulik-Sullivan B, Finucane HK, Anttila V, Gusev A, Day FR, Loh PR, et al. An atlas of genetic correlations across human diseases and traits. Nat Genet. 2015. Epub 2015/09/29. doi: 10.1038/ng.3406. PubMed PMID: 26414676.

16. Agresti A. Generalized Linear Models. An Introduction to Categorical Data Analysis: John Wiley & Sons, Inc.; 2006. p. 65-98.

17. Julienne H, Shi H, Pasaniuc B, Aschard H. RAISS: Robust and Accurate imputation from Summary Statistics. Bioinformatics. 2019. Epub 2019/06/08. doi: 10.1093/bioinformatics/btz466. PubMed PMID: 31173064.

18. Pasaniuc B, Zaitlen N, Shi H, Bhatia G, Gusev A, Pickrell J, et al. Fast and accurate imputation of summary statistics enhances evidence of functional enrichment. Bioinformatics (Oxford, England). 2014;30(20):2906-14. doi: 10.1093/bioinformatics/btu416.

19. Julienne H, Zoufir A, Audit B, Arneodo A. Human genome replication proceeds through four chromatin states. PLoS Comput Biol. 2013;9(10):e1003233. Epub 2013/10/17. doi: 10.1371/journal.pcbi.1003233. PubMed PMID: 24130466; PubMed Central PMCID: PMCPMC3794905.

20. Natarajan A, Yardimci GG, Sheffield NC, Crawford GE, Ohler U. Predicting cell-type-specific gene expression from regions of open chromatin. Genome Res. 2012;22(9):1711-22. Epub 2012/09/08. doi: 10.1101/gr.135129.111. PubMed PMID: 22955983; PubMed Central PMCID: PMCPMC3431488.

21. Backenroth D, He Z, Kiryluk K, Boeva V, Pethukova L, Khurana E, et al. FUN-LDA: A Latent Dirichlet Allocation Model for Predicting Tissue-Specific Functional Effects of Noncoding Variation: Methods and Applications. American Journal of Human Genetics. 2018;102:920-42. doi: 10.1016/j.ajhg.2018.03.026.
