# Supplementary figures and images for: Multitrait GWAS to connect disease variants and biological mechanisms
Source: PLoS Genet. 2021 Aug 30;17(8):e1009713. doi: 10.1371/journal.pgen.1009713 (PMC8437297; doi:10.1371/journal.pgen.1009713)

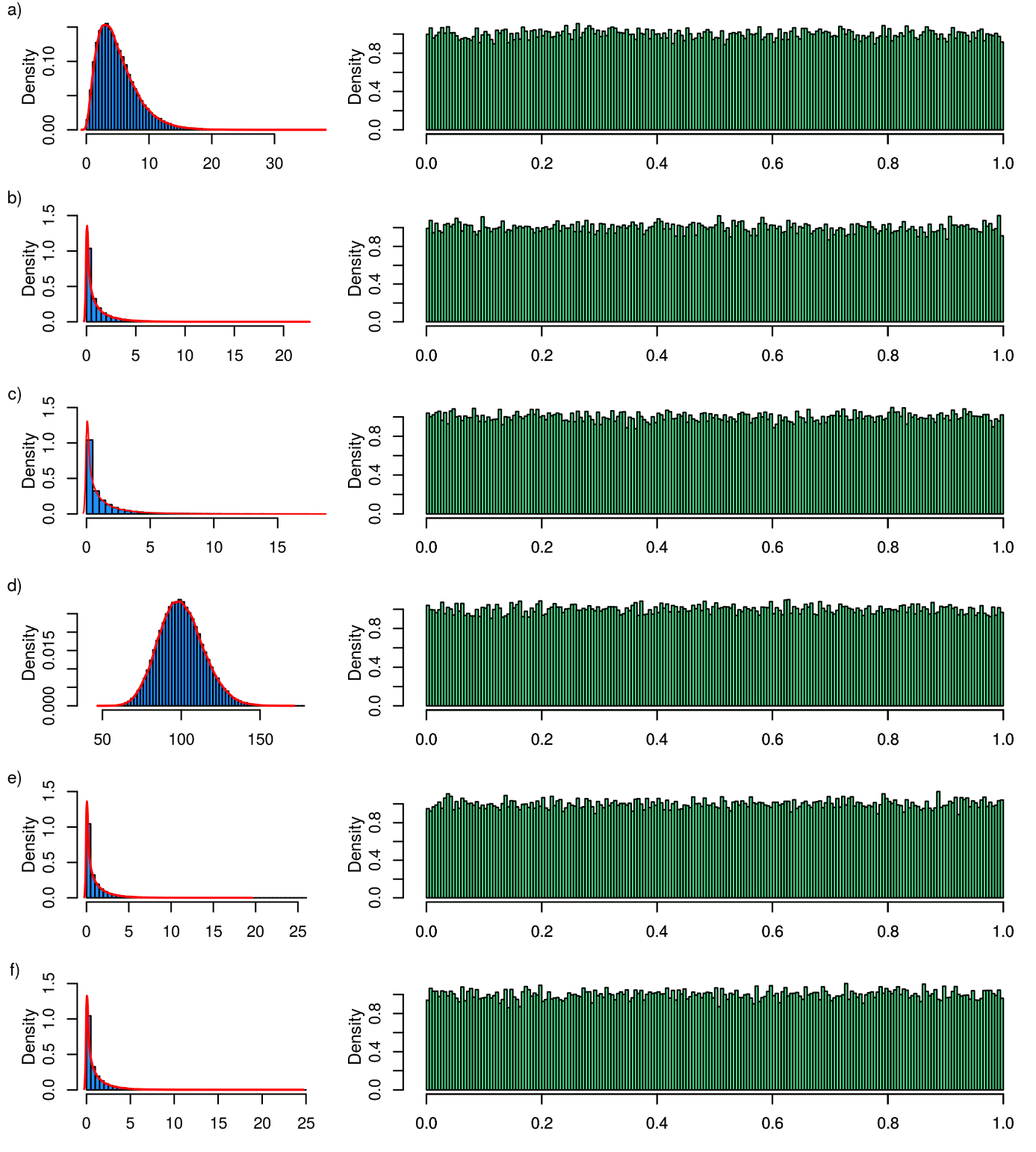

Supplement: S1 Fig — We simulated series of 10,000 replicates, each including z-score statistics for 5 (panels a-c) and 100 (panels d-f) phenotypes and 100K SNPs. For each replicate we applied the Omnibus test (panels a and d), the sumZ test using weights of 1 (panels c and f) and test sumZ test using weights equals to loading of the first principal component of the phenotypic correlation matrix. Left panels show the observed chi-square distribution in blue against the expected one in red. Right panels show the corresponding p-value histograms. (TIF) [file pgen.1009713.s002.tif]

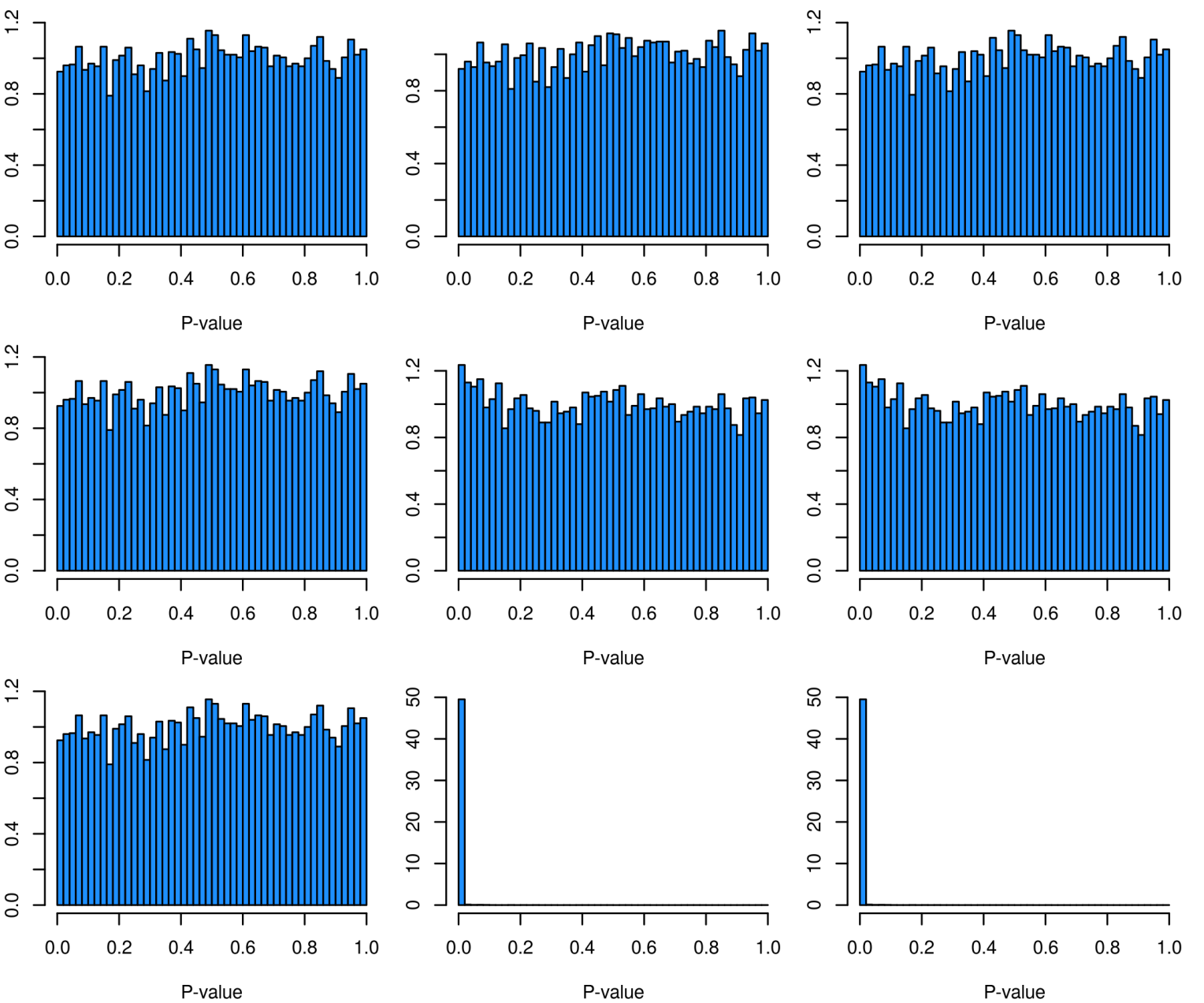

Supplement: S2 Fig — We simulated series of replicates, each including 20,000 individuals, 10,000 SNPs additively coded with frequencies ranging from 0.01 to 0.99, and 100 phenotypes. The phenotypes were drawn from a multivariate normal distribution with mean 0 and a variance-covariance Σr of rank 50. For each SNP, we computed the z-score of association with each phenotype using linear regressions and then derived p-values from the Omnibus test using three strategies to derive the inverse of Σr: i) considering only eigenvalues greater than a specified threshold ϵ (left column), ii) replacing eigenvalues below a threshold ϵ by ϵ (middle column), and iii) adding a small value ϵ to the diagonal terms of Σr (right column). For each strategy we considered three different ϵ values: 10−3(first line), 10−6(second line), and 10−9(last line). The plots show the histogram of p-value distribution. (TIF) [file pgen.1009713.s003.tif]

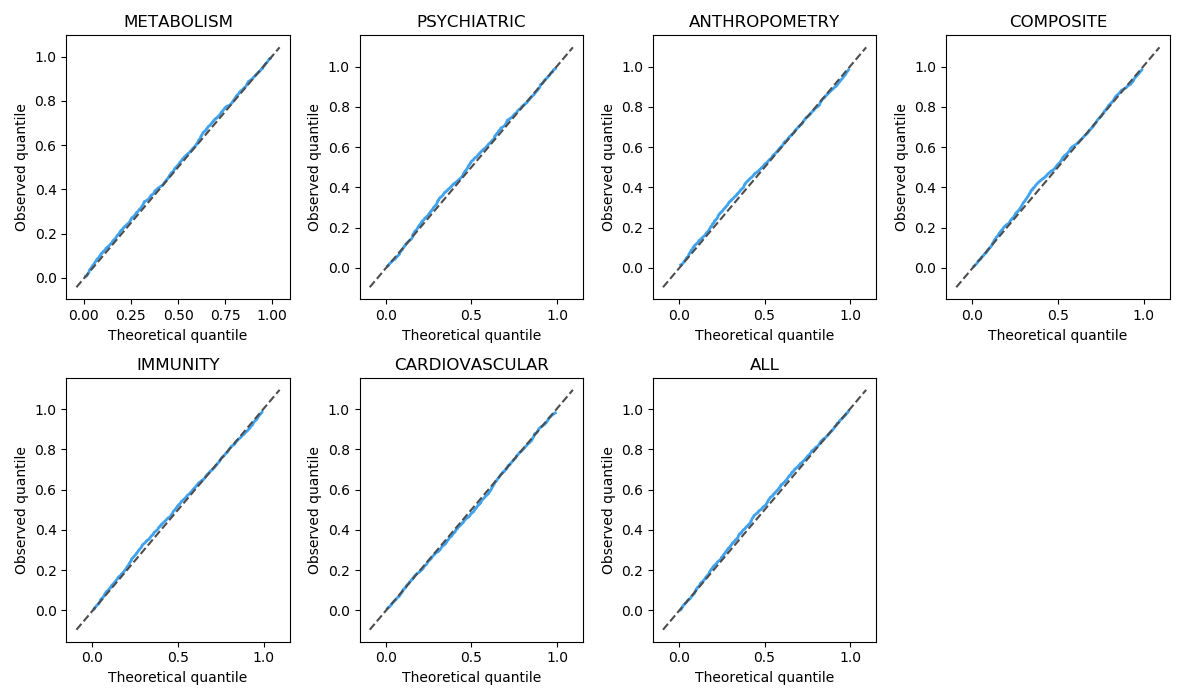

Supplement: S3 Fig — We simulated 1000 z-score vectors under the null hypothesis for each of the 7 GWAS set. The z-score vectors under the null hypothesis follow a multivariate Gaussian of null mean and of covariance given by the intercept of the LDSC regression. The ICA was applied on the 1000 simulated z-scores. For each component, the sumZ test using the component as weight vector. We then selected the component yielding the most association as final component. We computed the p-value for the 1000 point for the optimal component. The y-axis represents the observed p-value quantiles with respect to the theoretical p-value quantiles. (TIF) [file pgen.1009713.s004.tif]

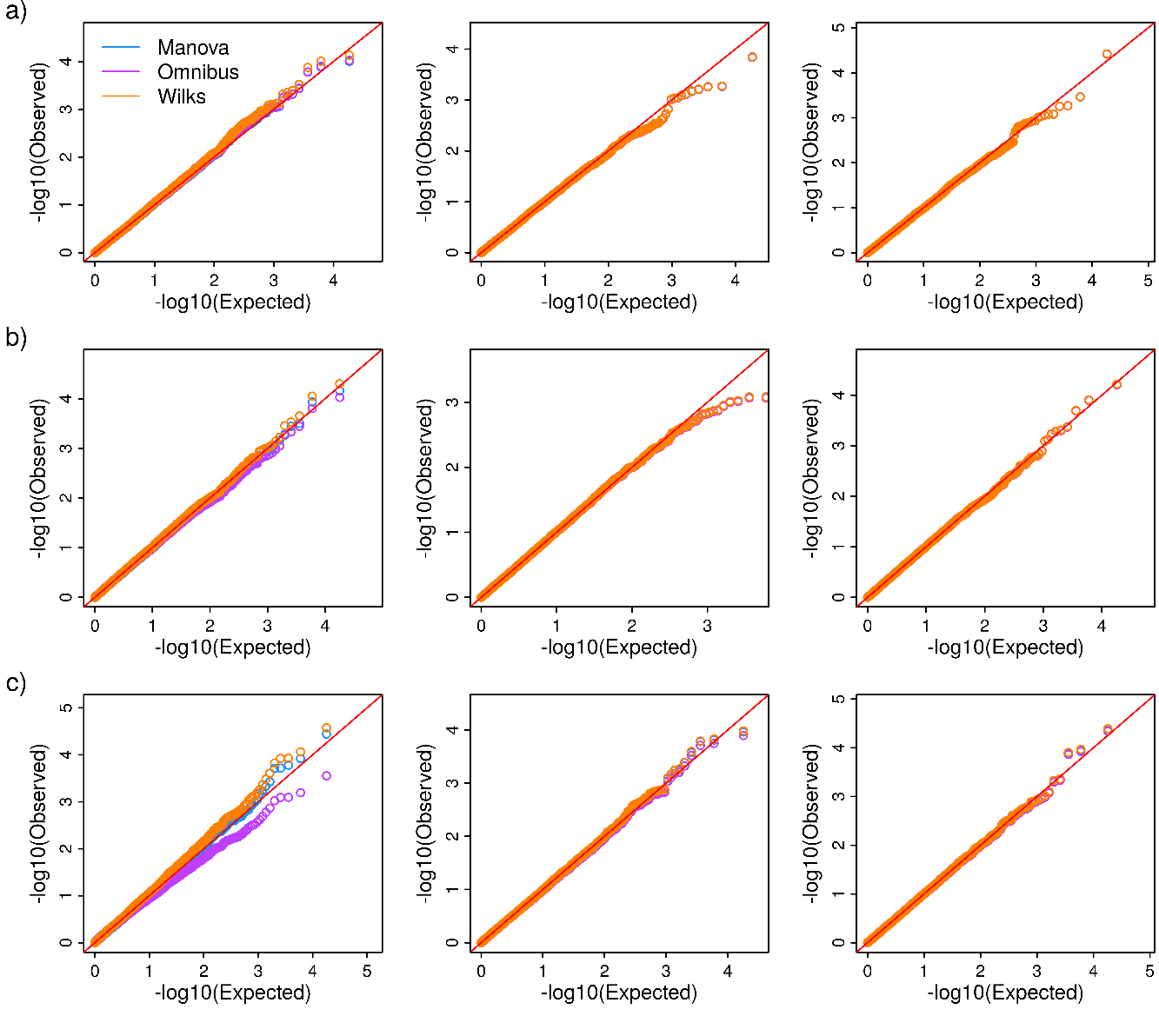

Supplement: S4 Fig — We simulated replicates of 10,000 predictors for 500 (left column), 5,000 (middle column) and 10,000 individuals (right column). Predictors were drawn from a binomial with n = 2 and probability varying in [0.01–0.99] to mimic genotype data, and further normalized to have mean 0 and variance 1. We then simulated a) 5, b) 20 and c) 100 correlated outcomes independent from the genotypes. To assess the impact of non-normal outcomes on the relationship between the summary-statistics based test and the individual-level data test, the outcomes were first drawn from a multivariate normal distribution with pairwise correlation ranging in [-0.7; 0.7] and then transform to non-normal distributions based on their quantiles, so that on average, 33% of them follow a uniform distribution, 33% a Laplace distribution, and 33% an exponential distribution. For each SNP, we first applied a Multivariate ANOVA (MANOVA, blue line). We then conducted association screenings for each single phenotype separately, and applied the proposed Omnibus test (purple line) and the Wilk’s approximation (orange line) on the resulting summary statistics. The plots show the observed -log10(p-value) of each test against the expected value under the null. (TIF) [file pgen.1009713.s005.tif]

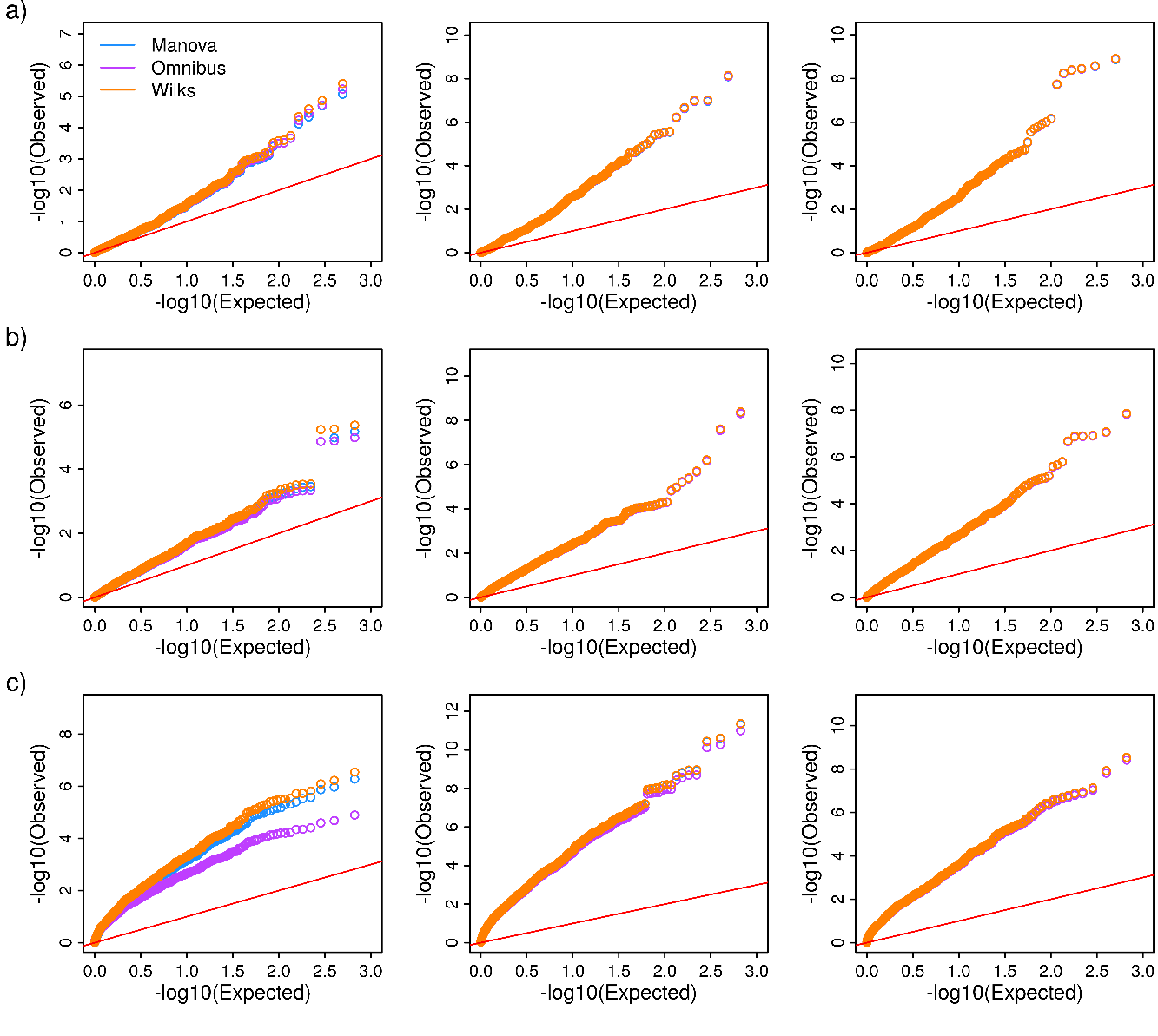

Supplement: S5 Fig — We simulated replicates of 10,000 predictors for 500 (left column), 5,000 (middle column) and 10,000 individuals (right column). Predictors were drawn from a binomial with n = 2 and probability varying in [0.01–0.99] to mimic genotype data, and further normalized to have mean 0 and variance 1. We then simulated a) 5, b) 20 and c) 100 correlated outcomes. We assumed that 10% of the predictors were causal, each causal predictor being randomly chosen and associated with up to 50% outcomes, randomly chosen with equal probability. Effect sizes for each causal predictor were drawn from a normal distribution with mean 0 and variance 0.0025 (i.e. the total phenotypic variance explained by the predictors). To assess the impact of non-normal outcomes on the relationship between the summary-statistics based test and the individual-level data test, the outcomes were first drawn from a multivariate normal distribution with pairwise correlation ranging in [-0.7; 0.7] and then transform to non-normal distributions based on their quantiles, so that on average, 33% of them follow a uniform distribution, 33% a Laplace distribution, and 33% an exponential distribution. For each SNP, we first applied a Multivariate ANOVA (MANOVA, blue line). We then conducted association screenings for each single phenotype separately, and applied the proposed Omnibus test (purple line) and the Wilk’s approximation (orange line) on the resulting summary statistics. The plots show the observed -log10(p-value) of each test against the expected value under the null. (TIF) [file pgen.1009713.s006.tif]

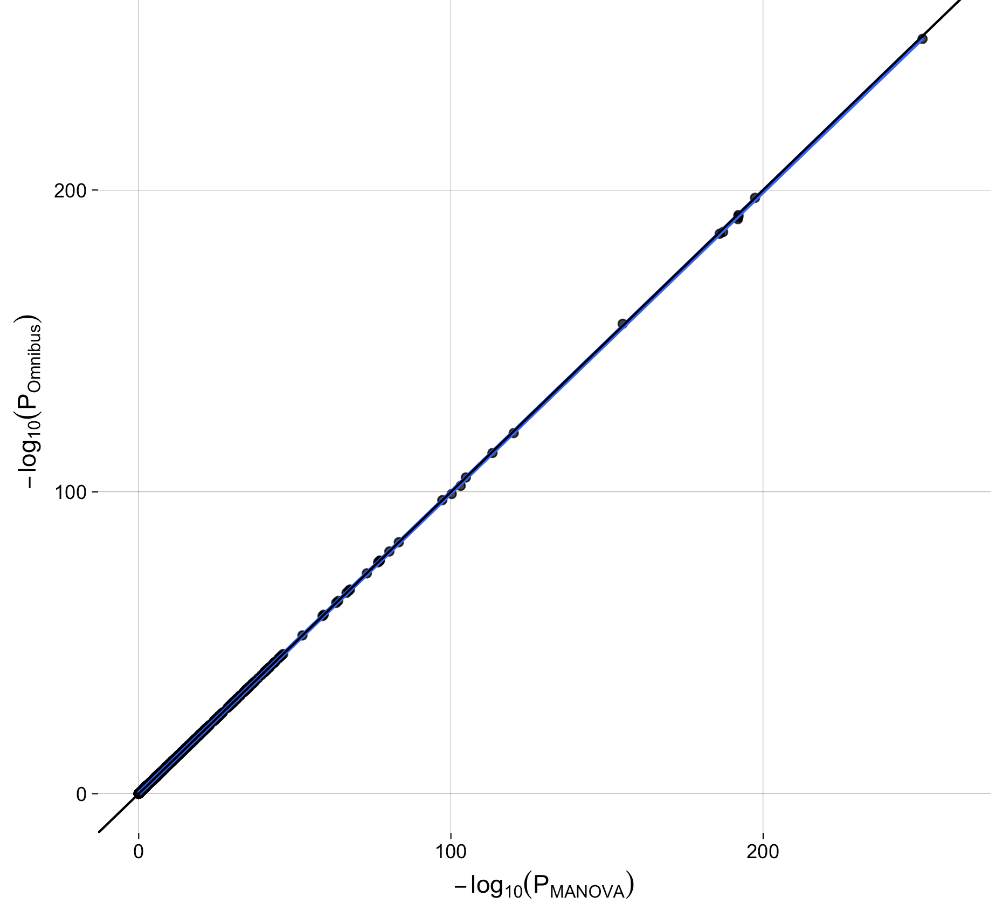

Supplement: S6 Fig — Multi-trait GWAS was performed in real data from the UK Biobank using five traits, 336,347 unrelated individuals, and 14,718 genotyped SNPs on Chromosome 20. We used two methods: MANOVA as implemented in PLINK, and the Omnibus test from JASS. The plot shows the -log10(p-values) of the Omnibus approach as a function of the -log10(p-values) derived with the MANOVA. (TIF) [file pgen.1009713.s007.tif]

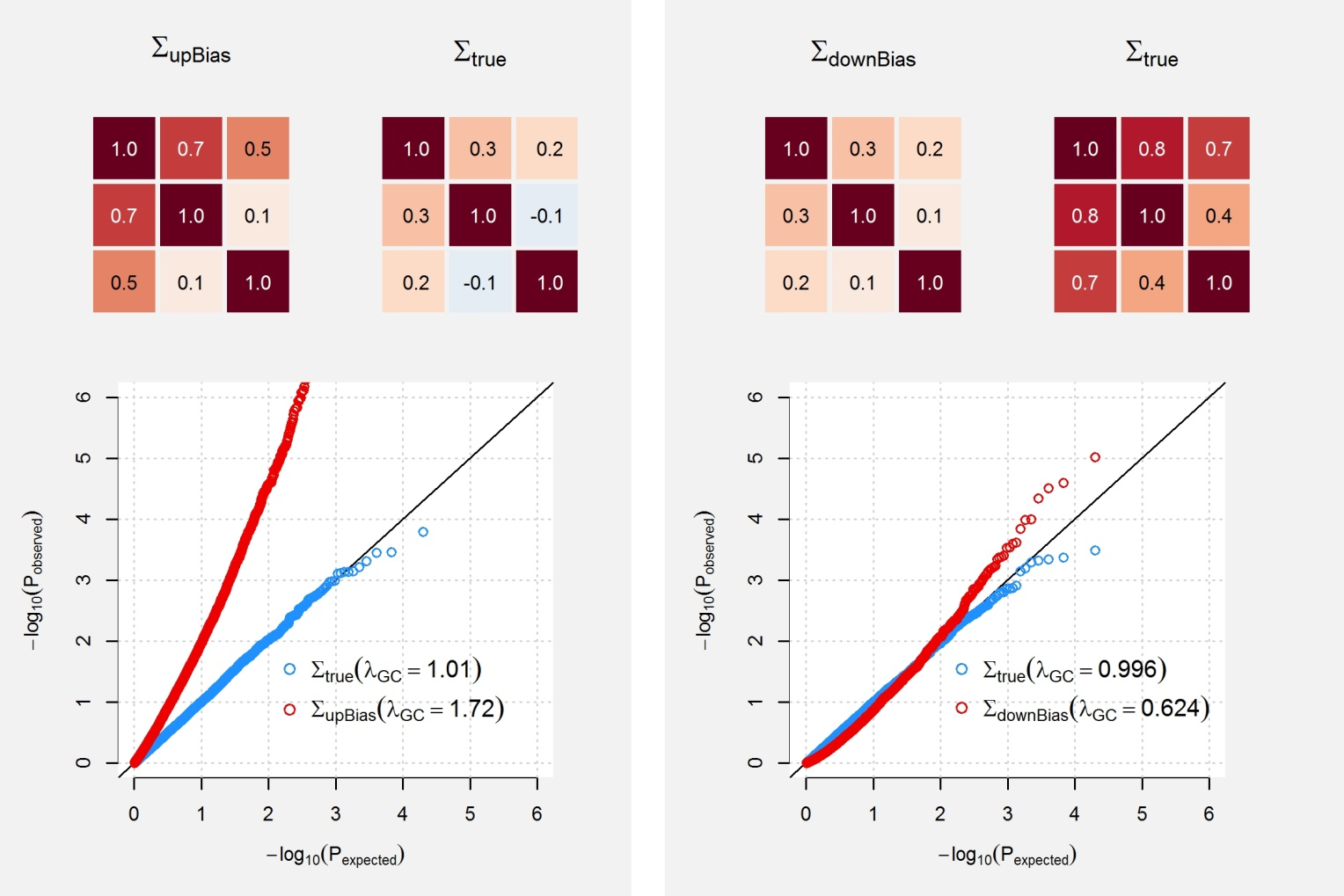

Supplement: S7 Fig — We simulated three correlated vectors of z-scores for 10,000 SNPs. We derived the multivariate test for each SNP using either the true covariance matrix (Σtrue), an upward-bias covariance matrix (ΣupBias, left panels) or a downward-bias covariance matrix (ΣdownBias, right panels). For both scenarios we derived the Omnibus multivariate test using either the true or biased covariance matrix. Invalidity of the test based on the biased covariance matrix is illustrated in the resulting QQplots and genomic inflation factor λGC (bottom panels). (TIF) [file pgen.1009713.s008.tif]

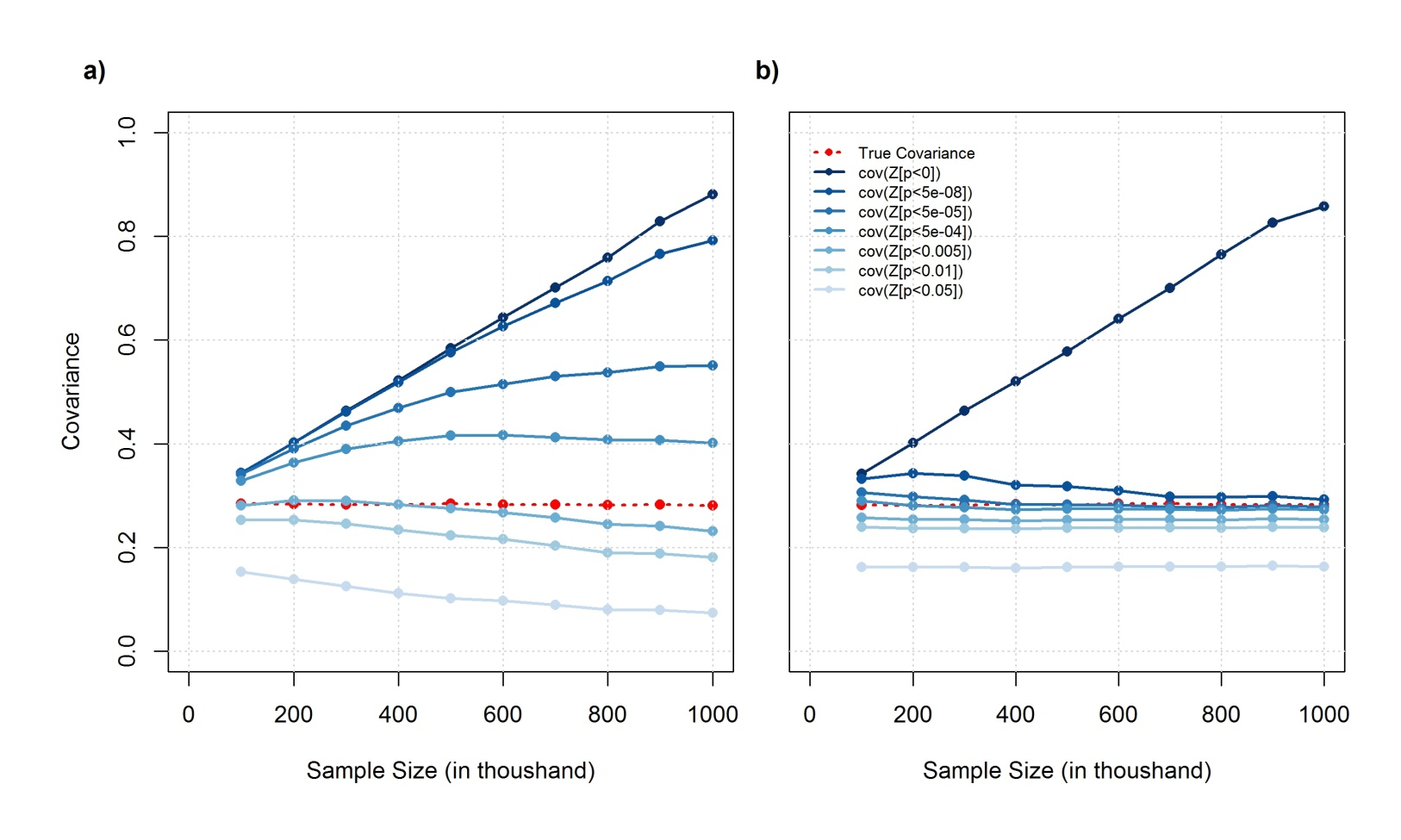

Supplement: S8 Fig — We simulated series of correlated z-scores for 100,000 SNPs from two outcomes Y1 and Y2. For each simulation we generated a matrix of true genetic effect b = (β1 β2) of m standardized and independent genotypes for two phenotypes Y1 and Y2 from a multivariate normal with means of 0 variance h12/m, and h22/m, respectively, and covariance σg, where h12=0.3 and h22=0.6 are the heritability of Y1 and Y2. We then generated b^ defined as b^=b+ε where ε was also drawn from a multivariate normal with means 0, variance 1/N1 and 1/N2, respectively, and covariance reNs/N1N2, where N1, N2 = N1/2 and Ns = N2/2 are the sample sizes for Y1 and Y2, and the number of shared samples, respectively, and re = 0.4 is the correlation between Y1 and Y2 across overlapping samples. Finally, we derived the expected z-score for each genotype z=(β^1N1β^2N2)=(z1z2), and σz = cov(z1, z2), the covariance between z1 and z2. The left panel (a) show the covariance between z-score for null variants in red, and the observed covariance between all z-scores except those harboring a p-value below a given threshold (0 (i.e. no SNP removed), 5x10^-8^, 5x10^-5^, 5x10^-4^, 5x10^-3^, 1x10^-2^, and 5x10^-8^). The right panel (b) shows the same results while assuming only 10% of the variants are causal, while all remaining have b = 0. (TIF) [file pgen.1009713.s009.tif]

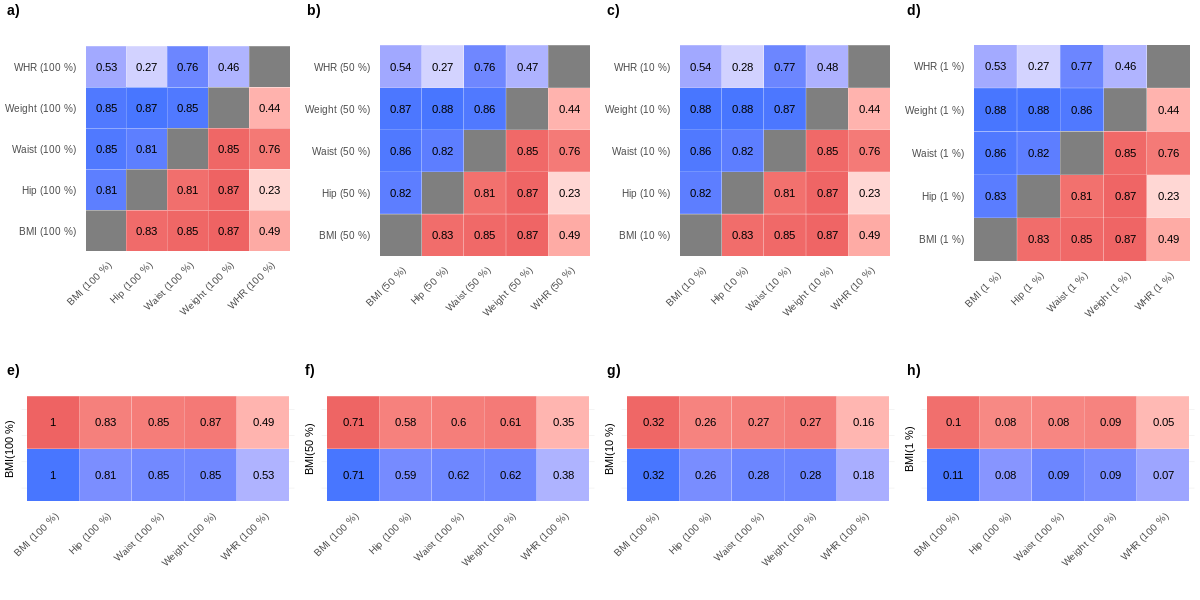

Supplement: S9 Fig — We compared LDSC estimates of covariance between summary statistics against its expected value, which equals ρNs/N1N2, where ρ is the phenotypic correlation among overlapping sample and Ns, N1 and N2 are the sample overlap, the sample size for phenotype 1 and the sample size for phenotype 2, respectively. We used individual-level data for five anthropometric traits and 619,017 SNPS measured in 336,347 individuals from the UK Biobank cohort. We first considered scenarios with complete sample overlap (i.e. Ns = N1 = N2, panels a-d), so that LDSC estimate is expected to equal the phenotypic correlation. We compared estimates while using 100% (a), 50% (b), 10% (c) and 1% (ds) of the total sample size respectively. The lower matrix triangle in pale red are estimated phenotypic correlation from individual-level data, while the upper triangle in pale blue shades are estimated correlation derived using the LDSC. We then considered scenario where sample overlap is only partial by sub-sampling individuals only for BMI, using 100% (e),50% (f), 10% (g) and 1% (h) of the total sample for that phenotype. The first row, in red, is the expected GWAS covariance knowing ρ, Ns, N1 and N2. The second row, in blue, is the estimate derived using LDSC. (TIF) [file pgen.1009713.s010.tif]

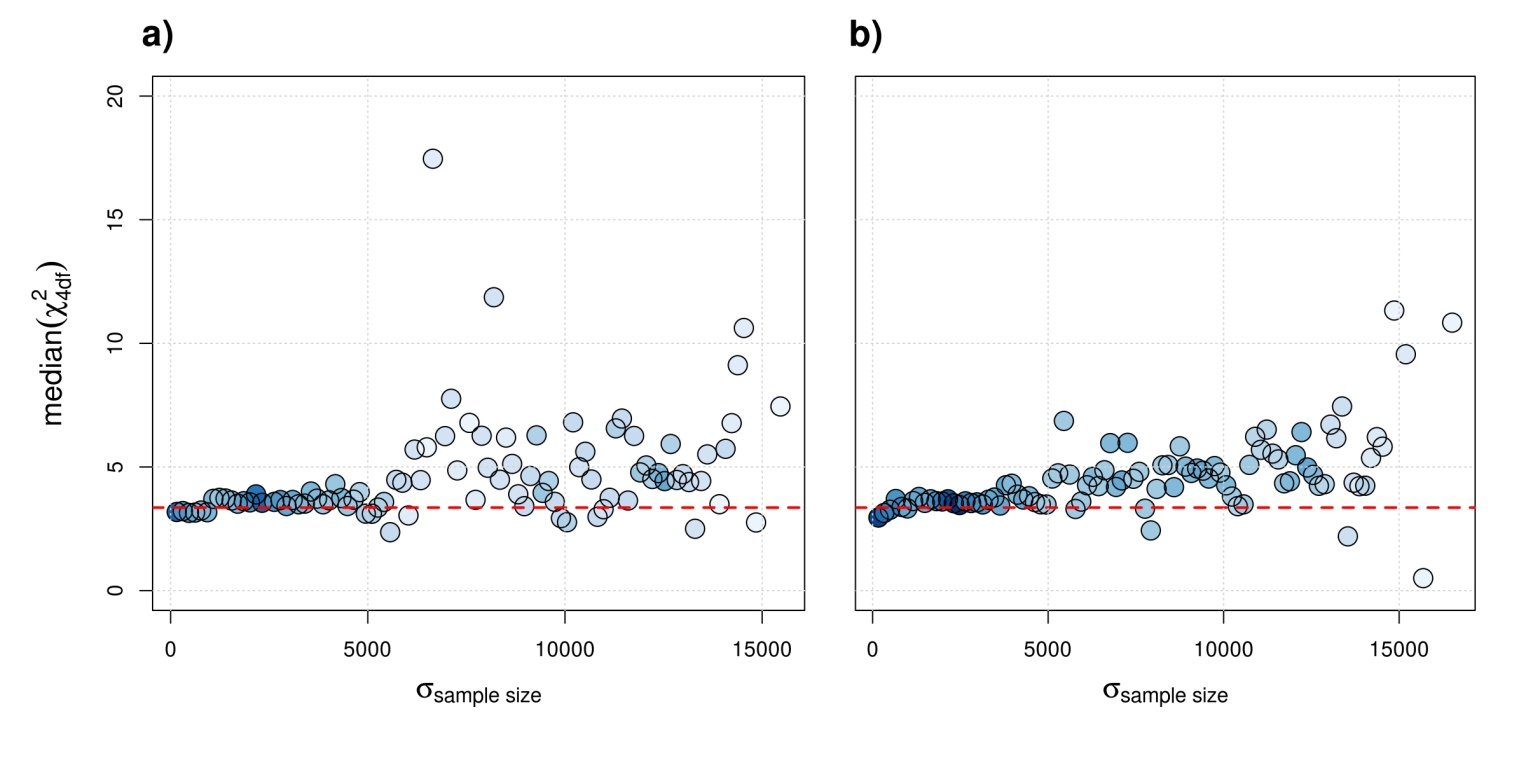

Supplement: S10 Fig — We performed the Omnibus test to the four traits from the GLG consortium: high density lipoprotein (HDL), low density lipoprotein (LDL), total cholesterol (TC), and total triglyceride (TG) using all available SNP with complete summary statistics. We plotted the median chi-squared of the resulting test across bin of SNPs defined based the per-SNP standard deviation in sample size across the four traits (σsample size) for SNPs with a minor allele frequency (MAF) below 5% (a), and above 5% (b). The red dashed line indicates the expected 4 degree of freedom chi-square under the null. The shade of blue is proportional to the number of SNPs in each bin. (TIF) [file pgen.1009713.s011.tif]

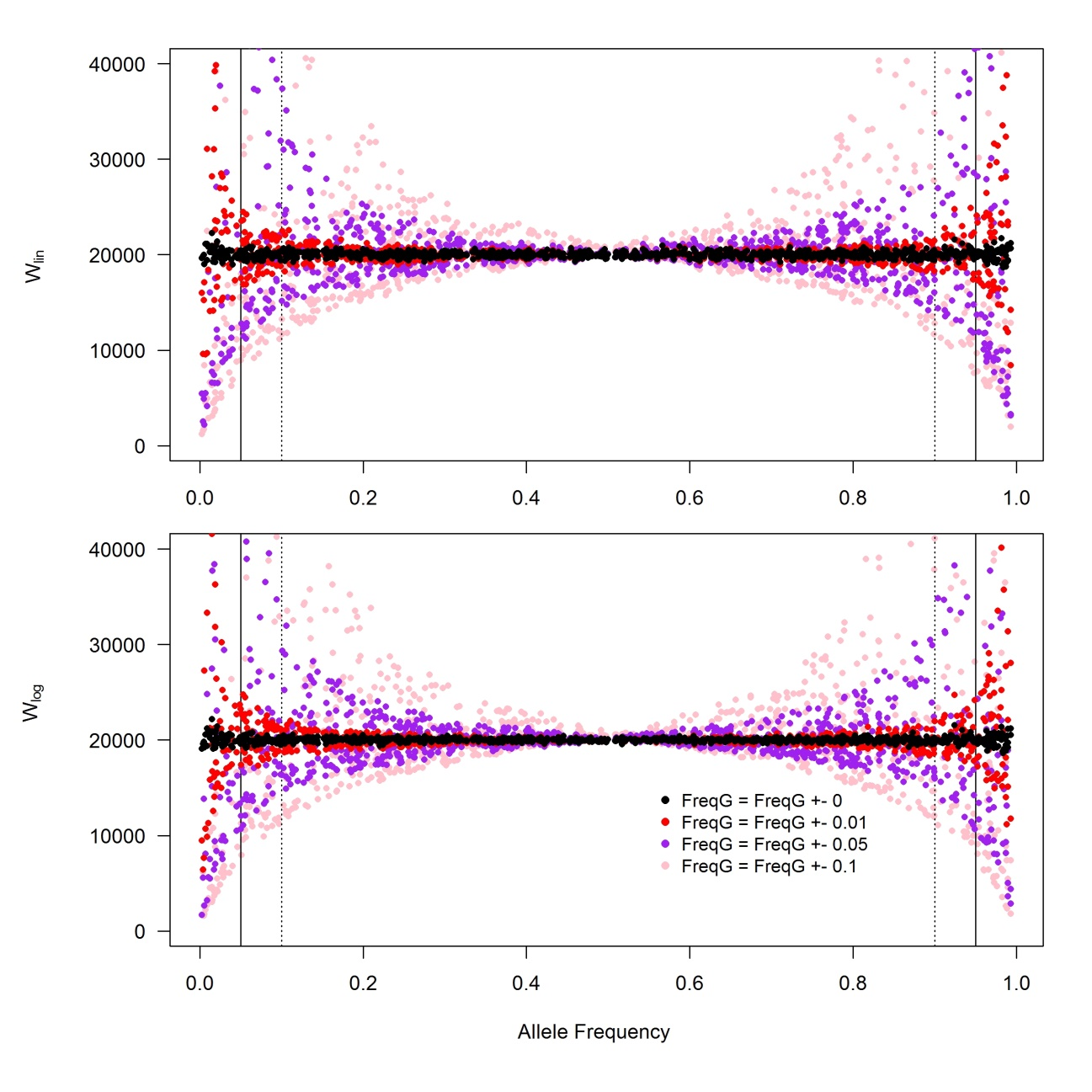

Supplement: S11 Fig — We simulated 1,000 replicates including 20,000 individuals were a phenotype Y is simulated independently of a genotype G with frequency randomly sampled in [0.001, 0.999]. For each replicate we tested for association between G and Y and we inferred w the weights, which equals the sample size times a constant, using the standard error of the effect estimate and either the in-sample allele frequency or the in-sample allele plus a noise term sample from a uniform with min and max in [0.01, 0.05, 0.10]. When allele frequency plus noise was larger or smaller than 0 or 1, the value was set to 0.001 and 0.999, respectively. Upper and lower panels show the inferred sample size as a function of the true allele frequency when using the identity and logit link functions for modelling and testing for association, respectively. Note that for the later, for each replicate, we simulated 50,000 individuals and considered a disease prevalence of 25%. We then randomly sampled 10,000 cases and 10,000 controls to form replicates of 20,000 individuals. Also, for the sake of comparison, we scaled w by a constant in the logit model so that the target is the true sample size. (TIF) [file pgen.1009713.s012.tif]

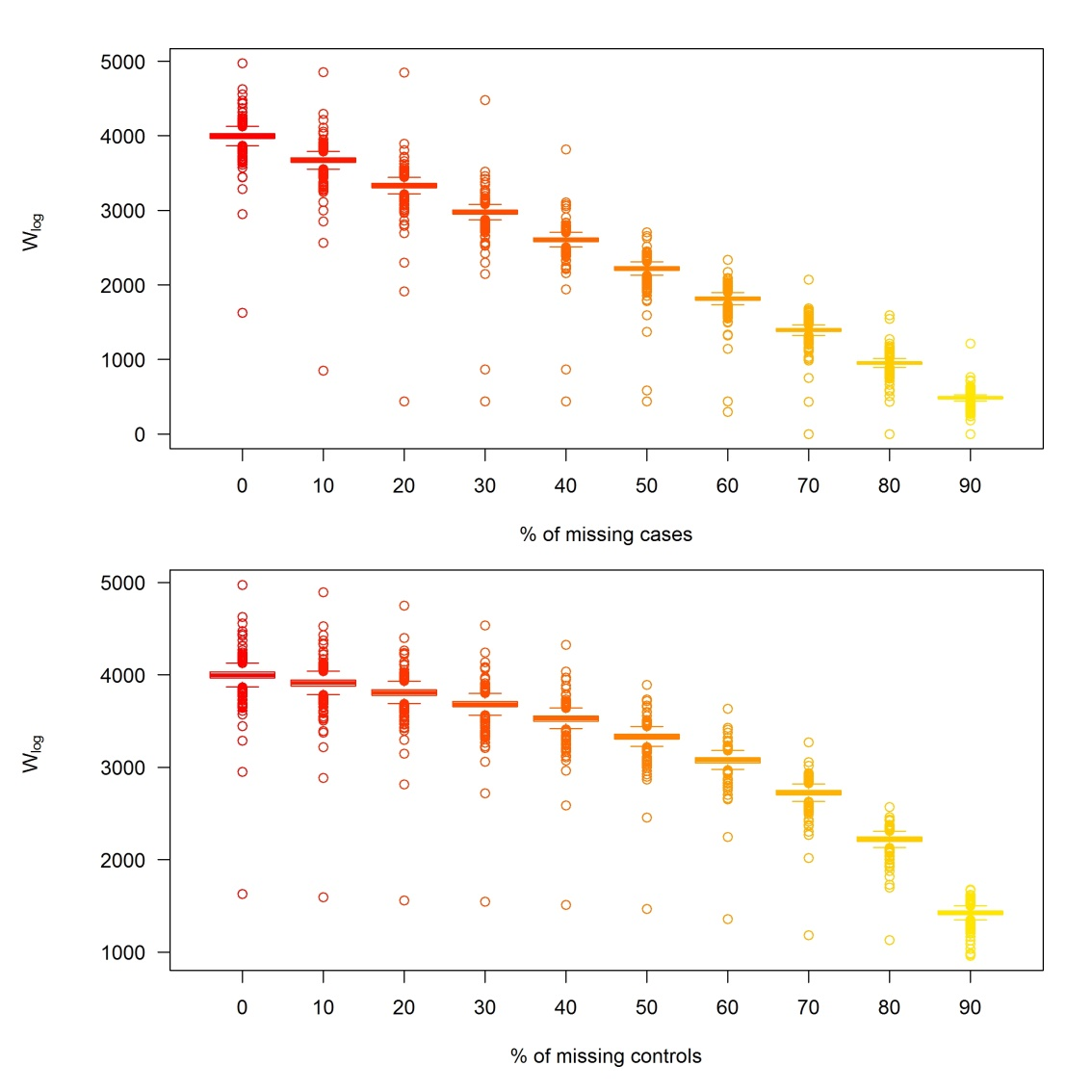

Supplement: S12 Fig — We simulated 1,000 replicates including 50,000 individuals. For each individual we generated a disease status assuming a prevalence of 25% and an independent genotype G with frequency randomly sampled in [0.001, 0.999]. For each replicate we randomly sampled 5,000 cases and 20,000 controls and tested for association between G and Y using these samples and after sub-sampling cases (i.e. using a sub-sample of the 5,000 cases and the 20,000 controls, top panel) or controls (i.e. using the 5,000 cases and a sub-sample of the 20,000 controls, bottom panel), in order to mimic situation where either cases or controls would be missing for some SNPs. For each experiment we inferred Wlog = Np(1−p), where N is the sample size and p is the case-control ratio, using the standard error of the effect estimate and the in-sample allele frequency. In both situations (sub-sampling cases or sub-sampling controls), Wlog is decreasing with increasing percentage of missingness. (TIF) [file pgen.1009713.s013.tif]

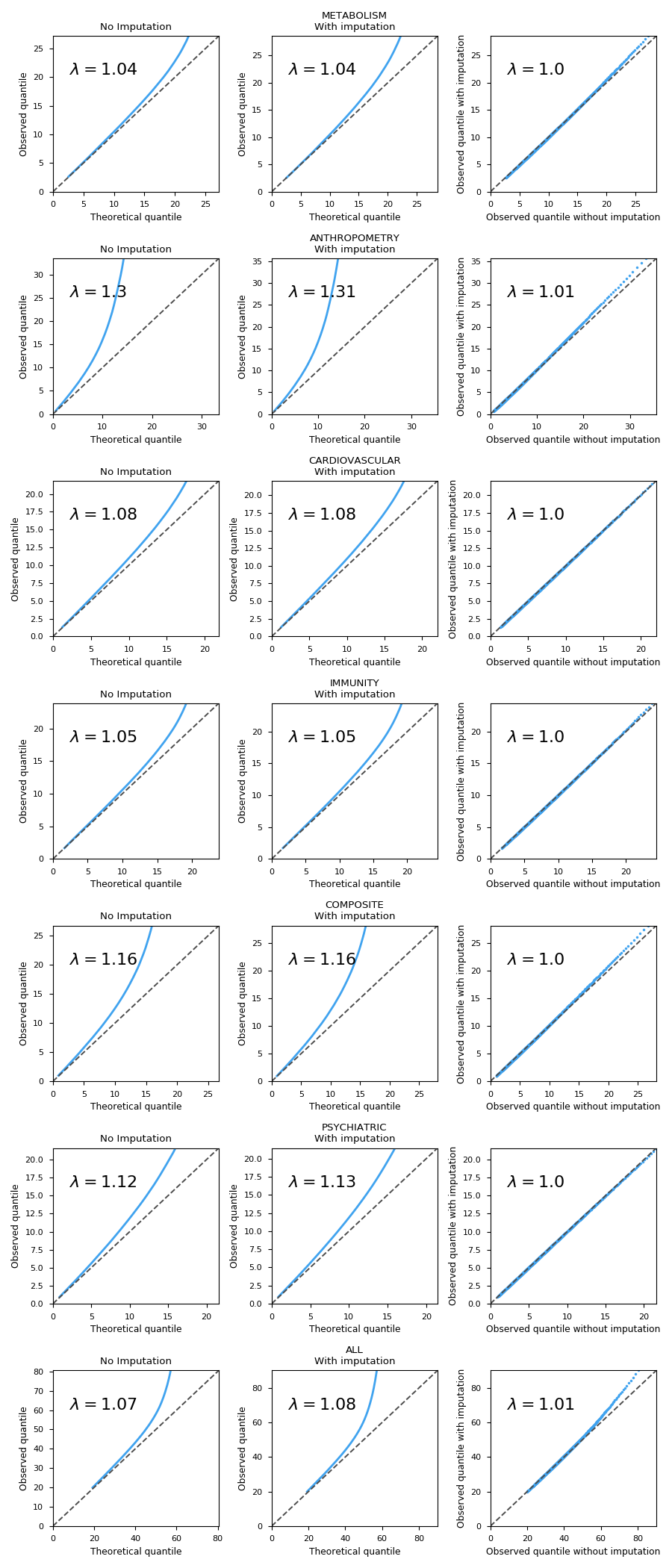

Supplement: S13 Fig — Each line corresponds to a GWAS set. The left column is the empirical quantile of the Omnibus statistic versus the theoretical quantile before imputation. The middle column is the same after imputation. The right column is the empirical quantile of the Omnibus statistic before imputation versus the same quantity after imputation. (TIF) [file pgen.1009713.s014.tif]

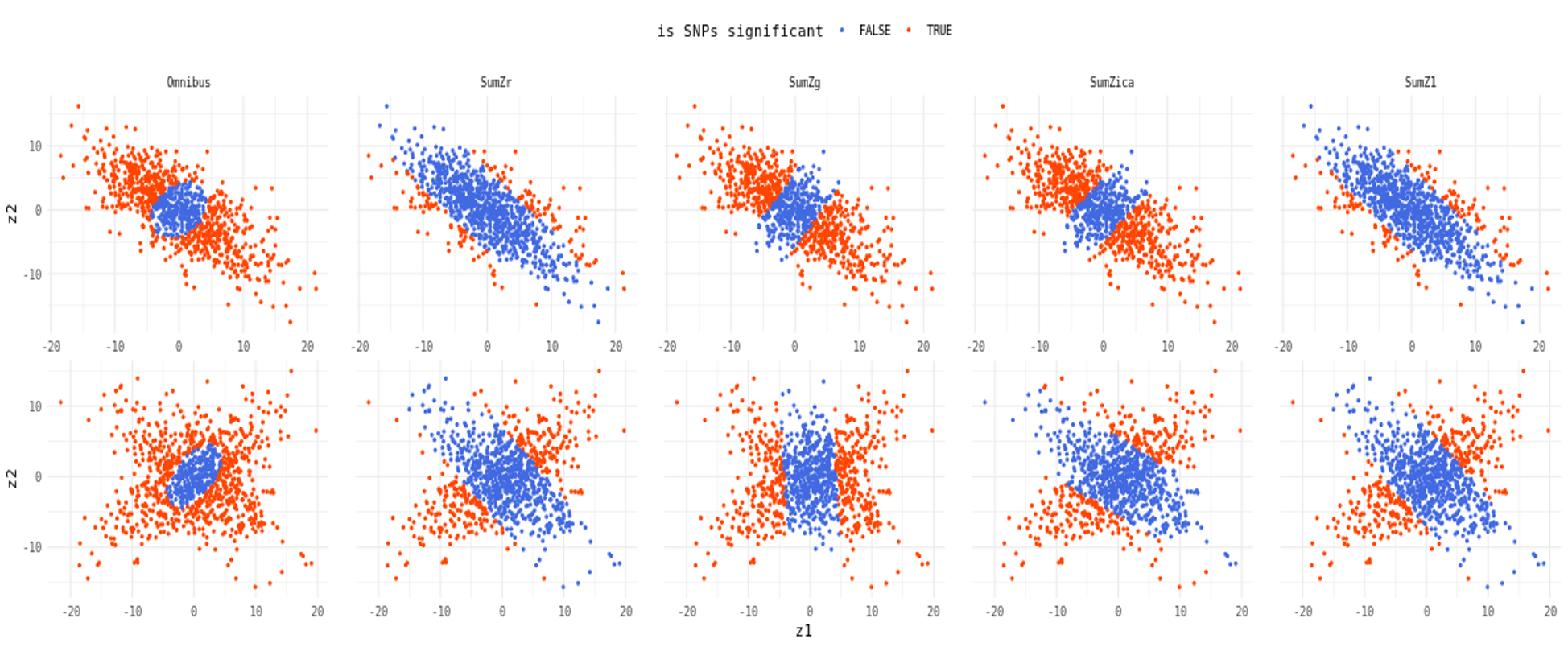

Supplement: S14 Fig — We simulated series of N = 100K individuals with two correlated outcomes Y1 and Y2 as a function of m = 1000 independent SNPs. The genetic effect of the m variants on Y1 and Y1, denoted b = (β1 β2), were drawn from a bivariate normal with means 0, variance h12/m, and h22/m, and correlation σg, where h12=0.4,h22=0.25, and σg = 0.8. The vectors of residual ε = (ε1, ε2) were drawn from a multivariate normal with means 0, variance 1−h12 and 1−h22, respectively, and covariance re = 0.5. For each simulation, we derived the z-score for each of the m genotypes z=(β^1Nβ^2N)=(z1z2) and plotted z1 as a function of z2. We applied the five tests: Omnibus, sumZr, sumZg, sumZica and sumZ1 and highlighted for each of them whether variants had significant p-value (red, p<5x10^-5^) or not (blue, p>5x10^-5^). In the results from the upper panels, b was drawn from a bivariate normal distribution with the σg parameter was set to 0.24. For the lower panel, b was drawn from a mixture of two normal distributions both centered on zero and both with variance equal to h12/m, and h22/m. However, the covariance σg was set to 0.8 for the first one and -0.65 for the second one. (TIF) [file pgen.1009713.s015.tif]

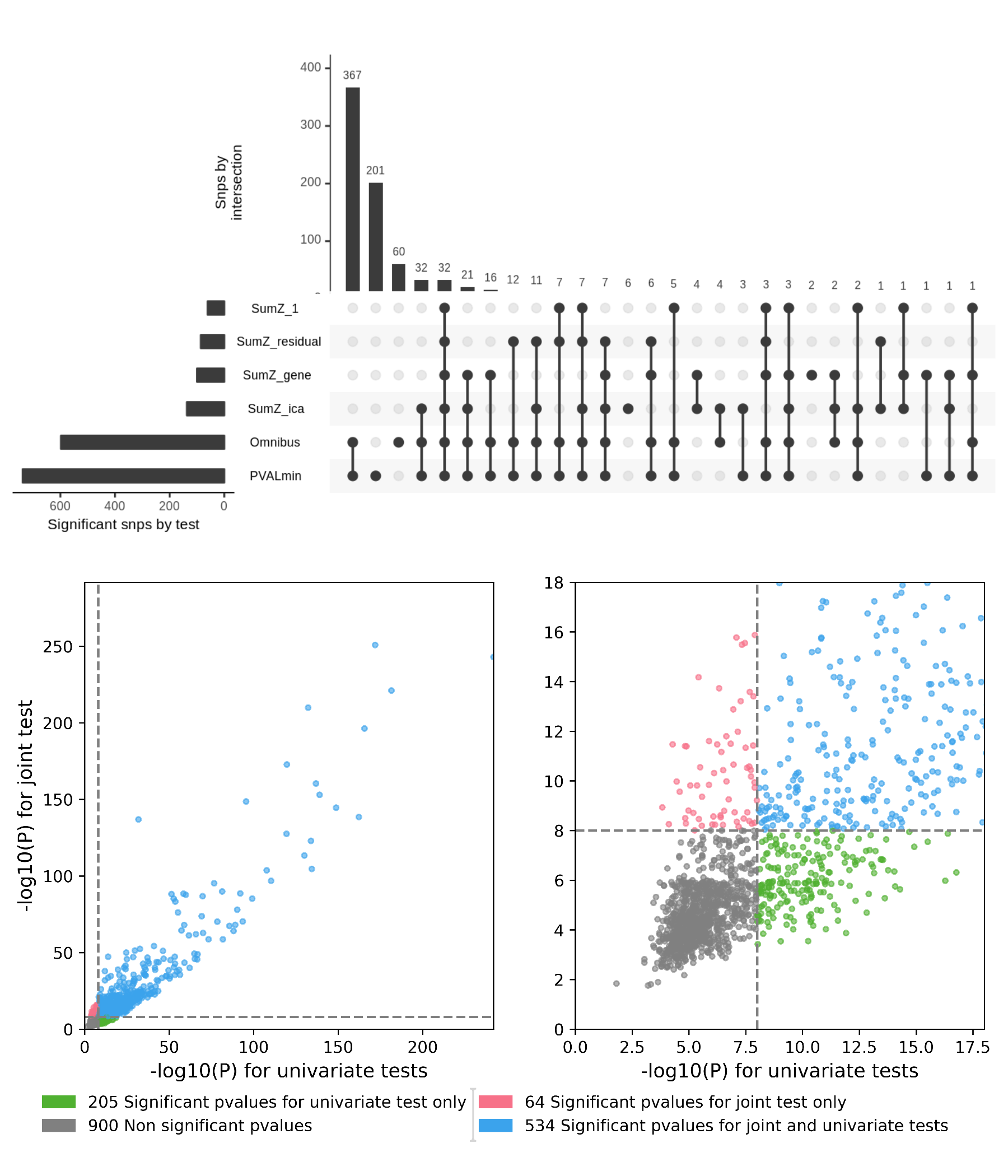

Supplement: S15 Fig — The upper panel shows independent variants detected across phenotype groups and across approaches represented as an UpSetR visualization. Matrix lines correspond to a test, each column to a set of significant variants. For each set, the test for which variants are significant are represented with a black dot on the test line. The barplot on the left of the matrix represents the number of significant independent signals detected by each approach. The barplot on the top of the matrix represents the cardinality of the sets. The sets are ordered by cardinality from the largest to the leftmost to the smallest to the rightmost. The bottom panels show quadrant plots, i.e. the -log10(p-value) for the most significant SNP per region for the Omnibus test as a function of the -log10(p-value) for the most significant SNP per region across all univariate GWAS. Complete results are presented in the left panel, and a zoom around the genome-wide significance threshold is presented on the right panel. (TIF) [file pgen.1009713.s016.tif]

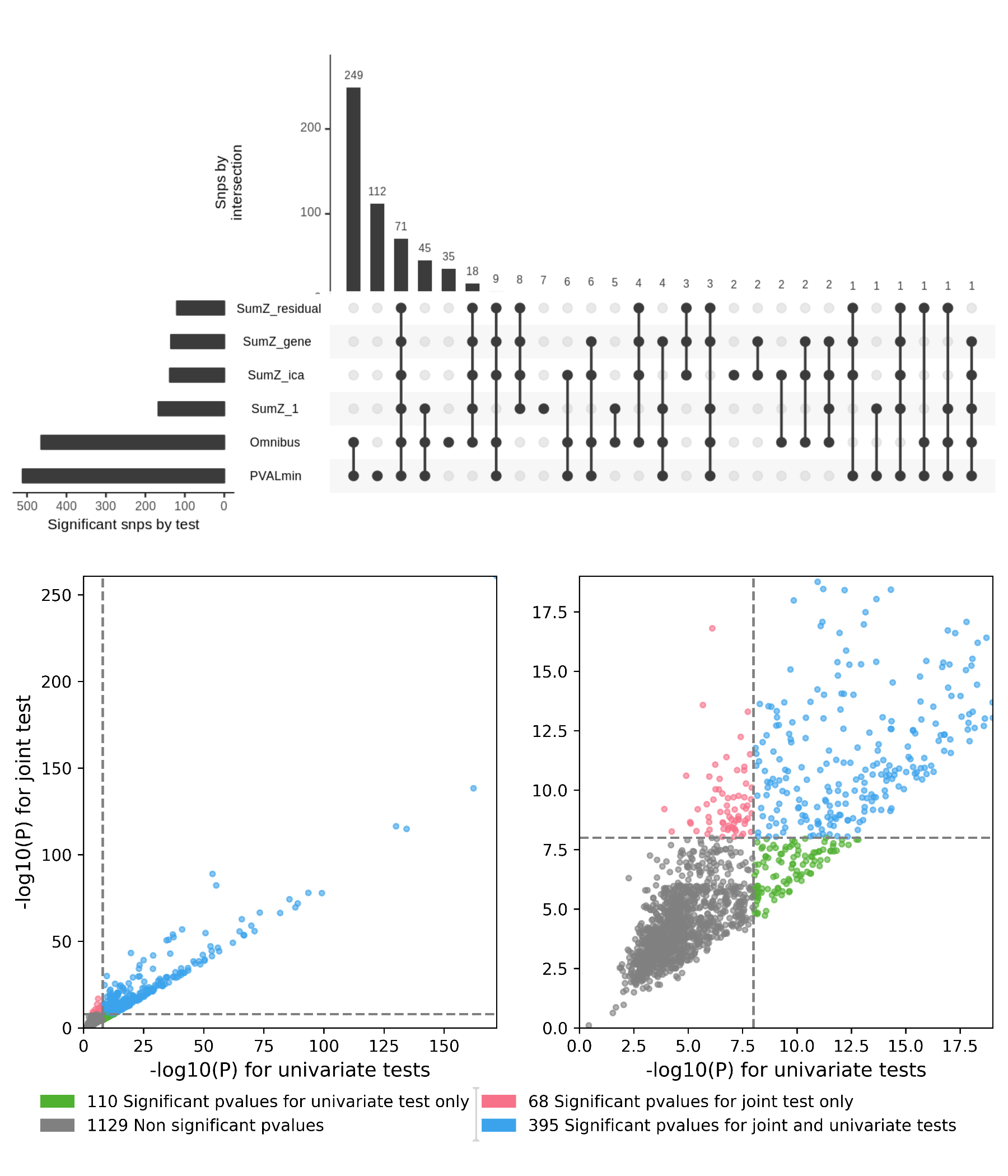

Supplement: S16 Fig — The upper panel shows independent variants detected across phenotype groups and across approaches represented as an UpSetR visualization. Matrix lines correspond to a test, each column to a set of significant variants. For each set, the test for which variants are significant are represented with a black dot on the test line. The barplot on the left of the matrix represents the number of significant independent signals detected by each approach. The barplot on the top of the matrix represents the cardinality of the sets. The sets are ordered by cardinality from the largest to the leftmost to the smallest to the rightmost. The bottom panels show quadrant plots, i.e. the -log10(p-value) for the most significant SNP per region for the Omnibus test as a function of the -log10(p-value) for the most significant SNP per region across all univariate GWAS. Complete results are presented in the left panel, and a zoom around the genome-wide significance threshold is presented on the right panel. (TIF) [file pgen.1009713.s017.tif]

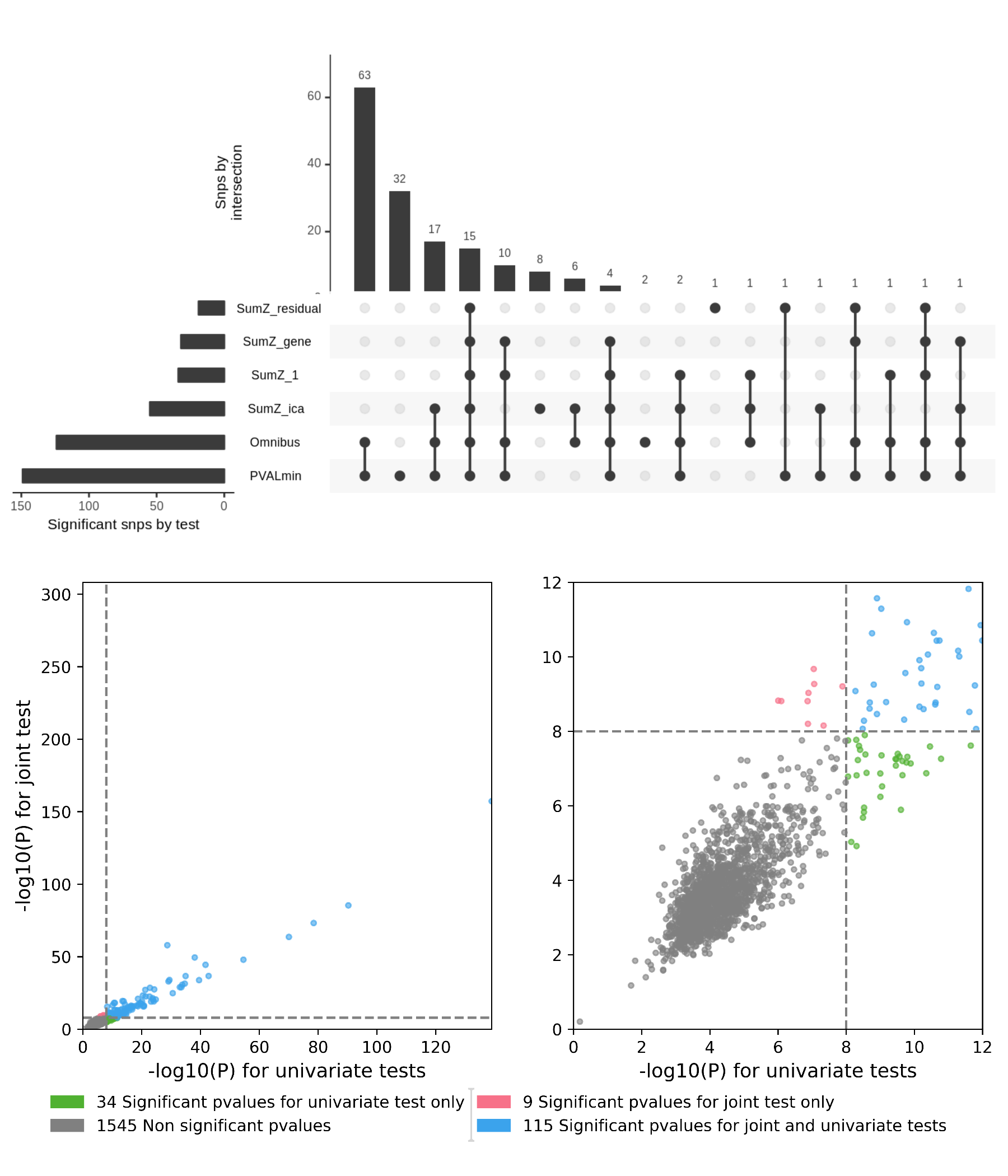

Supplement: S17 Fig — The upper panel shows independent variants detected across phenotype groups and across approaches represented as an UpSetR visualization. Matrix lines correspond to a test, each column to a set of significant variants. For each set, the test for which variants are significant are represented with a black dot on the test line. The barplot on the left of the matrix represents the number of significant independent signals detected by each approach. The barplot on the top of the matrix represents the cardinality of the sets. The sets are ordered by cardinality from the largest to the leftmost to the smallest to the rightmost. The bottom panels show quadrant plots, i.e. the -log10(p-value) for the most significant SNP per region for the Omnibus test as a function of the -log10(p-value) for the most significant SNP per region across all univariate GWAS. Complete results are presented in the left panel, and a zoom around the genome-wide significance threshold is presented on the right panel. (TIF) [file pgen.1009713.s018.tif]

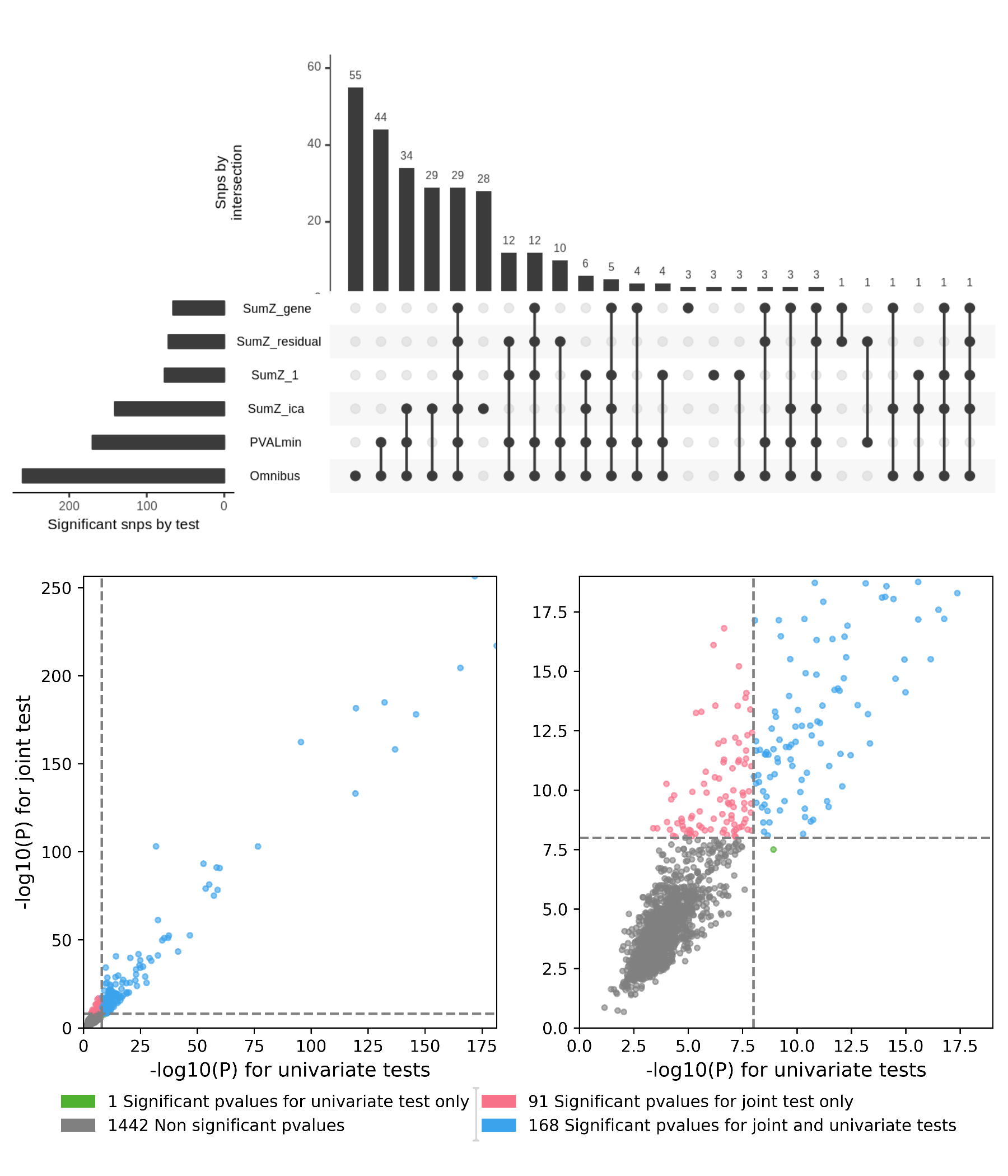

Supplement: S18 Fig — The upper panel shows independent variants detected across phenotype groups and across approaches represented as an UpSetR visualization. Matrix lines correspond to a test, each column to a set of significant variants. For each set, the test for which variants are significant are represented with a black dot on the test line. The barplot on the left of the matrix represents the number of significant independent signals detected by each approach. The barplot on the top of the matrix represents the cardinality of the sets. The sets are ordered by cardinality from the largest to the leftmost to the smallest to the rightmost. The bottom panels show quadrant plots, i.e. the -log10(p-value) for the most significant SNP per region for the Omnibus test as a function of the -log10(p-value) for the most significant SNP per region across all univariate GWAS. Complete results are presented in the left panel, and a zoom around the genome-wide significance threshold is presented on the right panel. (TIF) [file pgen.1009713.s019.tif]

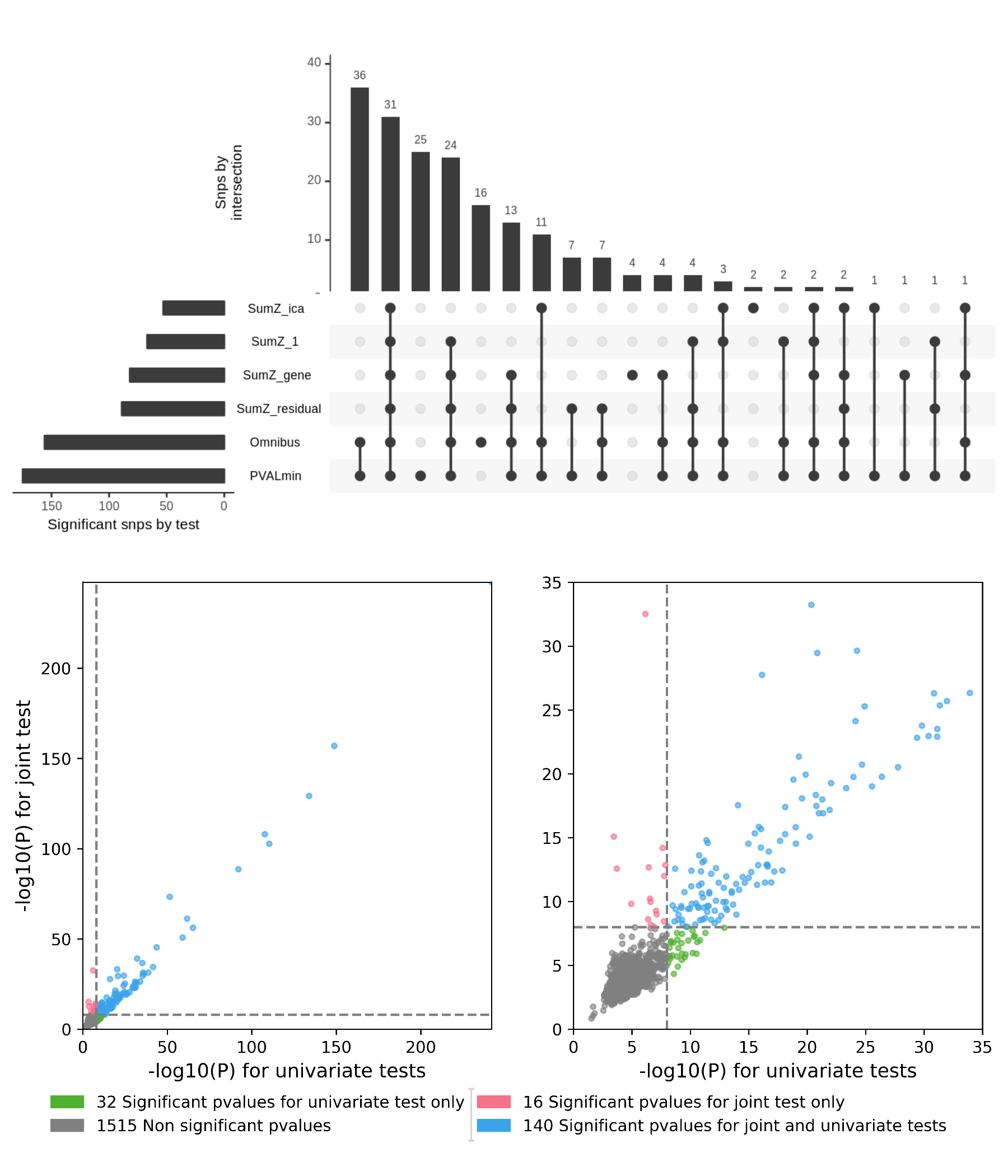

Supplement: S19 Fig — The upper panel shows independent variants detected across phenotype groups and across approaches represented as an UpSetR visualization. Matrix lines correspond to a test, each column to a set of significant variants. For each set, the test for which variants are significant are represented with a black dot on the test line. The barplot on the left of the matrix represents the number of significant independent signals detected by each approach. The barplot on the top of the matrix represents the cardinality of the sets. The sets are ordered by cardinality from the largest to the leftmost to the smallest to the rightmost. The bottom panels show quadrant plots, i.e. the -log10(p-value) for the most significant SNP per region for the Omnibus test as a function of the -log10(p-value) for the most significant SNP per region across all univariate GWAS. Complete results are presented in the left panel, and a zoom around the genome-wide significance threshold is presented on the right panel. (TIF) [file pgen.1009713.s020.tif]

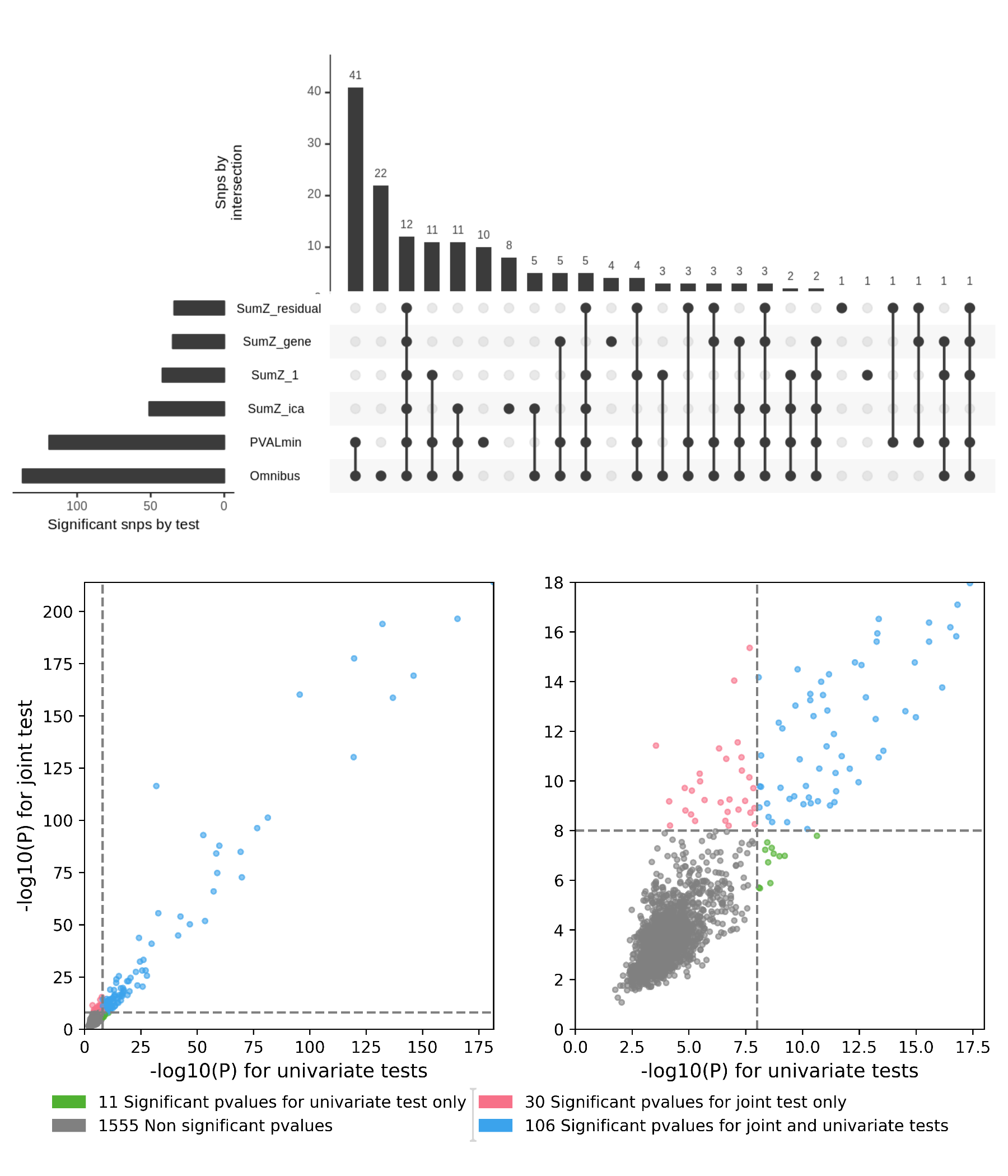

Supplement: S20 Fig — The upper panel shows independent variants detected across phenotype groups and across approaches represented as an UpSetR visualization. Matrix lines correspond to a test, each column to a set of significant variants. For each set, the test for which variants are significant are represented with a black dot on the test line. The barplot on the left of the matrix represents the number of significant independent signals detected by each approach. The barplot on the top of the matrix represents the cardinality of the sets. The sets are ordered by cardinality from the largest to the leftmost to the smallest to the rightmost. The bottom panels show quadrant plots, i.e. the -log10(p-value) for the most significant SNP per region for the Omnibus test as a function of the -log10(p-value) for the most significant SNP per region across all univariate GWAS. Complete results are presented in the left panel, and a zoom around the genome-wide significance threshold is presented on the right panel. (TIF) [file pgen.1009713.s021.tif]

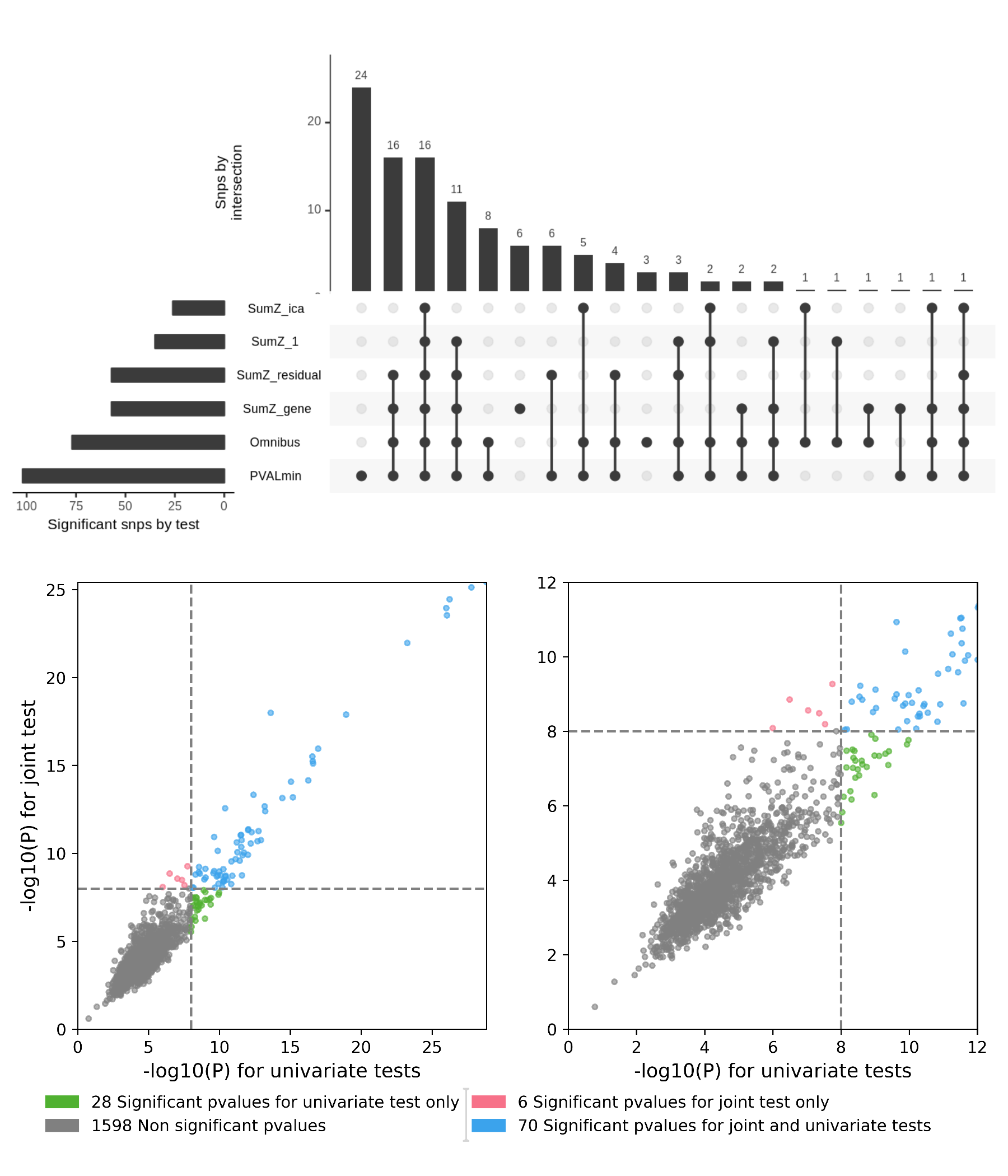

Supplement: S21 Fig — The upper panel shows independent variants detected across phenotype groups and across approaches represented as an UpSetR visualization. Matrix lines correspond to a test, each column to a set of significant variants. For each set, the test for which variants are significant are represented with a black dot on the test line. The barplot on the left of the matrix represents the number of significant independent signals detected by each approach. The barplot on the top of the matrix represents the cardinality of the sets. The sets are ordered by cardinality from the largest to the leftmost to the smallest to the rightmost. The bottom panels show quadrant plots, i.e. the -log10(p-value) for the most significant SNP per region for the Omnibus test as a function of the -log10(p-value) for the most significant SNP per region across all univariate GWAS. Complete results are presented in the left panel, and a zoom around the genome-wide significance threshold is presented on the right panel. (TIF) [file pgen.1009713.s022.tif]

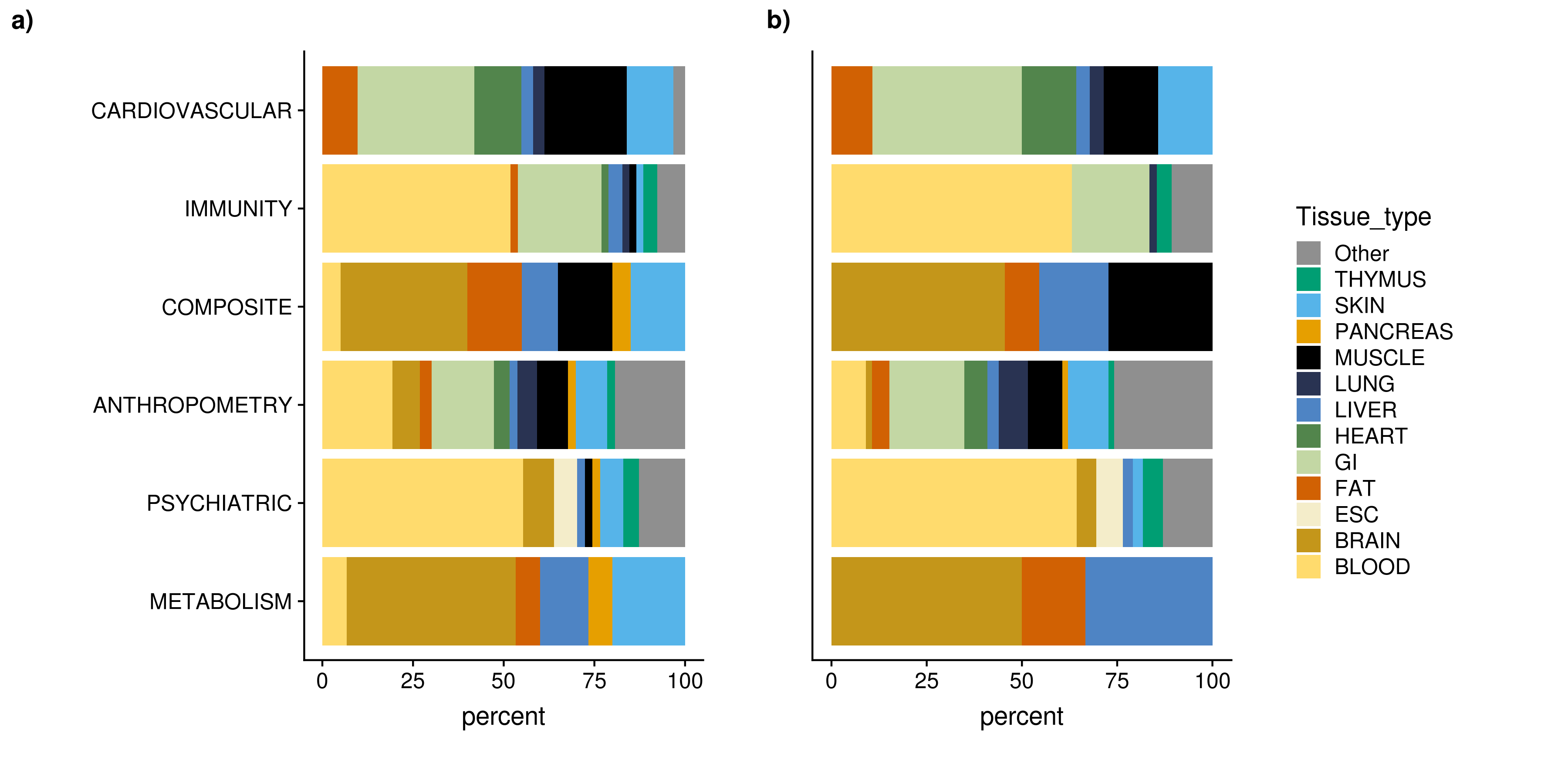

Supplement: S22 Fig — The union of enriched tissue by phenotype set were mapped to their larger anatomical category (Tissue type) in GTEx. To simplify the visualization, anatomical categories that did not explain at least 5% of any of the phenotype group were regrouped under the “Other” category. In each group, anatomical category representing less than 1% were approximated to 0. a) results obtained with variants detected with univariate test and b) results with variants detected with multivariate tests. (TIF) [file pgen.1009713.s023.tif]

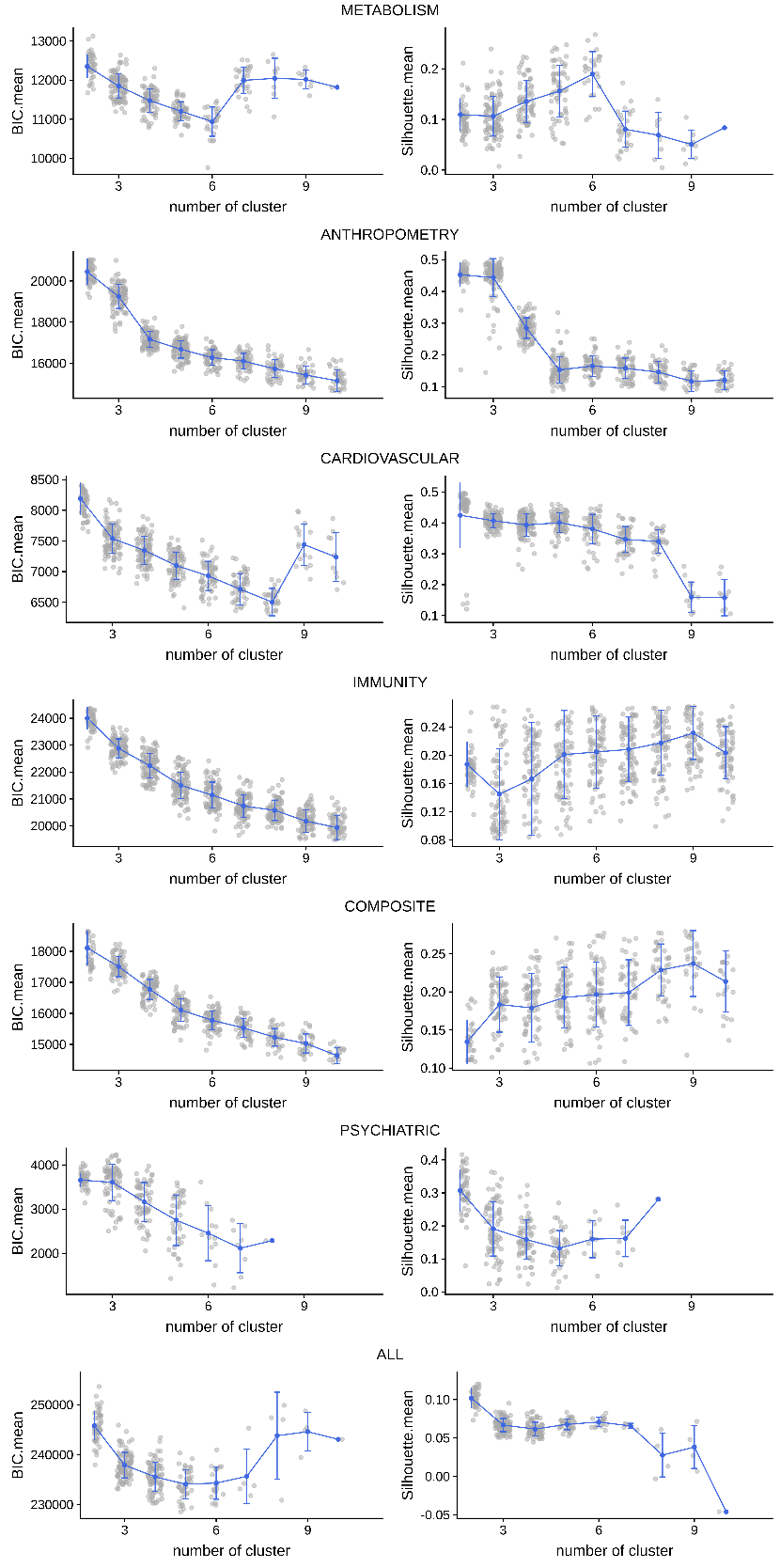

Supplement: S23 Fig — In all panels, the x-axis is the number of clusters derived using the union of significant SNPs in the Omnibus, SumZgenet and univariate tests. On the left column, the y-axis represents the BIC (Bayesian Information Criteria). On the right column, the y-axis is the Silhouette criteria (see Methods). Each line corresponds to a different group of phenotypes. (TIF) [file pgen.1009713.s024.tif]

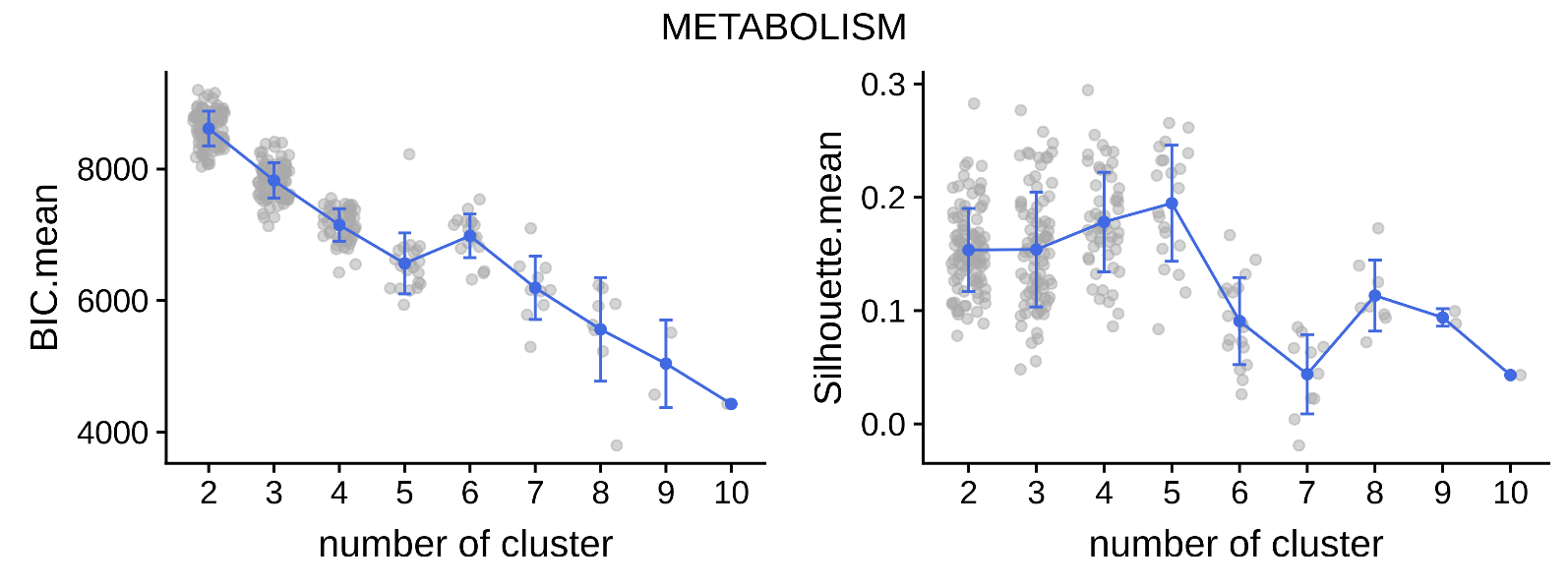

Supplement: S24 Fig — The x-axis is the number of clusters derived using the significant SNPs univariate tests. On the left column, the y-axis represents the BIC (Bayesian Information Criteria). On the right column, the y-axis is the Silhouette criteria (see Methods). (TIF) [file pgen.1009713.s025.tif]

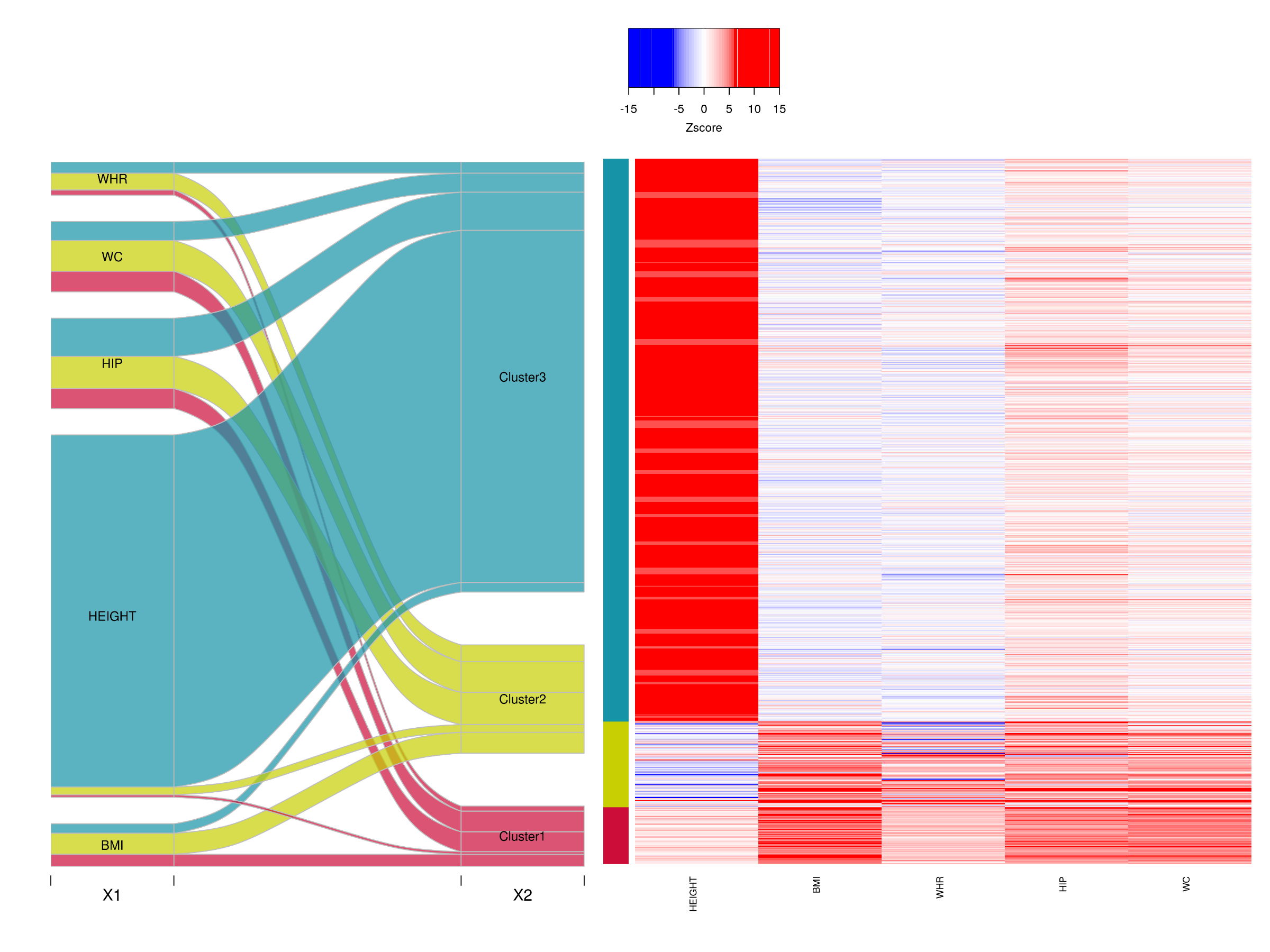

Supplement: S25 Fig — On the left panel, the alluvial plot represents the re-assignment of SNPs from univariate analysis to clusters. To emphasize the relative genetic contribution to phenotypes, SNPs from each phenotype block were weighted by their variance explained to that phenotype. On the right panel, the heatmap represents the multi-trait signatures. Each line is a SNPs, each column, a trait. The gradient of color represents the strength of the Z-scores. (TIF) [file pgen.1009713.s026.tif]

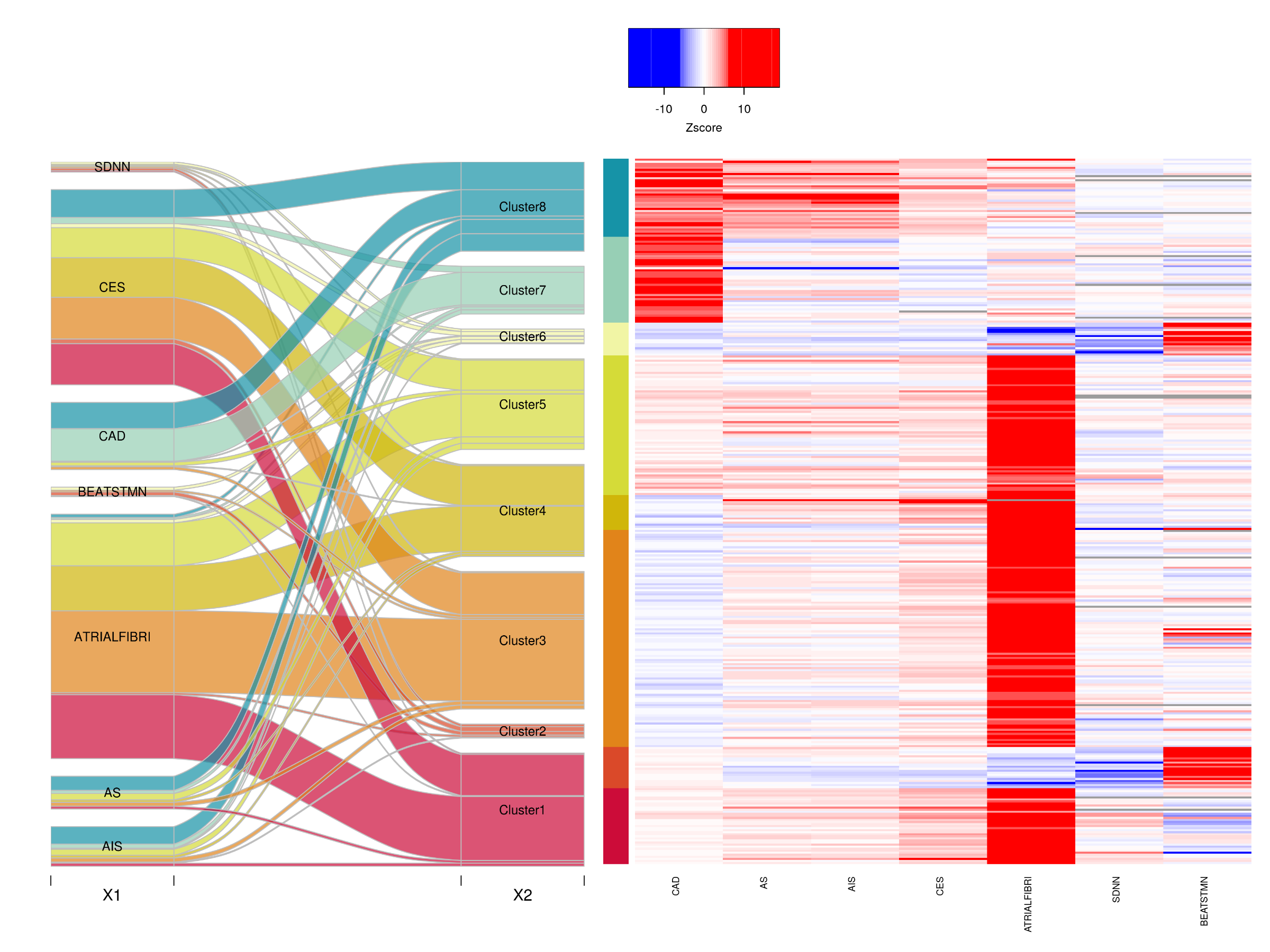

Supplement: S26 Fig — On the left panel, the alluvial plot represents the re-assignment of SNPs from univariate analysis to clusters. To emphasize the relative genetic contribution to phenotypes, SNPs from each phenotype block were weighted by their variance explained to that phenotype. On the right panel, the heatmap represents the multi-trait signatures. Each line is a SNP, each column, a trait. The gradient of color represents the strength of the Z-scores. (TIF) [file pgen.1009713.s027.tif]

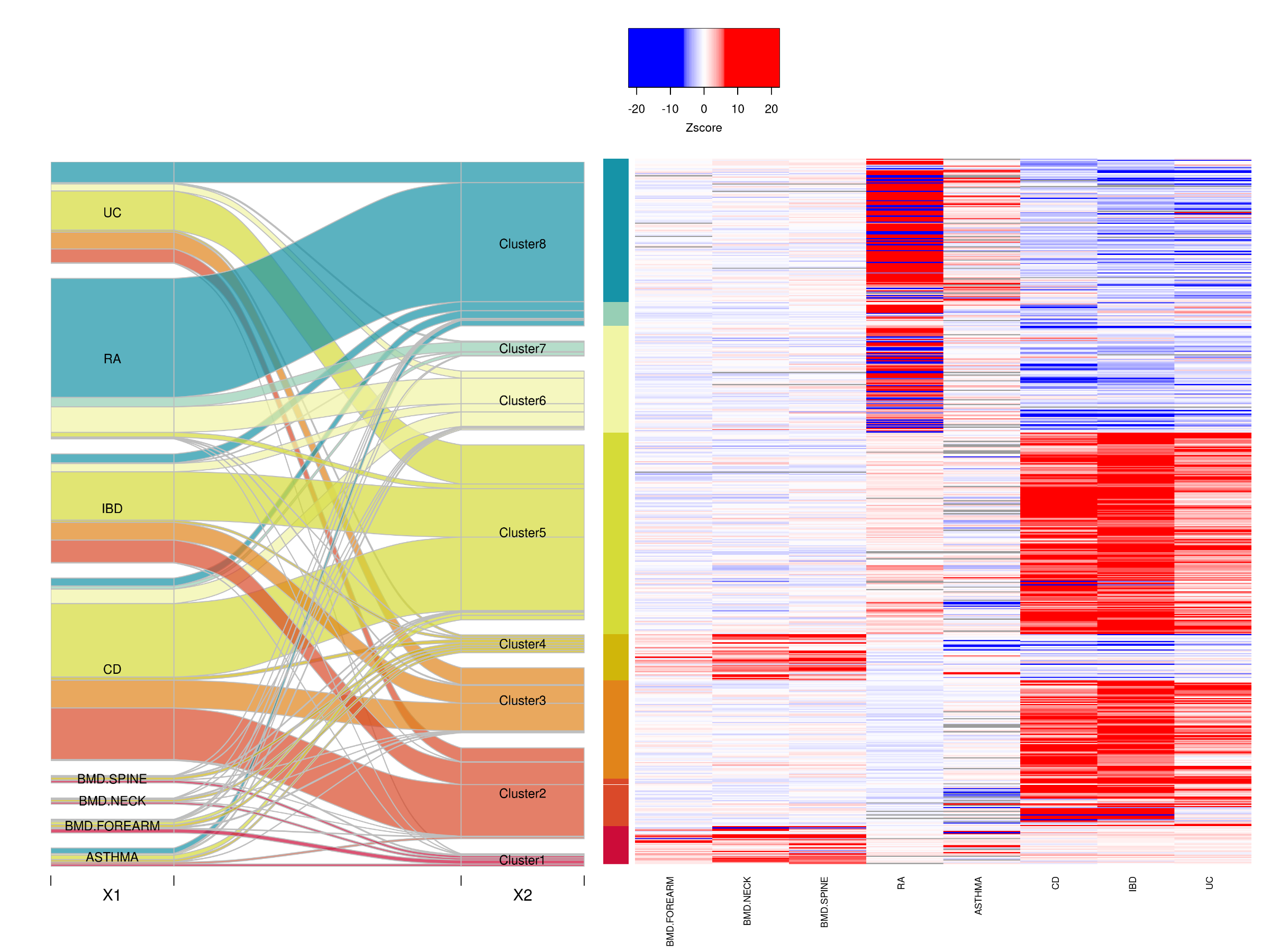

Supplement: S27 Fig — On the left panel, the alluvial plot represents the re-assignment of SNPs from univariate analysis to clusters. To emphasize the relative genetic contribution to phenotypes, SNPs from each phenotype block were weighted by their variance explained to that phenotype. On the right panel, the heatmap represents the multi-trait signatures. Each line is a SNP, each column, a trait. The gradient of color represents the strength of the Z-scores. (TIF) [file pgen.1009713.s028.tif]

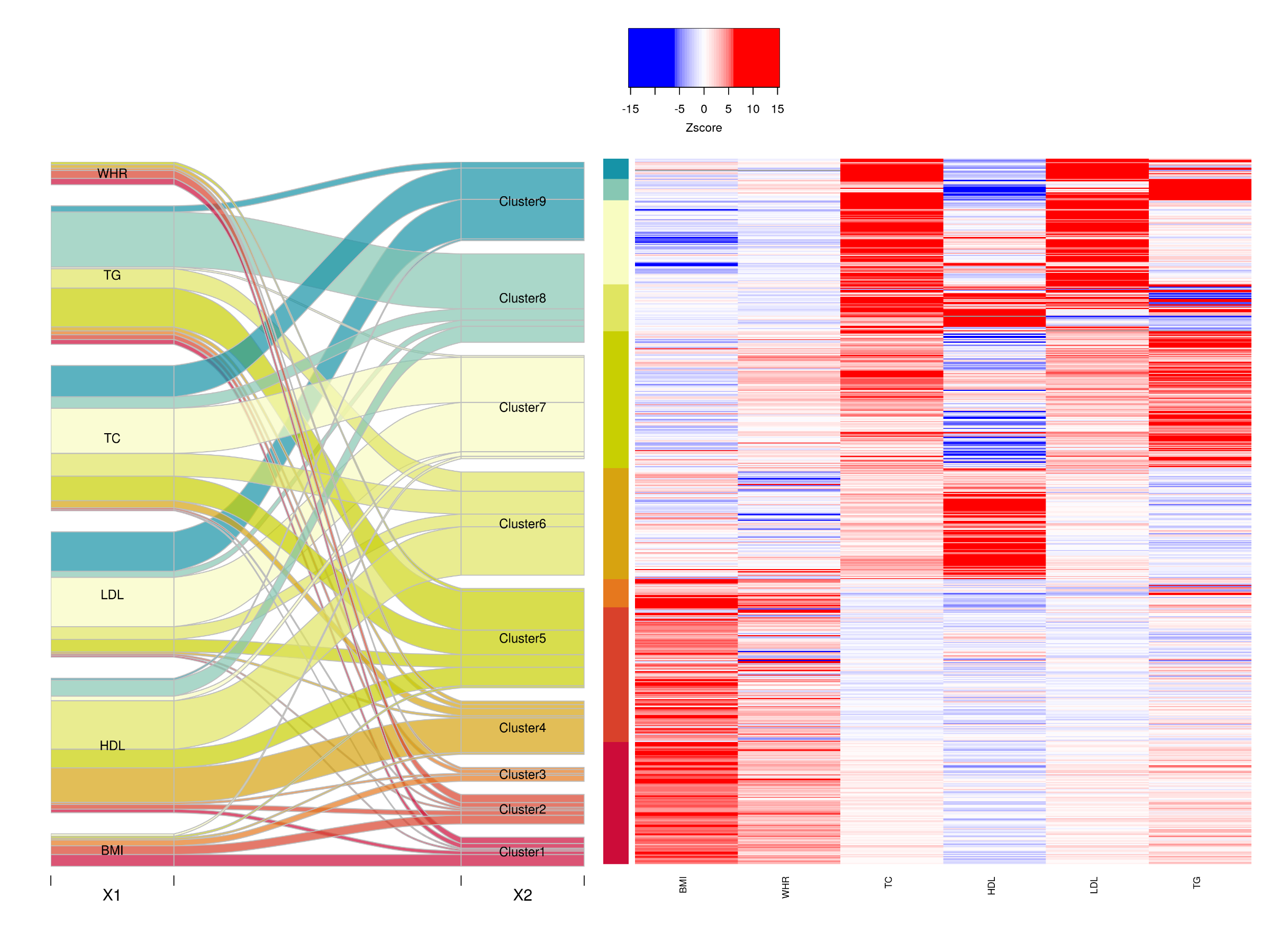

Supplement: S28 Fig — On the left panel, the alluvial plot represents the re-assignment of SNPs from univariate analysis to clusters. To emphasize the relative genetic contribution to phenotypes, SNPs from each phenotype block were weighted by their variance explained to that phenotype. On the right panel, the heatmap represents the multi-trait signatures. Each line is a SNP, each column, a trait. The gradient of color represents the strength of the Z-scores. (TIF) [file pgen.1009713.s029.tif]

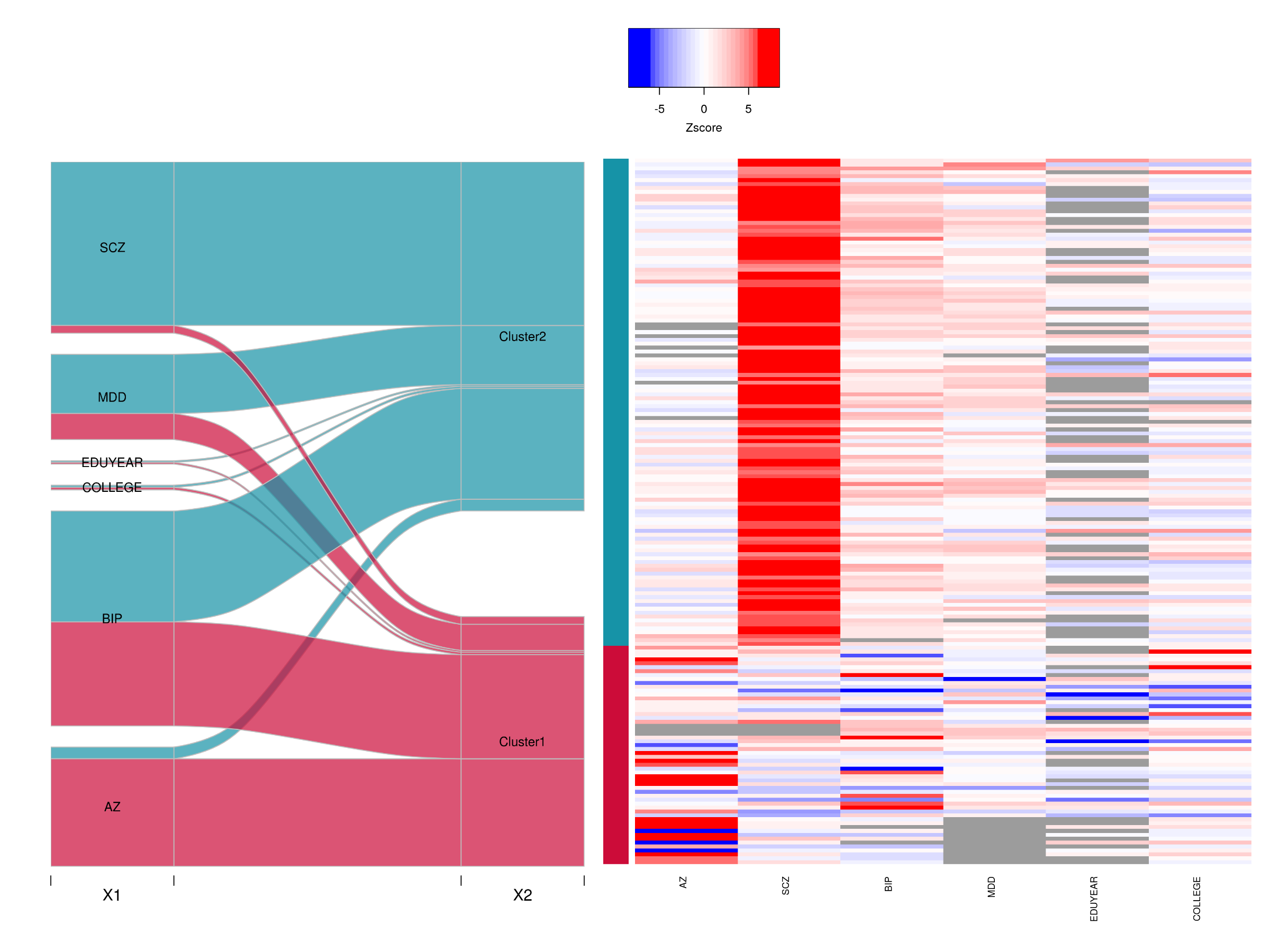

Supplement: S29 Fig — On the left panel, the alluvial plot represents the re-assignment of SNPs from univariate analysis to clusters. To emphasize the relative genetic contribution to phenotypes, SNPs from each phenotype block were weighted by their variance explained to that phenotype. On the right panel, the heatmap represents the multi-trait signatures. Each line is a SNP, each column, a trait. The gradient of color represents the strength of the Z-scores. (TIF) [file pgen.1009713.s030.tif]

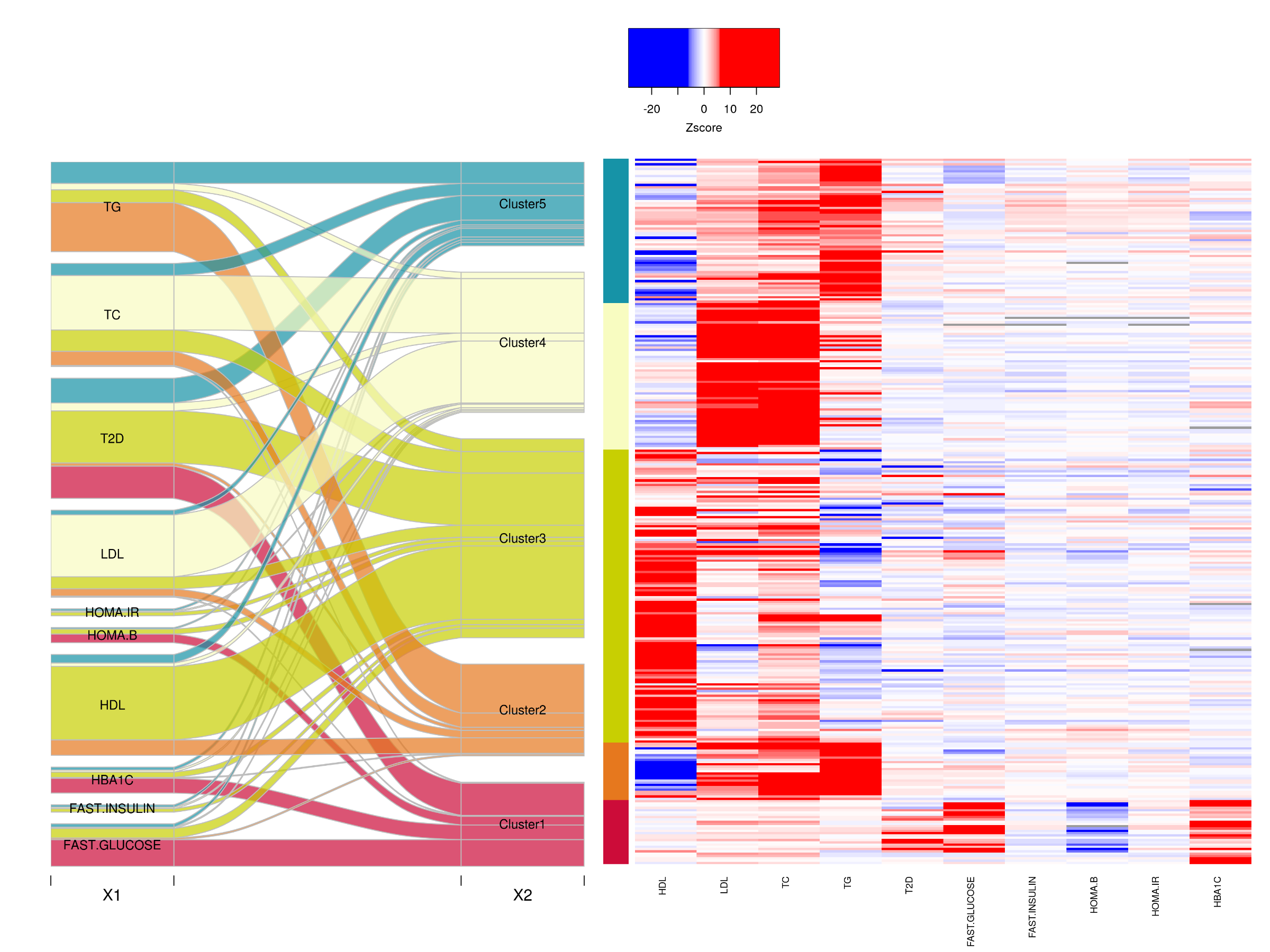

Supplement: S30 Fig — On the left panel, the alluvial plot represents the re-assignment of SNPs from univariate analysis to clusters. To emphasize the relative genetic contribution to phenotypes, SNPs from each phenotype block were weighted by their variance explained to that phenotype. On the right panel, the heatmap represents the multi-trait signatures. Each line is a SNP, each column, a trait. The gradient of color represents the strength of the Z-scores. (TIF) [file pgen.1009713.s031.tif]

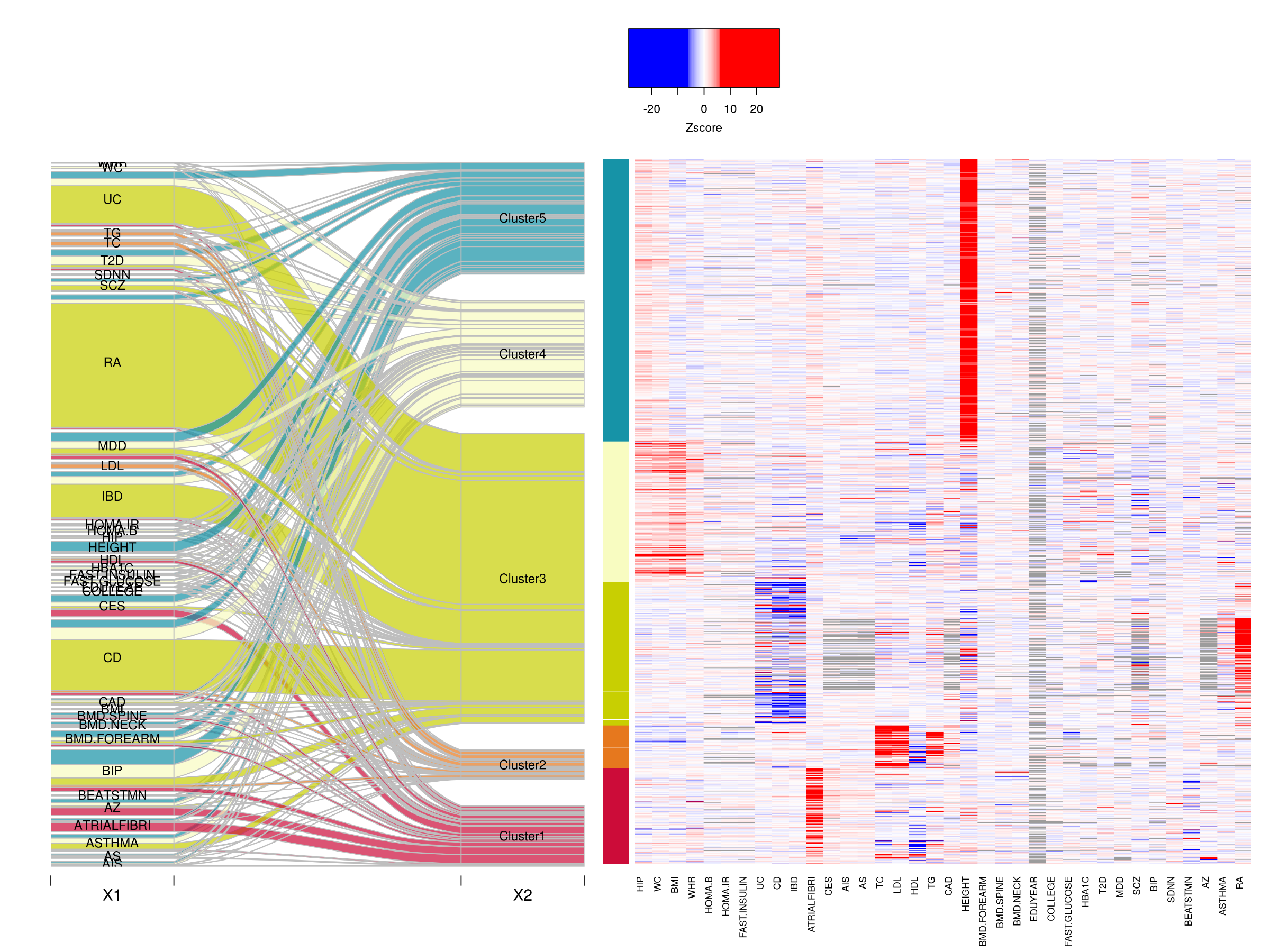

Supplement: S31 Fig — On the left panel, the alluvial plot represents the re-assignment of SNPs from univariate analysis to clusters. To emphasize the relative genetic contribution to phenotypes, SNPs from each phenotype block were weighted by their variance explained to that phenotype. On the right panel, the heatmap represents the multi-trait signatures. Each line is a SNP, each column, a trait. The gradient of color represents the strength of the Z-scores. (TIF) [file pgen.1009713.s032.tif]

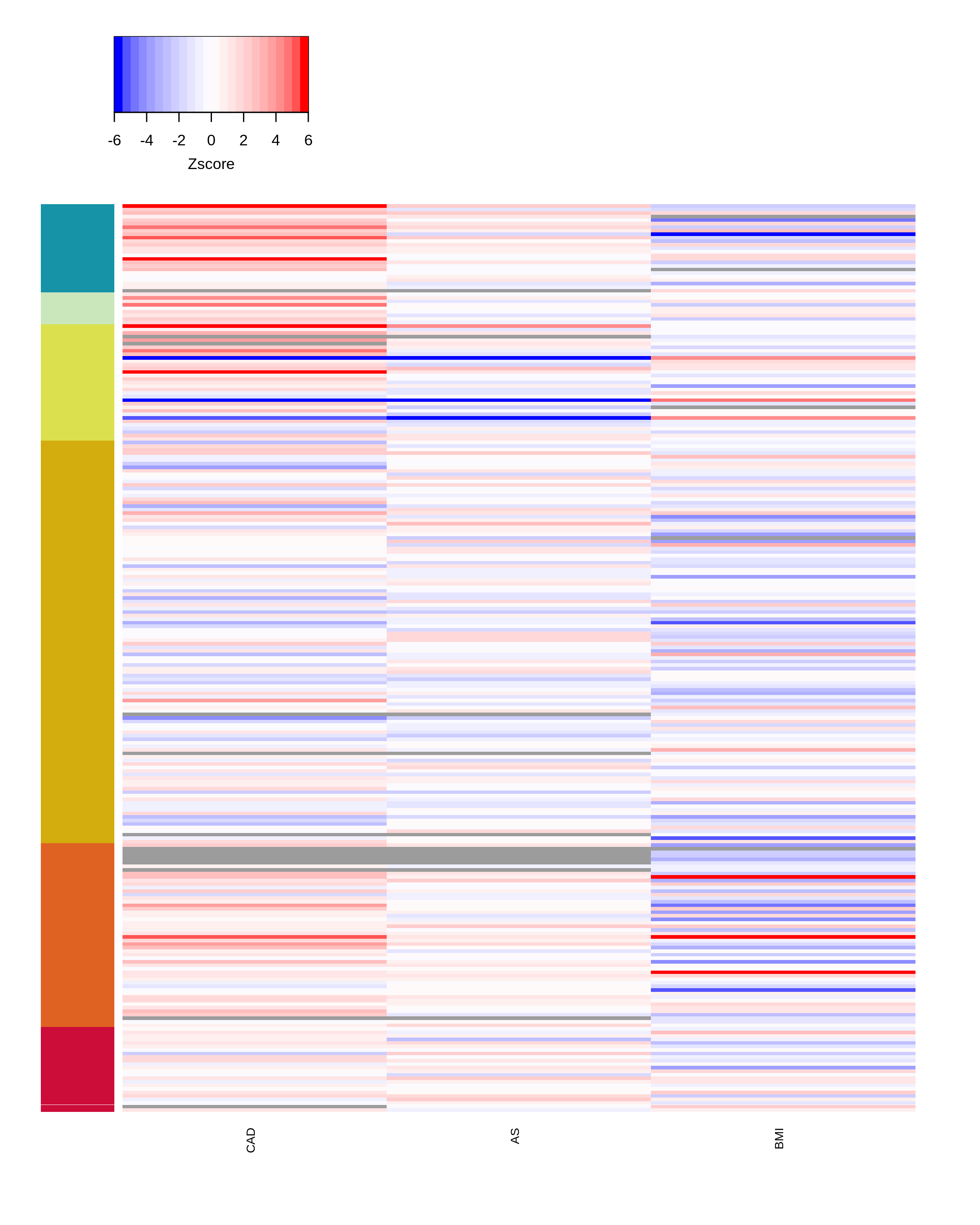

Supplement: S32 Fig — the heatmap represents the impact multi-trait signatures detected on the METABOLISM set on disease that have been linked to hyperlipidemia and diabetes. Each line is a SNP, each column, a trait. The gradient of color represents the strength of the Z-scores. (TIF) [file pgen.1009713.s033.tif]
