# Supplementary material for: Multitrait GWAS to connect disease variants and biological mechanisms
Source: PLoS Genet. 2021 Aug 30;17(8):e1009713. doi: 10.1371/journal.pgen.1009713 (PMC8437297; doi:10.1371/journal.pgen.1009713)
Supplement: Attachment — Submitted filename: Review1_v0.4.docx [file pgen.1009713.s055.docx]

**Please address all reviewers' comments including Reviewer 1's comments on clarifying whether the biological and clinical insights obtained from this work are meant to be suggestive or whether the authors believe they are strong enough on its own to guide future action / decision making.**

**###Reviewer #1 ##################################**

**This paper includes a lot of analyses of summary-statistic GWAS results for multiple traits, and partitions SNP associations into clusters. There are interesting methodological details, and fairly detailed analyses and interpretations of several sets of phenotypes, with extensive supplemental material. The manuscript is written to focus on the phenotypic results with the methodological work largely in the supplement. It might be better to include more of the methods details in the main text.**

We thank the reviewer for the thorough assessment of our work. And the reviewer is right that we decided to move all details of the methods in the supplement in order to focus on the results, but also to limit the length of the main text. Following the reviewer’s suggestion, we highlighted a part of the validity and sensitivity study we conducted on each test in the main (see the *Multitrait genetic association test* Results section).

**The Introduction is not well written and needs more clarity and rationale. In fact the paper is comparing a set of multivariate methods, including at least one new method, and evaluating the performance and the identified results. But this message does not come through clearly at all in the current introduction. Also, the introduction has a lot of space denoted to methods & results. I know this is a common practice in many articles, but in this case, the sentences’ meaning is unclear. The introduction should spend more time on the motivation. For example: “First, characterizing and comparing the relative performances of alternative multitrait association models, we found strong specificity of the signal identified by each approach,…” These “alternative…models” are not mentioned. Different kinds of models? Different assumptions? Why where they chosen? Then you say “…identified by each approach” but we don’t know what the approaches are.**

We made an overall revision of the introduction, which we hope, state more clearly the existent, the motivation for our study, and the goal we are trying to achieve. The second paragraph now reads:

*“The application of multitrait association tests to a large heterogeneous set of traits requires overcoming several practical issues including careful preprocessing of individual GWAS summary statistics to avoid statistical artifacts, the estimation of multiple global parameters, and addressing widespread missing summary statistics. We addressed these issues in recent studies, developing the RAISS^27^ approach for imputation, and JASS preprocessing and multitrait analysis pipeline software^28^. Nevertheless, the relative performances of existing multitrait tests in real data have not been fully addressed. In brief, two types of methods have been developed, weighted sum of univariate statistics, assuming a specific multivariate genetic effect distribution^12,15,29^, and an omnibus approach, allowing for one degree-of-freedom per statistic ^11,30^, with some approaches using a combination of both31. An extensive and fair comparison of these methods is challenging as most face some of the aforementioned practical issues, no readily available implementation28, and power in real data highly depends on the true (and unknown) multitrait genetic effect distribution. Finally, in addition to the potential ability to detect new variants, there is increasing interest in using GWAS multitrait association to decipher inter- and intra-phenotype genetic architecture^32,33^. Again, real data applications are scarce, and questions remain regarding on the approach to be used, the detectability of the multitrait genetic structure behind genome-wide genetic correlation, their potential matching to biological mechanisms, and their potential clinical utility.”*

**Results. Performance of the different multivariate methods are discussed in the main text – when they agree, when they disagree, what kinds of features they agree on. But it is done superficially rather than exploring the specific assumptions behind each model. Presumably, a more in-depth comparison can be achieved through the simulation studies, but none of this appears in the main text. For example, are each SNP effects on phenotypes correlated? Or are the phenotypes correlated because the SNP acts on a shared latent factor that increases risk of each phenotype? Or are the phenotype correlated due to residual (non-genetic) correlations?**

We initially decided to focus on real data analysis only, as the relative performances of each model considered depends on too many parameters for an exhaustive comparison. Nevertheless, we understand the reviewer’s concerns, and simulations might indeed be informative to assess the key characteristics of the tests considered. We extended the main text with a description of the results of two simulation study. The first one presented on Fig. S14 is a pedagogical simulation designed at representing the significance boundary of each test on simple bivariate examples. We chose to use two traits for the ease of representation of significance boundary and though it would help the reader to grasp intuitively the difference between the tests. We then studied the statistical power the *omnibus* test compared to the *SumZ_g_* test for more realistic simulation scenario (10 traits). The results of this simulation study are laid out on the new Fig. 1. The main text now reads:

Results : “*Next, to illustrate the relative detection ability of each approach, we conducted a series of simulation studies across various scenarios.* ***Fig S14*** *represents the null hypothesis rejection boundaries of each test using a simple example with 2 traits, when the genetic effect follows a single bivariate normal distribution and when it follows a mixture of two bivariate normal distributions (to reflect our working hypothesis). In this setting, the omnibus test displays the largest detection rate. Among the sumZ tests, sumZ_g_ and sumZ_ica_ show better performances than sumZ_1_ and sumZ_r_, especially when the structure of the genetic signal is heterogeneous. We then conducted more extensive simulations focusing on the omnibus and SumZ_g_ tests.* ***Fig 1*** *lays out the results of simulations using 10 traits generated under contrasting scenarios. Overall, the omnibus test performs better than the sumZ_g_ test when all traits have a high heritability and if the genetic signal is not structured along a specific direction. The statistical power of the omnibus test also tends to increase with the sample overlap especially when the environmental correlation was not aligned with the genetic correlation. Interestingly this good performance was also observed for genetically uncorrelated traits when they have a high heritability. In the case of a highly structured genetic effect sampled along a specific direction SumZ_g_ and the omnibus test performed similarly. However, when only one of the 10 traits had a high heritability, the omnibus test underperformed compared to the SumZ_g_, reflecting the cost of additional degrees of freedom in the omnibus test.*”

Material and Methods:

“*To assess the statistical power of the Omnibus test and the SumZ tests, we designed the following simulation scenarios. A 10 trait z-score vector was generated for each of the 5000 causal SNPs as the sum of a genetic effect and a residual effect: Z= Z_g_ + Z_res_. In the following, random covariance matrices were generated using the randcorr R package^78^. Z_res_ was sampled from a normal multivariate distribution with covariance matrix (*$\boldsymbol{\Sigma}_{r}$*) terms equal to*$\sigma_{z}={\rho n_{s}}/{\sqrt{n_{1}n_{2}}}$ *where* $n_{1}$*is the sample size of the first study,* $n_{2}$ *is the sample size of the second study and* $\rho$ *is the phenotypic correlation among the* $n_{s}$ *overlapping samples. As demonstrated in^9^,* $\rho= \rho_{g}+ \rho_{e}$ *where* $\rho_{g}$ *is the genetic correlation and* $\rho_{e}$ *the environmental residual correlation. We generated the residual covariance matrix* $\boldsymbol{\Sigma}_{r}$ *according to two scenarios: (random)* $\rho_{r}$ *was randomly sampled with the only constraint that* $\boldsymbol{\Sigma}_{r}$ *remains a semi definite positive matrix, (aligned)* $\rho_{r}$ *was constructed as* $\rho_{r}=0.4 \times\rho_{g}+0.6 \times\mathcal{E}$ *with* $\mathcal{E}$ *sampled at random. Z_g_ varied depending on the simulation scenario: (eff ~ Random) Z_g_	was sampled from a uniform multivariate distribution with boundary*$\left⟦ -6, 6 \right⟧^{10}$*, (eff ~ corG) Z_g_	was sampled from a normal multivariate distribution with a random covariance matrix, (eff ~ wG) Zg was sampled along a straight line blurred with a normal noise, (eff ~ high H), Z_g_	was sampled from a normal multivariate distribution simulating genetically uncorrelated traits with high heritability , (eff ~ het H), Z_g_	was samples from a normal multivariate distribution simulating genetically uncorrelated trait with only the first having a high heritability.*”

**Many interesting findings are buried in one sentence: e.g. “We also developed corrections for several critical real data issues related to model misspecification (Figs S7 to S12) and missing data (Fig.S13)”. Some of these results should be in the main body of the paper. Similarly, in Methods, you say briefly “we implemented additional tools to estimate the per SNPs sample size…”. In the relevant supplemental material, the formulas and Figures relating to sample size are shown without sufficient explanation. It is difficult to decipher what is the problem being addressed in these supplemental sample size sections.**

Following the reviewer suggestion, we moved some of the methods and supplementary notes related to the correction of the issues identified in the results section, adding a full paragraph that described in more details the motivation and the proposed solution. The main text now reads:

*“We first performed an in-depth validation of each approach, starting with series of simulation under an ideal situation, when there are no missing data and the true Z-score covariance matrix, Σ_r is known (Figs S1 to S3). We further show using both simulated data (Figs S4 to S5 and Supplementary Note) and real data from the UK Biobank cohort that in the specific case of complete sample overlap between GWAS, the omnibus test is asymptotically similar to a Multivariate Analysis of Variance (MANOVA) applied to individual level data (Figs S6 and Supplementary Notes). The only major potential source of bias we identified is the misspecification of Σ_r which can lead to severe type I error inflation (Figs S7). Misspecification can affect all variants, if for example Σ_r is estimated naively using the z-score data as proposed in previous studies (Fig S8). Comparing various approaches, we found that Σ_r can be accurately estimated using the LDscore regession9 (Fig. S9), which was therefore used to estimate Σ_r along the genome-wide genetic correlation (Σ_g) for the 36 phenotypes analyzed (Tables S2 and S3). However, misspecification can also be SNP-specific when sample size varies across the SNPs analyzed. Per-SNP sample size heterogeneity can induce different proportion of sample overlap, and therefore variability in pairwise residual correlation. We illustrate this potential bias by applying the omnibus tests for the joint analysis of the four GWASs from the GC consortium^36^ (Fig S10). To address this issue, we implemented additional tools to estimate sample size per SNP when missing, and subsequently filter the variants with small sample size (Figs S11, S12 and Supplementary Note). Finally, out of 10 million variants reported for some GWAS, fewer than 1,000 had complete summary statistics for all 36 phenotypes analyzed. While methods exist to impute missing GWAS statistics, they appear inaccurate for multitrait analyses and we implemented the RAISS^37^ approach we recently developed to ensure valid imputation for our context (Fig. S13). All pre-processing steps were recently incorporated into a publicly available toolset38. After applying our pre-processing pipeline to all 36 GWAS analyzed, there remained 6,978,319 SNPs with a missing data rate of 45% (59% before imputation).”*

We also updated the supplementary, adding context and objective and revising some of the formulation. We hope this address the reviewer comment.

**Existing multivariate methods were cited, but not used. Is there a reason for this?**

Actually, several of the approaches used in this study are similar in principle to some of these existing approaches, but re-implemented because their original implementation was either not usable or there was simply no implementation. An overview of these limitations is available in a previous publication (Julienne et al, 2020). Overall, it highlighted limited documentation, the absence of data pre-processing, and the inability to accommodate for missing value (note that even after imputation, missing data remains substantial and has to be addressed to allow for real data application). We now clarify in the introduction that the tests used are similar in principle to some of the existing approach and provide additional details on the links between the approaches used and existing one in the supplement:

Introduction: “*We implemented five tests, an omnibus K degree of freedom test (for K GWAS analyzed jointly) not assuming any prior as in ^11,29^, and a weighted approach using four alternative weighting schemes, including some partly similar to previously proposed ones ^12,14^*”

Supplement (about the omnibus test): *“This test is similar to the previously published metaCCA(1) approach. However, the existing implementation of metaCCA does not address any of the practical issues discussed in the main text (data pre-processing, missing statistics, etc). It also shares similarity with TATES(2), which suffers the same practical issues as metaCCA. Moreover, TATES has a slightly different null hypothesis, based on the aggregation of p-value instead of signed statistics. Finally, both methods propose using an ad hoc estimation of the covariance between test under the null, which, has shown in Fig S8, can be invalid.”*

Supplement (about the sumZ test): *“Several previous publications presented weighted approaches with various flavors (e.g.^3-6^). For example, the HIPO^3^ approach propose to optimize the weights by combining Σ_r and Σ_g, the genetic and residual covariance matrix, respectively, when we considered them separately. Zhu et al proposed two alternative weighting schemes, referred to as sHom and sHet. The sHom approach^7^ is very similar to the weighting scheme of 1, assuming similar direction of genetic effect across the traits analyzed, while also accounting for sample size per GWAS. On the other hand, the sHet propose a binary weight (i.e. 0 or 1) allowing to include or exclude a subset of phenotype to be analyzed jointly. Finally, the aSPU test^5^ and its extension (e.g. metaUSAT^6^) extend the latter sHet approach and proposes using a combination of multiple weights. While potentially powerful, the ad hoc combining assessing multiple weight requires estimating the null distribution of the statistics using a Monte Carlo simulation.”*

More generally, this paper focusses on assessing the performances of weighted statistics and omnibus tests in real data and on the biological interpretation of derived results. We hope that the overhaul of the introduction has clarified this point.

**Clustering: I am sure the authors are well aware of the challenges involved in interpreting clusters. Here, only SNPs that showed significance at 10-8 with at least one method are included in the clustering. Bootstrapping was used to look at cluster stability w.r.t. the number of clusters and the BIC/Silhouette criteria, but these would all be different if another threshold were used. Some clusters would have to be found given the thresholding effects. In fact, the authors acknowledge (in some way) the ad hoc nature of their analyses when they say: “These distinct multitrait association profiles might arise because their variants belong to distinct genetic functional groups. Understanding whether those genetic functional groups are only statistical construction or correspond to meaningful biologically mechanism is critical. In the latter, it means that data-driven approach, such as the one proposed in the present study, can be used to dissect the genetic contribution of many complex human phenotypes.” But this crucial text is in the middle of results, whereas I think this needs to be highlighted in the introduction.**

We agree with the reviewer that this important acknowledgement should not be buried in the result section. However, discussing this limitation in the introduction would require extensive context, and we therefore only briefly mention this in the introduction, while moving and adapting the cited text in the discussion.

Introduction: *“Again, real data applications are scarce, and questions remain on the approach to be used, the detectability of multitrait genetic structure behind genome-wide genetic correlation, their potential matching to biological mechanisms, and their potential clinical utility.”*

Discussion: “*The question of substructures underlying genome-wide genetic correlation has been partially explored in other recent studies^8,10^. Our work is in agreement with these studies, confirming the presence of regional genetic correlation differences and offering a data-driven approach for identifying primary substructures across millions of possibilities. Nevertheless, understanding whether those genetic functional groups are only statistical construction or correspond to meaningful biologically mechanism is critical. In the latter, it means that data-driven approach, such as the one proposed in the present study, can be used to dissect the genetic contribution of many complex human phenotypes. Here, we used two complementary functional enrichment analyses to map these multitrait association profiles to pathways, and report a detailed view of these profiles for the immunity and the metabolism phenotype set*.”

**An important message that needs to come through better is whether the conclusions that can be obtained through this kind of multivariate analysis of summary GWAS data is comparable enough to multivariate analyses of individual-level data to enable making decisions about potential drugs or treatments. Or is it just suggestive.**

Our response is two-folds. As we demonstrated in Fig S4-6 and supplementary note, in term of statistical power, the omnibus test based on summary data is equivalent to a MANOVA conducted on individual-level data when the number of samples is largely superior to the number of traits. There is therefore no expected limitation in using summary statistics as compared to individual-level data when inferring genetic structure from standard frequentist parameters. Using individual-level to implement more advanced methods (e.g. Bayesian method as in *mvBimBam*, Shim et al 2015) might allow to derive more parameters, but would be limited in sample size and in the number of phenotype that can be analyzed jointly because –as mentioned in the introduction and conclusion— of ethical and practical issues in obtaining and assembling large-scale individual-level data.

For the second point –using these results for making decisions about potential drugs or treatments– we have now updated the discussion to precise that the retrospective analysis on drug target is only suggestive about the potential deleterious side effect of a drug target. Yet considering the tremendous cost of drug development, we believe these results are still of high interest for clinical purposes. The discussion now reads:

“*Note that such retrospective analysis can be only suggestive of the potential deleterious side effects of drug targets. Nevertheless, the identification of those candidates might limit cost of further analysis using both in silico analyses in independent data, and ex vivo to examine the role of the identified genetic variants on additional intermediate molecular phenotypes (gene expression, protein, etc).*”

**Could some UKbiobank data be used to do some structural equations modelling including individual level data, and to confirm some of the clustering relationships seen? i.e. could directed graphical relationships be estimated in the individual level data?**

We fully agree with the reviewer that SEM can be an interesting path forward, and we started investing that option in preliminary studies. However, the method development/adaptation required is substantial and is, in our opinion, be a full study by itself. We therefore decided not to mention SEM in our study.

**Minor**

**Careful editing would be helpful to clean up prepositions and articles, as well as general spelling and grammar here and there. A few examples are provided here:**

- **“relevance of multitrait association tests, there have” : probably should be “they”**
- **“We performed a series of analyses”**
- **“To understand further the relative performance of those three tests (omnibus, sumZica, sumZg) along with the univariate test”**

**And general wording would also benefit from good editing. For example:**

- **“we explored which multitrait signal was associated with the largest increase in detection per test. For that aim,…” The use of “For that aim,…” here is awkward.**
- **“…median chi-squared were elevated for the any …” should be “median chi-squared tests were elevated for the any…**
- **Supplement, section on “Theoretical comparison with MANOVA”:**
- **K>>N should be K<Clustering: Fig S22 should indicate the number of clusters chosen.**

We thank the reviewer for his careful reading of the manuscript. We’ve done the requested changes, and paid editorial services from *Nature Research Editing Service* to revise our manuscript.

**### Reviewer #2 #########################**

**This study tackles the problem of finding pleiotropic loci that affects multiple traits (multi-trait analysis) and interpreting the complex genetic effect patterns. This is an important problem in the current genetics field, I think. The paper is extremely well written and most of the parts can be easily understood. The analyses and figures are fascinating. I only have a few minor comments.**

We thank the reviewer for his/her kind appreciation of our work.

**The authors are quite modest and do not argue that they developed these methods: the various sum of squares and omnibus. (They all look quite straightforward, and leave no room for controversy) Although omnibus is quite widely used, I think there’s some novelty in using PCs (driven from various sources) as weights for FE-meta. It’s a pretty simple idea, but it’s important to note that it’s certainly not what typical geneticist can bring up from his/her head instantly. I mean, it’s a good idea. I couldn’t find any citation there? So, if using PC as weight direction is what the authors have developed, I think it’s OK to advertise it as it is. (including the independent component analysis part)**

We thanks the reviewer for his/her enthusiasm for our weighting scheme. The simulation study added to answer reviewer 1 request, clearly highlight the advantage of using a weighting based on the genetic correlation. We emphasize further this point in the Result section (see the *Multitrait genetic association test* Results section:

Results: “*Next, to illustrate the relative detection ability of each approach, we conducted a series of simulation studies across various scenarios.* ***Fig S14*** *represents the null hypothesis rejection boundaries of each test using a simple example with 2 traits, when the genetic effect follows a single bivariate normal distribution and when it follows a mixture of two bivariate normal distributions (to reflect our working hypothesis). In this setting, the omnibus test displays the largest detection rate. Among the sumZ tests, sumZ_g_ and sumZ_ica_ show better performances than sumZ_1_ and sumZ_r_, especially when the structure of the genetic signal is heterogeneous.”*

**A bit of discussion about possible multiple testing correction issue for applying combination of Z-r, Z-ica, and omnibus together would be beneficial.**

We thank the reviewer for raising this important point. As performed on the same data and with partial similitudes in the weighting scheme, the 5 statistical tests cannot be considered independent. Hence, a correction for multiple testing is not completely straightforward. To address this issue, we performed our joint analysis using a conservative Bonferroni p-value threshold: 1x10^-8^. We agree with the reviewer that this was not stated clearly in the previous version of the text. The main now reads:

Result ‘Comparison of Multitrait genetic association signal’ section: “*We applied a bonferroni correction to the joint tests and used a p-value threshold of 10^-8^ instead of the standard 5x10^-8^.”*

In complement to a stringent threshold, we filtered out associations reported in the GWAS catalogue at the time of the first submission aiming at a conservative assessment of the potential of multi-trait testing to discover new association.

Discussion: *“Hence, at the date of the publication of this paper, newer summary statistics are available for few traits. We accounted for these new publications when counting newly identified variants by filtering associations reported in the latest version of the GWAS catalogue.”*

**Although details of GMM / MGMM are not described, only citing biorxiv paper, it should be better to reiterate how these frameworks work in a brief version here, so that this paper can be self-contained.**

We initially refrained from explaining the MGMM algorithm in the paper in the sake of concision. Yet, we believe, as the reviewer, that a brief overview of the method rationale and assumptions would benefit the reader. To this end, we extended the Material and method section in the main:

“*Briefly, the assumed generative model is as follows. Consider n independent vectors* $Z_{i}=\left( Z_{i1}, \ldots,Z_{ij},\ldots,Z_{id} \right)^{t}$ *in* $\mathbb{R}^{d}$*, each arising from one of k distinct clusters. Each vector represents one SNPs. The value* $Z_{ij}$ *is the Z-score of SNP* $i$ *on trait* $j$*. Let* $I_{ij}= 1$ *if the* $i^{th}$ *observation belongs to cluster* $j$*, and define the indicator vector* $I_{i}=\left( I_{i1}, \ldots,I_{ij},\ldots,I_{ik} \right)^{t}.$ *Conditional on membership to the* $j^{th}$ *cluster,* $Z_{i}$ *follows a multivariate normal distribution, with cluster specific mean* $\mu_{j}$ *and covariance* $\Sigma_{j}$*. Let denote* $\pi^{j}$ *the marginal probability of membership to the* $j^{th}$ *cluster. The observations can be viewed as arising from the following hierarchical model:*

$$I_{i} \sim Multinomial(1, \pi)$$

$Z_{i}\left( I_{ij}=1 \right)=N(\mu_{j} , \Sigma_{j} )$*”*

*This method relies on EM optimization techniques enabling the inference of unobserved variables from observed variables and an assumed Gaussian mixture model. In classical GMM, the only variable inferred is the posterior probability cluster membership. In the MGMM algorithm, missing Z-scores are also inferred taking into account the observed Z-scores and the inferred probability to belong to a cluster*.”

**I would be really interested if the code that can automatically perform (1) some kinds of standard preprocessing including summary imputation, (2) various tests, and (3) visualization of Figure 2, 3, and 4. (Or generation of input formatted-data for Figure 2,3,4 along with the standard script). That will really help the community.**

We thank the reviewer for her/his interest in JASS code. We believe that statistical methods should be made available to the genetic community in an automated and in a user-friendly way. To this end, we developed a pipeline enabling the automatic pre-processing of summary statistics, the computation of the covariance matrix under the null hypothesis, and their imputation. This implementation is described in a previous paper focusing on software development ([Julienne et al, 2020](https://academic.oup.com/nargab/article/2/1/lqaa003/5715214)).

While able to automatically manage a large set of summary statistics, this pipeline requires a computation cluster and a substantial configuration effort. To enable users to conduct a multi-trait GWAS with minimal effort, we set up a public server running a JASS application ( <https://jass.pasteur.fr/index.html>). Currently, on this server, users can run the omnibus tests by selecting traits among 154 public summary statistics.

The visualisation of Figures 3, 4, 5 (new numbering) would require a substantial amount of supplementary work to be fully automated that would justify a supplementary publication. To facilitate the reproduction of similar figures we list below, and in the manuscript, the R packages used for each figure (note that the numbering has been updated):

Figure 3 : UpsetR, radarchart

Figure 4 : alluvial, gplots

Figure 5 : igraph

**###Reviewer #3: Review: Multitrait GWAS to connect disease variants and biological mechanisms**

**Overview. Julienne et al integrate GWAS summary statistics from multiple phenotypes to provide insights into shared genetic architecture and biological mechanisms. They leverage previously described multi-trait association methods to identify GWAS signals shared across 36 phenotypes and apply a novel clustering approach to group underlying association signals into broad biological categories. They highlight results from immune- and metabolism-related phenotypes to shed light on shared pathways. The methods and analyses are rigorous, and the takeaways should be of broad interest to the statistical genetics community. I found the manuscript to be well written, underlying assumptions thoroughly tested with simulations, and results to be generally well supported. With that said, I have a few comments.**

We thank the reviewer for his/her appraisal of our work.

**Major Comments**

**1. Integrating multiple GWAS data from varying cohorts and studies requires great care in handling allele coding, varying sample size, and missingness. The authors have clearly spent a good deal of time considering the practical challenges and I was impressed with their systematic approach to each issue with rigorous statistical modeling and accompanying simulations. Similarly, I appreciated the thorough step-by-step derivations in the supplementary material. It was clear this was a considerable effort on part of the authors and sets a standard for how supplementary methods should be presented and for that they should be commended.**

We thank the reviewer for his/her appreciation of our effort to ease practical challenges of multi-trait analysis for the statistical genetic community. The technical aspects of this paper focuses mainly on the statistical validity of the tests. We previously published a paper solving some of the computational practical challenges raised by multi-trait analysis ([Julienne et al, 2020](https://academic.oup.com/nargab/article/2/1/lqaa003/5715214)).

**2. The authors note the need to prune SNPs that lacked a clear cluster assignment using large values of entropy as a metric. Can the authors perform analyses to provide some indication as to why these SNPs were unable to be assigned to any cluster with high confidence? Is this the result of similar functionality across clusters [and thus the method cannot assign to any with certainty], or is it more likely due to non-shared functionality and lack of a representative cluster?**

This is indeed an interesting question, and we agree with the two reviewer’s suggestions. It actually fits the two limitations of the clustering approach we implemented: 1) the GMM model assumes each SNP belong to a single cluster (and therefore does not account for “similar functionality across clusters”), and 2) the number of clusters is determined empirically (and therefore might fail to capture some cluster –i.e. “the potential lack of representative cluster”). The non-overlap assumption was used because it considerably reducing the space search and thus simplifies the inference of potential multitrait genetic structure. However, it is likely unrealistic for at least some of the variants, and future research using alternative modelling might be able to overcome this assumption. The potential lack of representative cluster is more complex, and might either reflect a power issue (i.e. there is not enough SNPs from a cluster to allow for a reliable inference), or as for point 1) represents a cluster hard to infer using the GMM.

It is challenging to determine whether the SNPs that couldn’t be assigned to a specific cluster results from point 1) or 2). Nevertheless, we have expended the result to clarify these two points:

“We assessed the uncertainty in cluster assignation by deriving the entropy for each variant (see **Supplementary Material**). We observed some heterogeneity in the distribution of entropy values across phenotype sets and clusters (**Fig 4C, Supplementary Tables S16-S17**). The difficulty to attribute a cluster for certain variants might be due to the lack of representative cluster (*i.e.* sub-structure not captured in our analysis) or to shared functionalities between clusters that are not modelled in the GMM framework. Because differentiating those two possibilities using the available data would be very challenging, we decided to remove outlier variants (*N*=28 across all sets) with entropy above 0.75 from further analyses.”

**3. With clusters acting as proxies for biological pathways, it would be interesting to see if tissue enrichment varies across clusters for a specific phenotype group.**

We investigated the tissue enrichment by clusters with two methods. One directly derived from FUMA and based on GTEx expression data and one based on the active chromatin marks (fun-LDA). The results of tissue enrichment are presented in Table 1 and Supplementary Table S13 and discussed in the result section “Biological meaning of genetic clusters.” Interestingly, significantly enriched tissue varies from one cluster to another for the same set of traits. The reviewer remark highlights that this observation was buried among the numerous supplementary table and further emphasize it by editing the text as follow:

Results section:

“Given a phenotype set, while using the same set of traits for all clusters, we observed large differences in pathways, tissues and cell type enrichments between clusters.”

Discussion section:

“In these analyses, the most enriched tissues varied substantially across clusters within each phenotype set, highlighting the potential of such an approach for characterizing distinct genetic mechanisms.”

**Minor Comments**

**1. Figure 3 caption has a formatting or word-to-pdf conversion error, “ -cell function”.**

We thank the reviewer for his/her minute attention to the text. We corrected the legend accordingly.
